# A Sobol-Driven Multi-Objective Whale Migration Algorithm for Engineering Optimization

**DOI:** 10.3390/biomimetics11090642

**Published:** 2026-09-07

**Authors:** Lizhen Du, Dahongnian Zhou, Xiaoshuang Xiong, Min Shen, Hongtao Tang, Lianqing Yu, Fei Fan

**Affiliations:** 1 School of Mechanical Engineering and Automation, Wuhan Textile University, Wuhan 430200, China; 2006111@wtu.edu.cn (L.D.);; 2 Three Dimensional Textile Hubei Engineering Research Center, Wuhan Textile University, Wuhan 430200, China; 3 School of Mechancal and Electronic Engineering, Wuhan University of Technology, Wuhan 430070, China

**Keywords:** multi-objective optimization, whale migration algorithm, Pareto front approximation, engineering design optimization, Sobol sequence

## Abstract

Multi-objective optimization plays an important role in modern design and complex engineering applications. However, achieving an effective balance between the convergence and diversity of Pareto-optimal solutions remains challenging. This paper proposes a Sobol-driven Multi-objective Whale Migration Algorithm (SMOWMA), which extends the Whale Migration Algorithm within a non-dominated sorting and elite-selection framework. A maximin scrambled Sobol initialization scheme is first employed to improve the distribution of the initial population. An archive-guided adaptive Student-*t* flight mechanism is then incorporated into the leader-whale position update to dynamically balance global exploration and local exploitation. In addition, archive crowding information and archive-entry success feedback are jointly used to adjust the search behavior according to both environmental diversity and recent search performance. SMOWMA is evaluated on five widely used multi-objective benchmark suites, namely ZDT, DTLZ, WFG, UF, and CF, using four performance indicators: generational distance (GD), inverted generational distance (IGD), spacing (SP), and hypervolume (HV). The results, together with Friedman tests and Holm-adjusted Wilcoxon tests, demonstrate that SMOWMA achieves competitive overall performance in terms of convergence, diversity, and objective-space coverage, although its relative advantage remains problem-dependent. The practical applicability of SMOWMA is further examined using multi-objective welded-beam design formulations, a bi-objective four-bar truss design problem, and a five-objective car side-impact design problem. The engineering results show that SMOWMA can obtain competitive and stable approximation sets for constrained design problems with different numbers of objectives, supporting its effectiveness and applicability in multi-objective engineering optimization.

## 1. Introduction

Engineering design tasks, in practice, rarely involve a single performance target. More often, several objectives must be considered at the same time, while the feasible design space is further restricted by multiple engineering constraints [1]. In such cases, improving one design criterion usually leads to deterioration in another, so the problem cannot be characterized by a single global optimum. The solution is instead described by a set of Pareto-efficient candidates that reflect different compromise relationships among the objectives [2]. Accordingly, a practical multi-objective optimizer should return, within one execution, a set of solutions that not only approaches the Pareto front well but also covers it with reasonable diversity. This is one reason why population-based methods are often preferred, since they can investigate multiple trade-off regions simultaneously. In engineering applications, evolutionary and metaheuristic algorithms built on elitist selection, diversity control, and archive maintenance have therefore become widely used [3].

The welded beam design problem is widely used to assess multi-objective optimization methods. Its main difficulty lies in balancing conflicting goals, since lower manufacturing cost must be pursued without allowing excessive deflection or stress. The design is governed by four strongly linked variables: thickness of the weld, length of the weld, height of the beam and width of the beam. They jointly determine the structural response and the cost of the design. Constraints over shear stress, bending stress, buckling load, and geometry make the design problem more difficult by focusing significantly on the design space. It becomes difficult to find such distributed trade-off solutions because of the extremely limited feasible region for the objectives to collide. The welded beam problem remains a hard challenge for many optimization techniques because of its nonlinear and coupled nature, especially in terms of their ability to prevent local stagnation and maintain population diversity [4].

The welded beam problem may be tackled by aggregating multiple objectives into a single function by fixing the weight of each objective or weighting methods. This approach is clearly inappropriate for the welded beam design problem, for which the solution involves intense trade-offs and strong nonlinear relationships between cost, deflection and stress, so the preset weightings cannot capture the true Pareto relationship between them [5]. Furthermore, when the problem is reformulated in a single-objective form, it is difficult to generate a whole set of representative Pareto solutions at one run and this weakens its application in practical engineering design. The need for more powerful multi-objective frameworks for such problems is obvious when the welded beam design problem becomes even more obvious with increasing numbers of objectives [6].

Even in multi-objective optimization, simultaneous optimization of all of the objectives cannot be realized due to the conflict among the objectives. Thus, the main objective is to find an approximation of the Pareto front (PF) that presents decision makers with interesting options for the design solution [7]. A powerful optimizer must be able to obtain good convergence towards the PF and good spread of distributed solutions, both aspects being useful for engineering tasks. To tackle these types of problems, various solution approaches have been designed. Among these, dominance-based evolutionary algorithms have emerged, like the Non-dominated Sorting Genetic Algorithm II (NSGA-II) [8], the Non-dominated Sorting Genetic Algorithm III (NSGA-III) [9], and the Strength Pareto Evolutionary Algorithm 2 (SPEA2) [10]. Most of these algorithms are based on the concept of non-dominance sorting and elitist preservation. On the other hand, some indicator-based methods are popular, such as the Indicator-based Evolutionary Algorithm (IBEA) [11], the S Metric Selection Evolutionary Multi-Objective Algorithm (SMS-EMOA) [12], and the Multi-Objective Evolutionary Algorithm Based on Decomposition (MOEAD) [3]. The first two methods solve collaborative scalar subproblems and adopt indicator-based selection, respectively, to improve Pareto front approximation.

Multi-objective optimization brings new powers to the state-of-the-art in several aspects: population cooperation, archive management, decomposition guidance, and diversity control. Representatives include the nature-inspired metaheuristics such as the Multi-objective Red-billed Blue Magpie Optimizer (MORBMO) [13], the Multi-objective Whale Optimization Algorithm (MOWOA) [14], the Mult-objective Ivy Algorithm (MOIVYA) [15], and the Multi-objective Chef-Based Optimization Algorithm (MOCBOA) [16], and the Multi-objective Starfish Optimization Algorithm (MOSFOA) [17]. MOSFOA extends starfish-inspired exploration, predation, and regeneration mechanisms to multi-objective optimization by incorporating elitist non-dominated sorting and crowding-distance-based selection to promote convergence and preserve population diversity. Apart from these, swarm-intelligence-based algorithms exist such as the Adaptive Many-Objective Evolutionary Algorithm (AMOEA) with two archives and entropy triggering [18], the Advanced Multi-Objective Ant Lion Optimization Algorithm (AMOALO) [19], the Multi-Objective Salp Swarm Algorithm (MOSSA) for Dynamic Economic Emission Dispatch [20], the Two-Archive Multi-Objective Grey Wolf Optimization Algorithm [21], and the Cooperative Multi-Objective Particle Swarm Optimization Algorithm (CMOPSO) with dual search strategies [22]. No matter what kind of search methods or population update procedures are adopted, a lot of numerical and engineering problems with certain Pareto-optimal fronts will be difficult to handle unless diversity-conscious selection strategies and good environmental selection are considered [23].

Despite these advances, maintaining a proper balance between convergence and diversity remains challenging in complex multi-objective optimization problems. All search strategies of convergence for single-objective optimization may lead the search to biased behavior and converge towards only some portions of the trade-off regions and reduce coverage of spared or extreme PF regions [24]. In addition, inadequate spatial coverage of the initial population may restrict exploration from the outset, making sparsely represented and extreme trade-off regions difficult to reach. Furthermore, poor preservation of the non-dominated solutions and distribution of non-dominated solutions on the Pareto front result in incomplete and uneven approximations of the PF. A lack of sufficient coordination between population updating and environmental selection during the search process also contributes to stagnation and premature convergence [25]. Likewise, a perturbation mechanism that does not respond to generation progress and archive feedback may either lack sufficient long-range exploration or introduce excessive disturbance during local refinement. Therefore, inheriting the essence of the original optimizer while achieving both convergence and diversity remains a challenge [26].

Recently, Ghasemi et al. [27] developed the Whale Migration Algorithm (WMA) as a single-objective metaheuristic based on the collective migration behavior of humpback whales. WMA has attracted attention because of its unique effectiveness by simulating humpback cooperation mechanisms. This algorithm distinguishes two phases: the exploration phase done by experienced whales and the exploitation phase where the followers replicate by looking at the neighbor while mimicking the leader towards attractive zones. Validation over CEC 2005, CEC 2014 and CEC 2017 test functions shows that WMA exhibits good enhancements in terms of accuracy, efficiency and scalability. Given these promising characteristics, Qi et al. [28] proposed a novel Multi-objective Whale Migration Algorithm (MOWMA), which integrates good point sets and logistic chaotic mapping to initialize the population, applies a Lévy flight strategy during exploration, and adopts a dual crowding degree mechanism in the external archive. The high computational complexity of good point set initialization can significantly increase the computational burden of population initialization as the dimensionality increases while the inherent distribution of the logistic map leads to a systematic accumulation of individuals near the boundaries of the search space. The optimality of Lévy flight is highly dependent on the size of the target region. Although the Lévy flight strategy demonstrates clear superiority in smaller regions, determining the optimal α parameter for the tail of the distribution remains difficult in larger search areas. However, a MOWMA employing the Lévy flight strategy may exhibit relatively weak performance on the DTLZ test suite. Particularly, MOWMA tends to fall into the curse of dimensionality when dealing with high-dimensional many-objective optimization engineering problems.

To make up for this research gap, this paper proposes a Sobol-driven Multi-objective Whale Migration Algorithm (SMOWMA). The algorithm introduces non-dominated sorting (NDS), crowding distance (CD) and an archive-assisted elitist retention mechanism to handle multi-objective optimization problems in benchmark test sets and practical engineering applications. Specifically, the algorithm adopts the maximin scrambled Sobol initialization scheme to improve the spatial coverage of the initial population; at the same time, the adaptive Student-*t* heavy-tailed flight mechanism is introduced in the process of leader whale migration, which is adjusted by generation progress, archive crowding distance and archive-entry success feedback. This enhances the global exploration ability and alleviates the search stagnation.

The main contributions of this work are outlined below:

(1) A new Sobol-driven Multi-objective Whale Migration Algorithm (SMOWMA) is proposed for engineering applications. The algorithm integrates the maximin scrambled Sobol initialization scheme and the whale migration search mechanism into a Pareto-oriented multi-objective framework. In addition, the archive-guided adaptive Student-*t* heavy-tailed flight mechanism regulates the leader whale search according to the archive crowding distance and archive-entry success feedback, so as to balance global exploration and local exploitation.
(2) Verify SMOWMA on classic benchmark test sets such as ZDT [29], DTLZ [30], WFG [31], UF and CF [32]. The ZDT and UF series are mainly used for two-objective problems with different front shapes, the DTLZ and CF series are mainly used for three-objective problems involving higher dimensions and constraints, and the WFG series is used to examine the search ability and scalability of algorithms under complex Pareto-front shapes.
(3) Compare SMOWMA with representative multi-objective algorithms such as MOSFOA, MOWOA, MORBMO, MOWMA, SMS-EMOA, MOEAD, SPEA2, NSGA-II and NSGA-III. The comparative study mainly evaluates the convergence of the results, the diversity of the obtained solution set and the approximation quality of the Pareto front.
(4) Apply SMOWMA to constrained welded-beam design problems with different numbers of objectives. In these formulations, conflicting objectives such as fabrication cost, structural deflection, and stress-based responses are optimized simultaneously under engineering constraints. In addition, a bi-objective four-bar truss design problem is considered to examine the trade-off between structural volume and displacement, while a five-objective car side-impact design problem is employed to evaluate the algorithm’s ability to balance structural weight and multiple occupant-safety objectives.

The rest of this article is arranged as follows: Section 2 introduces the relevant basic theories and problem formulations; Section 3 introduces the Sobol-driven Multi-objective Whale Migration Algorithm (SMOWMA); Section 4 gives the experimental results and discussion of the benchmark test set; Section 5 further evaluates the scalability and engineering applicability of SMOWMA through multi-objective welded-beam design formulations, a bi-objective four-bar truss design problem, and a five-objective car side-impact design problem; and Section 6 summarizes the full text and puts forward the future research direction.

## 2. Problem Formulation

### 2.1. Single- and Multi-Objective Optimization

A conventional single-objective optimization problem involves only one scalar objective to be optimized, typically by minimizing or maximizing the corresponding cost function. The problem can be generally expressed as follows:

(1) $$min_{x\in X}z=f(x)=f(x_{1},x_{2},\ldots ,x_{D})$$

subject to

(2) $$\left\{\begin{array}{l} g_{i}(x)\leq 0,\;\;\;\;i=1,\ldots ,n\\ h_{j}(x)=0,\;\;\;\;j=1,\ldots ,m\\ x^{L}\leq x\leq x^{U} \end{array}\right.$$

where $x=\left(x_{1},\ldots ,x_{D}\right)\in R^{D}$ is the decision vector, $f:R^{D}\to R$ is the objective function, $g_{i}$ and $h_{j}$ are constraint functions, and $x^{L},\;x^{U}$ define the variable bounds [33]. This formulation yields a single optimal solution $x^{*}$ under given constraints.

Compared with the single-objective formulation presented above, multi-objective optimization considers multiple objective functions simultaneously under a common set of constraints. Rather than producing a unique optimum, it seeks a set of non-dominated trade-off solutions for decision-making [34]. The general form of a multi-objective optimization problem is given as follows:

A general multi-objective optimization problem (MOP) is defined by m objective functions, $f_{1}(x),\ldots ,f_{m}(x)$, that are optimized simultaneously within a feasible decision space $X\subseteq R^{D}$. It is commonly written in the form of a vector minimization problem:

(3) $$min_{x\in X}\;f(x)=(f_{1}(x),f_{2}(x),\ldots ,f_{m}(x))$$

When constraints are present, they may be incorporated into the multi-objective model using standard constraint-handling mechanisms, such as feasibility-based rules or penalty functions. In contrast to single-objective optimization, however, a multi-objective problem rarely yields a unique optimum. Its solution is instead characterized by a set of Pareto-optimal candidates, each representing a different compromise among the competing objectives.

For two feasible solutions x,y∈X, x is said to Pareto dominate y, denoted by x≺y, if

(4) $$\begin{array}{l} f_{i}(x)\leq f_{i}(y),\;\forall i\in \{1,2,\ldots ,m\}\\ and\;\exists j\in \{1,2,\ldots ,m\}\;such\;that\;f_{j}(x)<f_{j}(y) \end{array}$$

In other words, x performs no worse than y in every objective and outperforms it in at least one objective [34]. A feasible solution $x^{*}$ is regarded as Pareto optimal (or efficient) when no other feasible solution dominates it. The collection of all such non-dominated decision vectors is called the Pareto solution set (PS), while its mapping in the objective space is termed the Pareto front (PF), defined as

(5) $$PS=\left\{x\in X\;|\;∄y\in X:y≺x\right\},\;PF=\left\{f(x)\;|\;x\in PS\right\}$$

The Pareto front therefore consists of the non-dominated objective vectors corresponding to the Pareto-optimal solutions in the decision space [34]. The purpose of solving an MOP is to approximate this front as accurately as possible, thereby providing decision-makers with a representative set of trade-off solutions.

**Figure 1:**
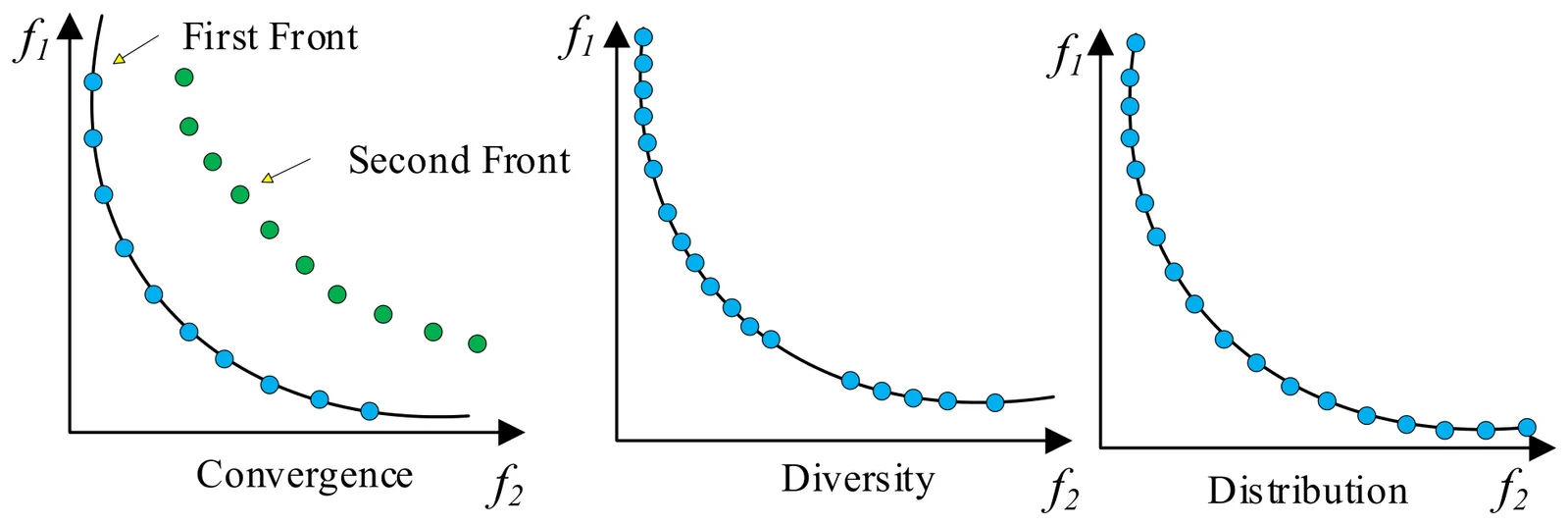
Diagram for convergence, diversity and distribution performance of the MO algorithm.

As shown in Figure 1, the obtained non-dominated solutions are expected to approximate the true Pareto front in terms of convergence, diversity, and distribution. Specifically, convergence measures the closeness of the obtained solution set to the reference PF, diversity reflects the breadth of front coverage, and distribution describes the evenness of solutions along the front. Therefore, the performance of a multi-objective optimizer should be evaluated not only by approximation accuracy, but also by coverage and uniformity. To quantify these properties, the next subsection introduces the corresponding performance indicators.

### 2.2. Performance Metrics

For a quantitative evaluation of multi-objective optimizer performance, two aspects are typically considered: the proximity of the obtained non-dominated set to the true (or reference) Pareto front, and the coverage and distribution of solutions along the front. Consistent with common practice in recent engineering-oriented multi-objective optimization studies, this work adopts four indicators, namely inverted generational distance (IGD), spacing (SP), hypervolume (HV), and generational distance (GD) [35].

Let $PF_{obt}=y_{1},y_{2},\ldots ,y_{N_{obt}}$ denote the obtained non-dominated solution set, and let $PF_{ref}=r_{1},r_{2},\ldots ,r_{N_{ref}}$ denote the reference Pareto front. Here, $N_{obt}$ and $N_{ref}$ are the numbers of solutions in the obtained and reference sets, respectively. The Euclidean distance from a point a to a set S is defined as

(6) $$d(a,S)=min_{s\in S}\|a-s{\|}_{2}$$

where $∥\cdot {∥}_{2}$ denotes the Euclidean norm.

#### 2.2.1. Inverted Generational Distance (IGD)

IGD quantifies the approximation quality of the obtained non-dominated set by averaging the distances from the reference Pareto front to the obtained set. A lower IGD value implies better convergence toward the reference front as well as better coverage of it. When the obtained set fully coincides with the reference Pareto front, the IGD value reduces to zero.

(7) $$IGD\left(PF_{obt},PF_{ref}\right)=\sqrt{\frac{1}{N_{ref}}\sum_{i=1}^{N_{ref}}d{\left(r_{i},PF_{obt}\right)}^{2}}$$

Here, $r_{i}$ is the i-th point on the reference Pareto front, and $d(r_{i},PF_{obt})$ denotes the minimum Euclidean distance from $r_{i}$ to the obtained set $PF_{obt}$. A smaller IGD value indicates better overall convergence and coverage. In the ideal case, IGD=0 means that the obtained set fully coincides with the reference front.

#### 2.2.2. Spacing (SP)

Spacing (SP) is used to measure the uniformity of the obtained non-dominated solutions along the front. For each solution $y_{i}\in PF_{obt}$, the nearest-neighbor distance is defined as

(8) $$\delta_{i}=min_{j\neq i}{\left\|y_{i}-y_{j}\right\|}_{2}$$

and the corresponding mean distance is

(9) $$\;\;\bar{\delta }=\frac{1}{N_{obt}}\sum_{i=1}^{N_{obt}}\delta_{i}$$

The *SP* metric is then given by the standard deviation of these nearest-neighbor distances:

(10) $$SP\left(PF_{obt}\right)=\sqrt{\frac{1}{N_{obt}-1}\sum_{i=1}^{N_{obt}}{\left(\bar{\delta }-\delta_{i}\right)}^{2}}$$

Here, $\delta_{i}$ denotes the nearest-neighbor distance of the i*-th* obtained solution, and $\bar{\delta }$ is the average of all nearest-neighbor distances. A smaller SP value indicates a more uniform distribution of solutions along the obtained front.

#### 2.2.3. Hypervolume (HV)

Hypervolume measures the size of the dominated region in the objective space enclosed by the obtained front and a fixed reference point W. To ensure comparability, the reference point must be specified before the calculation and remain identical for all algorithms and independent runs on the same problem. Mathematically, HV is defined as the Lebesgue measure of the union of the hyper-rectangles formed by each obtained solution and W:

(11) $$HV\left(PF_{obt}\right)=volume\left(\overset{N_{cbt}}{\underset{i=1}{\cup }}V_{i}\right)$$

where $V_{i}$ denotes the hyper-rectangle formed by the objective vector $y_{i}$ and the reference point W, and volume(⋅) denotes the Lebesgue measure of the dominated region. A larger HV value indicates a better approximation set in terms of both convergence and diversity. The construction procedure and the problem-specific reference points adopted in the benchmark experiments are reported in Section 4.1.

#### 2.2.4. Generational Distance (GD)

Generational distance evaluates the convergence quality of the obtained non-dominated set by averaging its distances to the reference Pareto front. Lower GD values indicate that the obtained solutions lie closer to the true front.

(12) $$GD\left(PF_{obt},PF_{ref}\right)=\sqrt{\frac{1}{N_{obt}}\sum_{i=1}^{N_{obt}}d{\left(y_{i},PF_{ref}\right)}^{2}}$$

Here, $y_{i}$ is the i*-th* obtained non-dominated solution, and $d(y_{i},PF_{ref})$ is the minimum Euclidean distance from $y_{i}$ to the reference Pareto front. A smaller GD value indicates that the obtained solutions lie closer to the true Pareto front.

## 3. Sobol-Driven Multi-Objective Whale Migration Algorithm

### 3.1. Whale Migration Algorithm

**Figure 2:**
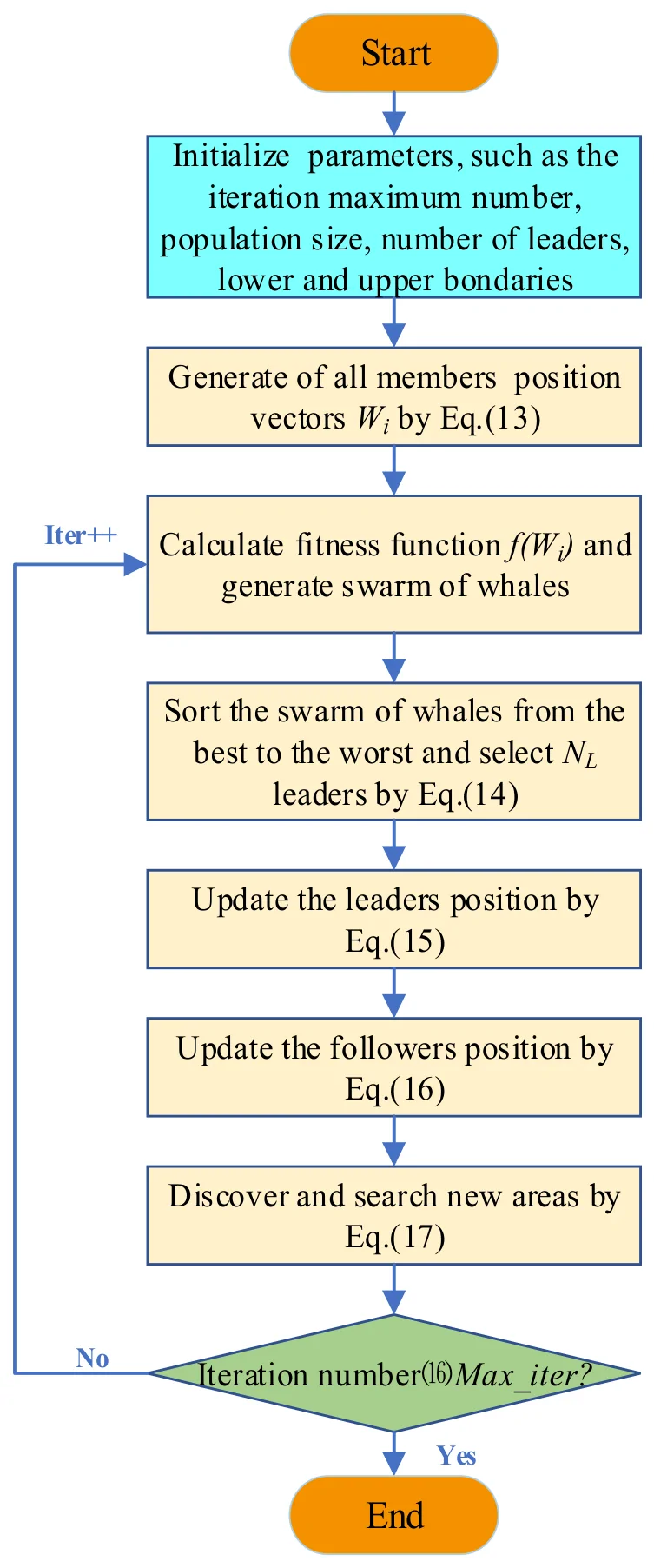
Flowchart of WMA.

The Whale Migration Algorithm (WMA) is a bio-inspired metaheuristic optimization method introduced by Ghasemi et al. [27]. The WMA successfully explores and exploits the search space by mimicking humpback whale migration patterns. The flowchart of the WMA is illustrated in Figure 2.

This subsection describes the whale cooperative migration mechanism and the main procedure of the WMA. First, the algorithm initializes the key parameters, including the maximum iteration number Max_iter, the population size N, and the lower and upper search bounds Land U. An initial whale population is then randomly generated within the range [L,U]. Each whale position, denoted by $W_{i}\left(i=1,2,\ldots ,N\right)$, represents a candidate solution in the search space. The subsequent optimization process is carried out through the following steps:

**Phase 1: Initialization population of WMA.**

A preliminary population or a group of migratory whales and the vector $W_{i}$ are established. This initial production population serves as a collective of migratory whales. The swarm of whales is generated randomly in the search space according to Equation (13).

(13) $$W_{i}=L+rand(1,D)⊙(U-L),i=1,2,\cdots ,N_{pop}$$

where $N_{pop}$ is the number of population members, D is dimension, rand(1,D) is the random numbers from the interval [0,1] and the operation ⊙ signifies the Hadamard product of two vectors.

**Phase 2: Updating current local position of the leader whales.**

All individuals of the population are arranged in descending order according to their fitness value. The movement of each less experienced individual $W_{i}$ is significantly influenced by the previous member in the sequence nearest to them, as described in Equation (14).

(14) $$W_{1},\cdots ,W_{i-1},W_{i},W_{i+1},\cdots W_{Npop}$$

where $W_{1}$ is the best member denoted as $W_{Best}$ and $W_{Npop}$ is the worst member.

In a group of whales, the more experienced individuals known as leaders select the optimal route to the destination, directing the group of whales. Here, the leader whale updates superior locations and the objective function by the following:

(15) $$W_{Mean}=\frac{1}{N_{L}}\sum_{j=1}^{N_{L}}W_{j}$$

where $W_{Mean}$ is the average position of the current leaders and $N_{L}$ represents members with better locations.

**Phase 3: Updating less-experienced whales’ position guided by more experienced whales.**

The *i*th migratory whale $W_{i}$ either endeavors to relocate to a more advantageous site or remains in its current position. If the distance between points $W_{Mean}$ and $W_{Best}$ starts to shorten, it means that the whole group of whales is approaching $W_{Best}$. Finally, the equation for updating the position of the *i*th less-experienced whale is given as follows:

(16) $$\begin{array}{l} W_{i}^{new}=W_{Mean}+rand(1,D)⊙(W_{i-1}-W_{i})\\ \;+rand(1,D)⊙\left(W_{Best}-W_{Mean}\right),i\\ \;=N_{L}+1,\cdots ,N_{pop} \end{array}$$

In Equation (16), the first displacement term describes the local learning of a follower whale from its immediately preceding individual, while the second term guides the follower toward the region determined by $W_{Best}$ and $W_{Mean}$. The candidate position $W_{i}^{new}$ replaces $W_{i}$ only when $f\left(W_{i}^{new}\right)<f\left(W_{i}\right)$.

**Phase 4: Exploration of new search regions.**

The migration of humpback whales was governed by two principles. Initially, the humpback occupies a precise position inside the gravitational and magnetic field, denoted by the vector $r_{1}⊙L$. Secondly, humpback whales navigate in a nearly straight trajectory from the origin point L to the destination U, denoted by the vector $r_{1}⊙r_{2}⊙(U-L)$. Then, the migratory mechanism of the humpback whale is collaborative, as expressed by the following equation:

(17) $$W_{i}^{new}=W_{i}+r_{1}⊙L+r_{1}⊙r_{2}⊙(U-L),i=1,\cdots ,N_{L}$$

where $r_{1}$ and $r_{2}$ are two independently generated D-dimensional random vectors uniformly distributed in ${\left[0,1\right]}^{D}$, while L and U denote the lower- and upper-bound vectors, respectively. Boundary handling is subsequently applied to ensure that $W_{i}^{new}\in \left[L,U\right]$.

After fitness evaluation, the whale population is ranked from best to worst, and the first $N_{L}$ individuals are selected as leaders for the next iteration.

### 3.2. Proposed SMOWMA Framework

**Figure 3:**
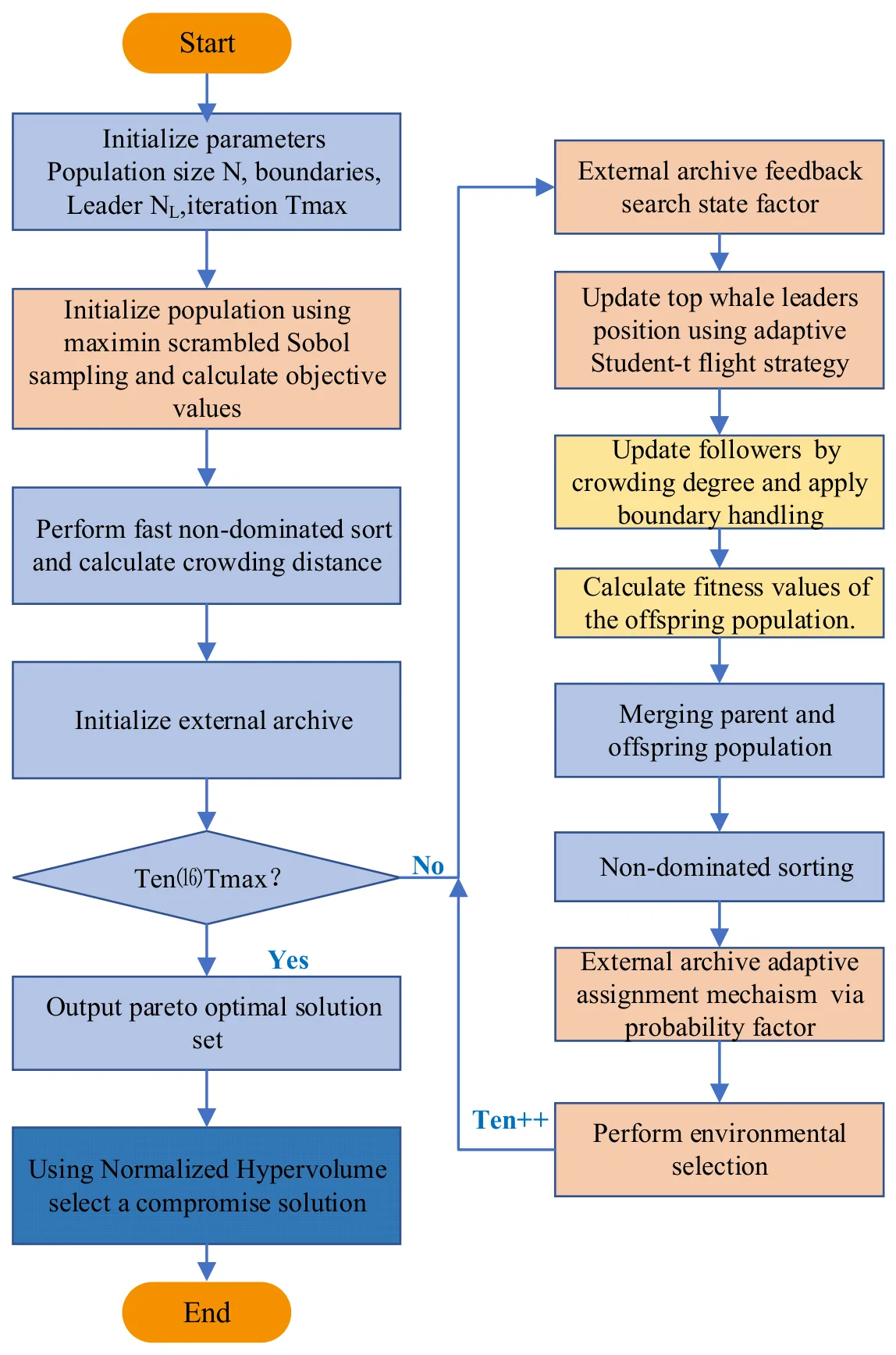
Flowchart of SMOWMA.

The original MOWMA extends the single-objective Whale Migration Algorithm into a multi-objective framework by incorporating the good point set, Lévy flight strategy, and a bi-crowding non-dominated sorting mechanism. The Lévy flight strategy often generates excessively large step sizes, which can lead to the “overshooting” of optimal solutions during the late stages of the search process, thereby degrading the local exploitation accuracy. Despite these enhancements, the original MOWMA often suffers from the curse of dimensionality in many-objective engineering problems, failing to maintain a proper trade-off between convergence and solution uniformity. To overcome these limitations, this paper proposes SMOWMA, a novel variant that integrates Sobol initialization, an adaptive Student-*t* flight strategy, and an external archive mechanism. Specifically, Sobol initialization is employed to enhance the spatial distribution of the initial population, while the adaptive Student-*t* flight strategy dynamically balances global exploration with local exploitation. Additionally, the external archive is utilized to store non-dominated solutions, effectively guiding the search direction and improving the quality of the Pareto-optimal front. The whole flowchart is shown in Figure 3.

#### 3.2.1. Maximin Scrambled Sobol Initialization

In the original WMA, the random initialization defined in Equation (13) often yields clustered individuals, resulting in insufficient coverage of the decision space. Initially, SMOWMA employed a maximin scrambled Sobol sampling strategy to construct a candidate set [36]. The mathematical formation is as follows:

(18) $$C=\left\{z_{1},z_{2},\ldots ,z_{N_{c}}\right\}\subset {\left[0,1\right]}^{D}$$

where $N_{c}$ is the size of the candidate pool and is determined by

(19) $$N_{c}=2^{\left\lceil \log_{2}\left[\max\left(N,\left\lceil kN\right\rceil \right)\right]\right\rceil }$$

where N is the population size and κ is the candidate-pool factor.

Let $I=\left\{1,2,\ldots ,N_{c}\right\}$ denote the candidate-index set. The first candidate index $j_{1}$ is randomly selected from I, and $S_{1}=\left\{j_{1}\right\}$. The remaining candidates are selected sequentially according to the maximin distance criterion [37]:

(20) $$j_{i}=\underset{j\in I\setminus S_{i-1}}{argmax}\;\underset{k\in S_{i-1}}{min}{\left\|z_{j}-z_{k}\right\|}_{2}^{2},\;\;\;\;i=2,\ldots ,N$$

where $S_{i-1}$ denotes the set of candidate indices selected before the ith step. After selecting $j_{i}$, the index set is updated as $S_{i}=S_{i-1}\cup \left\{j_{i}\right\}$. The selected samples are then mapped to the actual decision space:

(21) $$W_{i}=L+z_{j_{i}}⊙\left(U-L\right),\;\;\;\;i=1,\ldots ,N$$

This strategy leverages both the low-discrepancy property of scrambled Sobol sampling and the maximin distance criterion, thereby reducing initial clustering and improving the coverage of the decision space [38].

#### 3.2.2. Fast Non-Dominated Sorting and Crowding Distance

**Figure 4:**
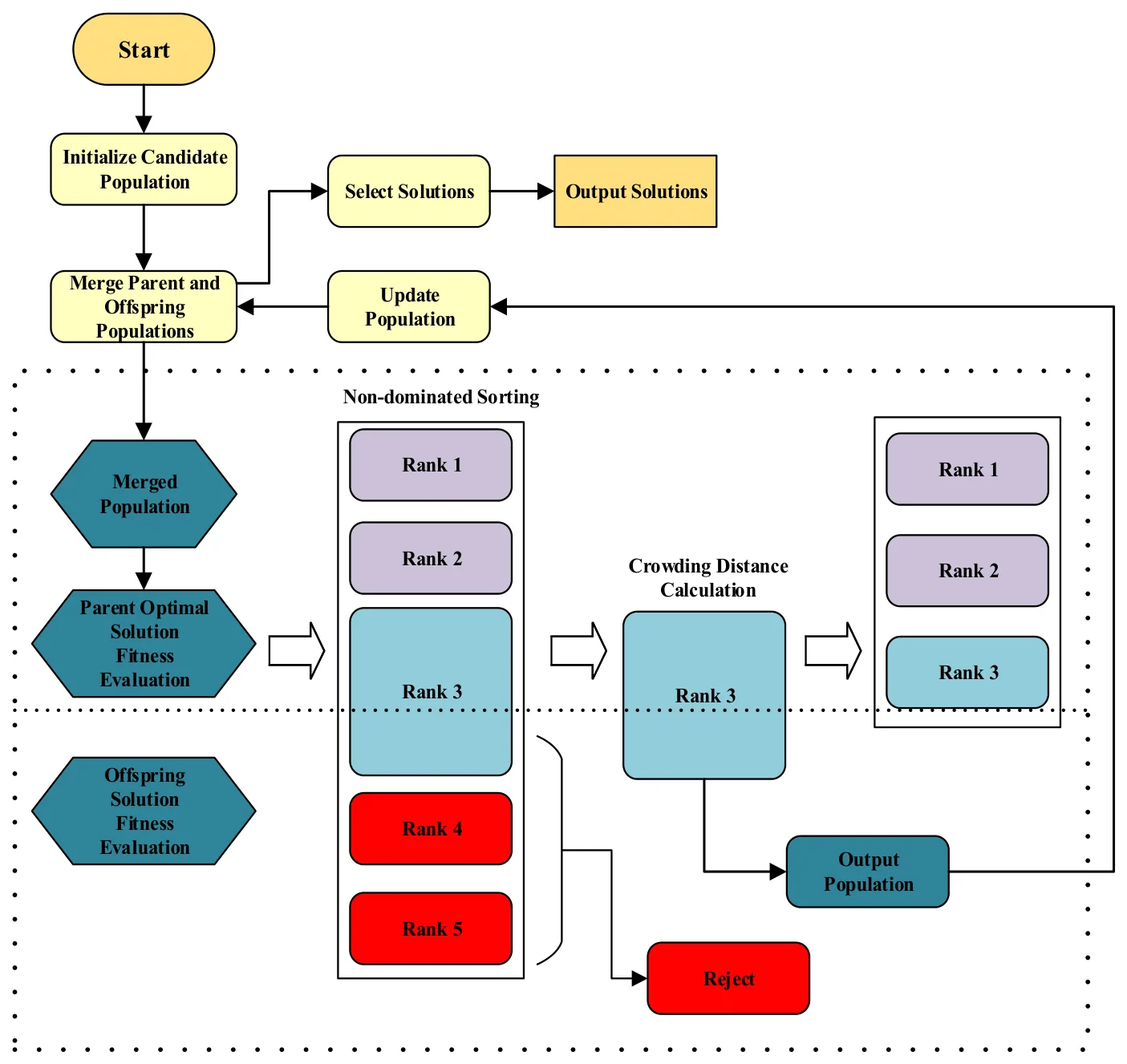
Diagram representation of non-dominated sorting method. The dashed box indicates the NDS/CD-based selection module.

To rank candidate solutions and maintain diversity in the objective space, SMOWMA employs fast non-dominated sorting (NDS) and crowding distance (CD). NDS establishes the selection priority of candidate solutions according to Pareto dominance, while CD estimates the local density of solutions within each non-dominated front. Pareto rank is used as the primary selection criterion, whereas CD distinguishes solutions with the same rank, thereby maintaining the distribution and coverage of the population along the Pareto front. The detailed NDS/CD-based selection procedure is illustrated in Figure 4.

To further alleviate crowding within the same non-dominated front, crowding distance (CD) is introduced as a density estimator and is computed separately for each front during the NDS/CD-based update. For a given front F, the solutions are first sorted with respect to each objective. Boundary solutions are assigned large CD values to preserve extreme points, whereas interior solutions accumulate normalized neighbor gaps across all objectives:

(22) $$CD_{i}\leftarrow CD_{i}+\frac{f_{m}(i+1)-f_{m}(i-1)}{f_{m}^{\max}-f_{m}^{\min}+\epsilon },\;m=1,\ldots ,M$$

where $f_{m}^{max}$ and $f_{m}^{min}$ denote the maximum and minimum values of the m-th objective within the considered front, and ϵ is a small positive constant introduced to avoid division by zero. Based on the resulting Pareto ranks and CD values, the updated population $P^{t+1}$ is formed by filling fronts in ascending order of rank. If the last selected front exceeds the remaining capacity, its solutions are sorted in descending order of CD, and only the top individuals are retained so that $|P^{t+1}|=N_{pop}$. In this way, Pareto rank acts as the primary selection criterion, while CD serves as a secondary discriminator among solutions with the same rank, thereby improving convergence toward the PF while preserving distribution and coverage along the front.

#### 3.2.3. Adaptive Student-*t* Heavy-Tailed Flight Strategy with Archive Feedback Mechanism

In the proposed search mechanism, the leader whales no longer use the random search operation in Equation (17). Instead, their positions are updated through archive guidance and an adaptive Student-*t* heavy-tailed flight [39].

To evaluate the effectiveness of the archive-guided search, the archive success rate $\rho_{t}$ is defined as the proportion of offspring that survive in the updated archive:

(23) $$\rho_{t}=\frac{\left|Q^{t}\cap A^{t+1}\right|}{\left|Q^{t}\right|}$$

To reduce fluctuations in the archive success rate, an exponential moving average parameter ${\bar{\rho }}_{t+1}$ is defined as follows:

(24) $${\bar{\rho }}_{t+1}=\left(1-\lambda \right){\bar{\rho }}_{t}+\lambda \rho_{t}$$

where λ∈[0,1] is the memory coefficient and the initial value is ${\bar{\rho }}_{0}=0.5$.

The normalized adaptive iteration parameters are defined as follows:

(25) $$\tau_{t}=\frac{t}{max\left(T-1,1\right)}$$

where t and T denote the current generation and maximum number of generations, respectively. For the i-th leader, an adaptive search state factor $\eta_{i}^{t}$ is constructed by combining the iteration progress, archive success-rate memory, and normalized crowding distance of its archive guide:

(26) $$\eta_{i}^{t}=clip\left[\tau_{t}+\left(1-\tau_{t}\right)\left(\omega {\bar{\rho }}_{t}+\left(1-\omega \right)\hat{d}\left(G_{i}^{t}\right)\right),0,1\right]$$

where ω∈[0,1] controls the relative contribution of archive success. $\hat{d}\left(G_{i}^{t}\right)$ is the normalized crowding distance, $G_{i}^{t}$ represents the *i-th* archive guide solution, and the Clip operator restricts the values of the tensor to the interval (0, 1).

Because the archive-dependent feedback term in Equation (26) is multiplied by $1-\tau_{t}$, its contribution is relatively strong during the early and intermediate search stages but gradually decreases as $\tau_{t}$ approaches one. This gradual attenuation allows archive-success memory and crowding information to guide exploration during the early and intermediate stages, while limiting their influence during the later stage to avoid excessive reliance on historical archive information during local refinement.

The degrees of freedom and flight scale of the Student-t distribution are then determined by

(27) $$\nu_{i}^{t}=1+\left(\nu_{\max}-1\right){\left(\eta_{i}^{t}\right)}^{2}$$

(28) $$\alpha_{i}^{t}=\alpha_{\min}+\left(\alpha_{\max}-\alpha_{\min}\right){\left(1-\eta_{i}^{t}\right)}^{2}$$

The position of the i*-th* leader whale is updated as follows:

(29) $$W_{i}^{new}=W_{i}+r_{i}⊙\left(G_{i}^{t}-W_{i}\right)+\alpha_{i}^{t}\xi_{i}^{t}u_{i}⊙\left(U-L\right)$$

where $r_{i}$ is a random vector uniformly distributed in ${\left[0,1\right]}^{D}$, and

(30) $$\xi_{i}^{t}\sim t_{\nu_{i}^{t}}\;\;u_{i}=\frac{v_{i}}{{\left\|v_{i}\right\|}_{2}}\;\;v_{i}\sim U{\left(-1,1\right)}^{D}$$

At the early search stage, a smaller $\eta_{i}^{t}$ produces fewer degrees of freedom and a larger flight scale, resulting in stronger heavy-tailed exploration. As the iteration progresses, the degrees of freedom increase and the flight scale decreases, allowing the search to shift gradually toward stable local refinement.

The follower whales retain the cooperative migration mechanism of the original WMA. However, a unique global-best solution cannot be identified directly in a multi-objective problem. Therefore, $W_{Best}$ in Equation (16) is replaced by an archive guide $G_{i}^{t}$ selected according to Equation (33). The position of the i*-th* follower is consequently updated as

(31) $$W_{i}^{new}=W_{Mean}+r_{1}⊙\left(W_{i-1}-W_{i}\right)+r_{2}⊙\left(G_{i}^{t}-W_{Mean}\right),\;\;\;i=N_{L}+1,\ldots ,N$$

where $W_{Mean}$ is calculated using Equation (15), and $r_{1}$ and $r_{2}$ are independent random vectors uniformly distributed in ${\left[0,1\right]}^{D}$. The first displacement term represents local learning from the preceding whale, whereas the second term directs the follower toward a representative non-dominated solution in the external archive.

The leader and follower updates are repeated for K internal migration steps, with boundary handling applied after each update. The resulting offspring are then evaluated, the external archive $A^{t}$ is updated, and the next population is determined through the NDS/CD-based selection mechanism described in Section 3.2.2.

#### 3.2.4. External Archive Assignment via Adaptive Probability Selection Factor

An external archive $A^{t}$ is maintained to preserve the non-dominated solutions obtained during the optimization process. The initial archive is constructed from the first non-dominated front of the initial population. After offspring population $Q^{t}$ is generated, the archive is updated according to

(32) $$A^{t+1}=T_{N_{A}}\left[ND\left(A^{t}\cup Q^{t}\right)\right]$$

where ND(⋅) extracts the non-dominated solutions, $N_{A}$ is the archive capacity, and $T_{N_{A}}\left(\cdot \right)$ denotes crowding-distance-based truncation. When the number of non-dominated solutions exceeds $N_{A}$, the solutions are arranged in descending order of crowding distance, and the first $N_{A}$ solutions are retained. In the present implementation, the archive capacity is equal to the population size.

Rather than employing an equal-weight selection strategy for archive guides, SMOWMA prioritizes solutions situated in sparse regions by assigning them higher selection probabilities. The probability factor for the *k*-th archive solution is calculated as follows:

(33) $$p_{k}=\frac{\delta_{p}+{\hat{d}}_{k}^{\gamma }}{\sum_{j=1}^{\left|A^{t}\right|}\left(\delta +{\hat{d}}_{j}^{\gamma }\right)}$$

where ${\hat{d}}_{k}\in \left[0,1\right]$ is the normalized crowding distance of the archive solution, γ controls the influence of crowding distance, and δ is a small positive constant that guarantees a non-zero selection probability. An archive guide parameter is sampled independently for each whale according to the probabilities in Equation (33).

Consequently, archived solutions located in less crowded regions are more likely to guide the population, which helps maintain the coverage of the Pareto front. In addition, the normalized crowding distance associated with the archive guide $G_{i}^{t}$ is also passed to the adaptive Student-*t* mechanism as a search-state feedback variable. The balance between convergence and diversity is achieved through the interaction of iteration progress, archive-entry success feedback, and archive crowding information. Iteration progress provides a global transition of the search behavior: fewer degrees of freedom and a larger flight scale produce heavy-tailed steps during the early stage, increasing the probability of exploring distant regions. As the search proceeds, the increasing degrees of freedom and decreasing flight scale reduce excessive perturbations and support local refinement. Unlike a purely iteration-dependent schedule, the archive-entry success memory reflects whether recently generated offspring contribute useful non-dominated solutions, allowing the flight behavior to respond to the actual search state. Meanwhile, crowding-weighted guide selection assigns higher probabilities to archive solutions located in sparse regions. This directs the population toward insufficiently covered portions of the Pareto front, while the preservation of non-dominated archive solutions maintains pressure toward high-quality objective regions. Therefore, the external archive provides both quality information for convergence and density information for diversity, whereas the adaptive Student-*t* distribution controls the movement range. Their interaction helps reduce premature concentration in limited front regions and avoids excessive disturbance during the later search stage.

### 3.3. The Pseudocode of SMOWMA

Algorithm 1 gives the complete workflow of SMOWMA. In the unified optimization process, the framework integrates maximin scrambled Sobol initialization, environmental selection based on non-dominated sorting and crowding distance, archive guide selection based on crowding distance, archive-guided follower-whale migration, adaptive Student-*t* heavy-tailed leader-whale updating driven by iteration progress and archive-entry success feedback, and the external Pareto archive maintenance mechanism.


**Algorithm 1.** Sobol-Driven Multi-Objective Whale Migration Algorithm (SMOWMA)
**Require:**
Population size N, dimension D, maximum generations $G_{max}$, inner iterations K, boundaries LB, UB, objective function f, and parameters $c,\;\lambda ,\;\omega ,\;\gamma ,\;\varepsilon_{p},\;\nu_{max},\;\alpha_{min},\;\alpha_{max}$.
**Ensure:**
Pareto optimal set represented by External Archive A.1

$N_{L}\leftarrow round\left(N/2\right),\;\Delta \leftarrow UB-LB$


// Part 1: Maximin Scrambled Sobol Initialization2Generate a scrambled Sobol candidate pool of size $2^{\left\lceil {log}_{2}\left(max\left(N,\lceil cN\rceil \right)\right)\right\rceil }$ in ${\left[0,1\right]}^{D}$
3Randomly select the first point and select the remaining N−1 points using the greedy maximin distance criterion4Map the selected points to [LB,UB] and construct population P
5Evaluate $f\left(X_{i}\right)$ for all $X_{i}\in P$
6

A←ArchiveUpdate(∅,P,N)

7Apply **FastNonDominatedSort** and crowding distance to P
8Sort P by ascending rank and descending crowding distance9

$P_{0}\leftarrow P,\;s_{A}\leftarrow 0.5,\;t\leftarrow 0$

10**while** $t<G_{max}$ **do**

// Part 2: CD-based Archive Leader Selection11
Calculate and normalize the crowding distance of each $A_{j}\in A$ to obtain ${\hat{d}}_{j}$
12


$p_{j}\leftarrow \frac{\varepsilon_{p}+{\hat{d}}_{j}^{\gamma }}{\sum_{A_{l}\in A}\left(\varepsilon_{p}+{\hat{d}}_{l}^{\gamma }\right)}$

13


$\tau_{t}\leftarrow t/max\left(G_{max}-1,1\right)$

14


$X^{\left(0\right)}\leftarrow \{X_{i}|X_{i}\in P_{t}\}$



// Part 3: Archive-guided WMA Migration15
**for** k←1 **to** K **do**16



$Mean\leftarrow \frac{1}{N_{L}}\sum_{i=1}^{N_{L}}X_{i}^{\left(k-1\right)}$

17

**for each individual** $X_{i}^{\left(k-1\right)}$ **do**18


Select archive guide $A_{i}$ according to $\left\{p_{j}\right\}$ and obtain its normalized distance ${\hat{d}}_{i}$
19


**if** $i\leq N_{L}$ *(Leader)* **then**20





$\eta_{i}\leftarrow clip\left(\tau_{t}+\left(1-\tau_{t}\right)\left[\omega s_{A}+\left(1-\omega \right){\hat{d}}_{i}\right],0,1\right)$

21





$\nu_{i}\leftarrow 1+\left(\nu_{max}-1\right)\eta_{i}^{2},\;\alpha_{i}\leftarrow \alpha_{min}+\left(\alpha_{max}-\alpha_{min}\right){\left(1-\eta_{i}\right)}^{2}$

22



Generate $r_{i}∼U{\left(0,1\right)}^{D}$, $u_{i}∼U{\left(-1,1\right)}^{D}$, and $z_{i}∼t_{\nu_{i}}$
23





$e_{i}\leftarrow u_{i}/∥u_{i}{∥}_{2}$

24





$Y_{i}\leftarrow X_{i}^{\left(k-1\right)}+r_{i}⊙\left(A_{i}-X_{i}^{\left(k-1\right)}\right)+\alpha_{i}z_{i}e_{i}⊙\Delta$

25



**else**
26



Generate $r_{1},r_{2}∼U{\left(0,1\right)}^{D}$
27





$Y_{i}\leftarrow Mean+r_{1}⊙\left(X_{i-1}^{\left(k-1\right)}-X_{i}^{\left(k-1\right)}\right)+r_{2}⊙\left(A_{i}-Mean\right)$

28



**end**
29




$X_{i}^{\left(k\right)}\leftarrow clip\left(Y_{i},LB,UB\right)$

30


**end**
31

**end**


// Part 4: Archive Update and Success Feedback32
Construct $Q_{t}\leftarrow \{X_{i}^{\left(K\right)}{\}}_{i=1}^{N}$ and evaluate all solutions in $Q_{t}$
33


$A\prime \leftarrow ArchiveUpdate\left(A,Q_{t},N\right)$

34


$r_{t}\leftarrow \frac{1}{N}\sum_{X_{i}\in Q_{t}}I\left(X_{i}\in A\prime \right)$

35


$s_{A}\leftarrow \left(1-\lambda \right)s_{A}+\lambda r_{t},\;A\leftarrow A\prime$



// Part 5: Environmental Selection36


$R_{t}\leftarrow P_{t}\cup Q_{t}$

37


$\{F_{1},F_{2},\ldots \}\leftarrow FastNonDominatedSort\left(R_{t}\right)$

38


$P_{t+1}\leftarrow \emptyset ,\;j\leftarrow 1$

39
**while** $\left|P_{t+1}\right|+\left|F_{j}\right|\leq N$ **do**40

Calculate crowding distance for $F_{j}$
41



$P_{t+1}\leftarrow P_{t+1}\cup F_{j},\;j\leftarrow j+1$

42

**end**
43
**if** $\left|P_{t+1}\right|<N$ **then**44

Sort $F_{j}$ by descending crowding distance and add the first $N-\left|P_{t+1}\right|$ solutions to $P_{t+1}$
45

**end**
46
Sort $P_{t+1}$ by ascending rank and descending crowding distance47


t←t+1

48
**end**
49**return** External Archive A



Algorithm 2 presents the archive-guided migration strategy embedded in Algorithm 1. In this strategy, the guide solution is selected from the external archive, and individuals located in relatively sparse archive areas are given a higher selection priority to improve the coverage of the Pareto frontier. Subsequently, the population evolved through the leader whale–follower whale update mechanism: the leader whale executed the archive-guided adaptive Student-*t* heavy-tailed flight adjusted by iterative progress, archive success feedback and the crowding distance of the guide solution, while follower whales moved under the joint action of the average position of the leader whales, the preceding whale and the selected archive guide.


**Algorithm 2.** Archive-Guided Migration Strategy of SMOWMA
**Require:**
Population P sorted by Pareto rank and Crowding Distance, External Archive A, inner migration iterations K, current generation t, maximum generations $G_{max}$, archive-success state $s_{A}$, boundaries LB, UB, objective function f, and parameters $\omega ,\;\gamma ,\;\varepsilon_{p},\;\nu_{max},\;\alpha_{min},\;\alpha_{max}$.
**Ensure:**
Updated offspring population Q.1

N←|P|

2

$N_{L}\leftarrow round\left(N/2\right)$

3

Δ←UB−LB


// CD-based archive leader distribution4Calculate Crowding Distance $d_{j}$ for each solution $A_{j}\in A$
5Normalize $d_{j}$ to obtain ${\hat{d}}_{j}$
6**for each archive solution** $A_{j}\in A$ **do**7


$p_{j}\leftarrow \frac{\varepsilon_{p}+{\hat{d}}_{j}^{\;\gamma }}{\sum_{A_{l}\in A}\left(\varepsilon_{p}+{\hat{d}}_{l}^{\;\gamma }\right)}$

8
**end**
9

$\tau_{t}\leftarrow \frac{t}{max\left(G_{max}-1,1\right)}$

10

$X^{\left(0\right)}\leftarrow \{X_{i}|X_{i}\in P\}$

11**for** k←1 **to** K **do**12


$Mean\leftarrow \frac{1}{N_{L}}\sum_{i=1}^{N_{L}}X_{i}^{\left(k-1\right)}$

13
**for** i←1 **to** N **do**14

Select archive guide $A_{i}\in A$ according to probabilities $\left\{p_{j}\right\}$
15

Let ${\hat{d}}_{i}$ be the normalized Crowding Distance of $A_{i}$
16

**if** $i\leq N_{L}$ *(Leader)* **then**



// Adaptive Student-t heavy-tailed flight17




$\eta_{i}\leftarrow clip\left(\tau_{t}+\left(1-\tau_{t}\right)\left[\omega s_{A}+\left(1-\omega \right){\hat{d}}_{i}\right],0,1\right)$

18




$\nu_{i}\leftarrow 1+\left(\nu_{max}-1\right)\eta_{i}^{2}$

19




$\alpha_{i}\leftarrow \alpha_{min}+\left(\alpha_{max}-\alpha_{min}\right){\left(1-\eta_{i}\right)}^{2}$

20


Generate archive-guidance vector $r_{i}∼U{\left(0,1\right)}^{D}$
21


Generate random direction $u_{i}∼U{\left(-1,1\right)}^{D}$
22




$e_{i}\leftarrow u_{i}/∥u_{i}{∥}_{2}$

23


Generate Student-t random variable $z_{i}∼t_{\nu_{i}}$
24




$Y_{i}\leftarrow X_{i}^{\left(k-1\right)}+r_{i}⊙\left(A_{i}-X_{i}^{\left(k-1\right)}\right)+\alpha_{i}z_{i}e_{i}⊙\Delta$

25


**else**




// Follower migration26


Generate $r_{1},r_{2}∼U{\left(0,1\right)}^{D}$
27




$Y_{i}\leftarrow Mean+r_{1}⊙\left(X_{i-1}^{\left(k-1\right)}-X_{i}^{\left(k-1\right)}\right)+r_{2}⊙\left(A_{i}-Mean\right)$

28


**end**
29



$X_{i}^{\left(k\right)}\leftarrow clip\left(Y_{i},LB,UB\right)$

30

**end**
31
**end**
32Construct $Q\leftarrow \{X_{i}^{\left(K\right)}{\}}_{i=1}^{N}$
33Calculate objective values $f\left(X_{i}^{\left(K\right)}\right)$ for all $X_{i}^{\left(K\right)}\in Q$
34**return** Q


### 3.4. Limitations in SMOWMA Biological Behavior

The whale-migration operators in SMOWMA are inspired by the collective migration of humpback whales, but they are not a quantitative, parameter-calibrated model of real whale navigation. The leader–follower hierarchy, the gravity or magnetic-field guidance, and the near-straight-line trajectory are used as analogies that motivate the algebraic update rules. They are neither derived from nor validated against empirical humpback-movement data. Accordingly, the algorithm is described throughout this paper as being inspired by whale migration behavior, rather than as mimicking or replicating it. No claim of biological fidelity is made; the biological parallel is used only as a design heuristic for structuring the search operators.

To keep the metaphor/operator boundary transparent, the main points at which the abstraction departs from the biological referent are disclosed as follows. (i) SMOWMA uses a discrete-generation, fixed-size, synchronously updated population with no demographic turnover, whereas real whale pods have variable composition and individual memory across seasons. (ii) The algorithm ranks all individuals globally each iteration, a centralized information state that no whale possesses; this is a centralized operator rather than a local sensing mechanism. (iii) The gravity/magnetic-field and direction vectors in the original WMA are sampled from uniform random distributions; this is a stochastic-search device rather than a model of magnetoreception or gravitational orientation. (iv) The target (global optimum) is fixed within a run, whereas a real migration endpoint is itself dynamic and perceptually mediated. (v) The non-dominated archive corresponds structurally, not behaviorally, to the survival of diverse strategies under coupled environmental pressures.

The computational value of SMOWMA rests on the multi-objective machinery (non-dominated sorting, crowding distance, Pareto archive, and archive-guided migration) and its empirical performance on the ZDT, DTLZ, WFG, UF, and CF suites and on the welded-beam design problem, rather than on any biological correctness. The whale-migration framing is therefore presented as a search-space-structuring metaphor whose usefulness is established numerically, not biologically. The correspondence between the whale-migration metaphor and the associated computational operators is summarized in Table 1.

## 4. Experimental Results

### 4.1. Benchmark Settings

The performance of SMOWMA is examined here with four commonly used multi-objective metrics, namely GD, IGD, HV, and SP. Its results are compared with those of MOWOA, MOSFOA, MORBMO, MOWMA, SMS-EMOA, MOEAD, SPEA2, NSGA-II, and NSGA-III on five benchmark families: ZDT, DTLZ, WFG, UF, and CF.

The benchmark problems used in the experiments include the following:

ZDT: ZDT1-ZDT4 and ZDT6;
DTLZ: DTLZ1-DTLZ6;
WFG: WFG1-WFG9;
UF: UF1-UF7;
CF: CF1-CF10.

These benchmark families involve a wide range of front shapes and search conditions, including non-convex, discontinuous, multimodal, degenerate, constrained, and many-objective cases, as well as problems with strong interactions among decision variables. For consistency, all algorithms are run under the same experimental settings, with identical generation limits and evaluation budgets.

Table 2 summarizes the parameter settings adopted for all compared algorithms in the benchmark experiments. All algorithms were run with the same basic settings: population size 200, maximum generation 1000, archive size 200, and 30 independent runs for each problem. In terms of objective settings, DTLZ was mainly tested in the three-objective case, while the other benchmark families followed their corresponding experimental configurations. For SMOWMA, the algorithm-specific parameters were set as follows: κ = 4, λ = 0.2, ω = 0.5, ν_max_ = 30, α_min_ = 0.01, α_max_ = 0.20, γ = 1.0, δ = 10^−3^, and K = 5. The archive-entry success memory was initialized as $\bar{\rho }$_0_ = 0.5. With N = 200 and κ = 4, the power-of-two Sobol sampling rule produced a candidate pool of Nc = 1024. The number of leader whales and the archive capacity were set to N_L_ = round(N/2) = 100 and N_A_ = N = 200, respectively.

For each benchmark problem, the HV reference point was determined from the corresponding fixed reference front according to $r_{j}=1.1{max}_{y\in PF_{ref}}y_{j}$. The resulting reference point was fixed before the experiments and remained identical for all algorithms and independent runs on the same problem. The adopted reference points are summarized in Table 3.

The reference point for each problem was fixed before the experiments and was shared by all algorithms and independent runs. For each objective, the reference-point component was set to 1.1 times the corresponding maximum value of the fixed reference front. The different numerical reference points reflect the different objective scales and Pareto-front geometries of the benchmark problems.

### 4.2. Parameter Sensitivity and Ablation Analysis

Before the full benchmark comparison, the principal SMOWMA parameters and the contribution of each newly introduced mechanism were examined separately. The sensitivity analysis evaluates the number of internal migration steps K, the maximum degrees-of-freedom parameter ν_max_ of the adaptive Student-*t* flight, and the archive-success weighting coefficient ω. The ablation study then removes or replaces one mechanism at a time while retaining the remaining settings and the same experimental protocol.

#### 4.2.1. Parameter Sensitivity

Table 4 reports the sensitivity results using the mean HV rank, mean IGD rank, their combined rank, and a composite score. Lower rank values and higher composite scores indicate more favorable settings. The results show that K = 5 provides the highest composite score (0.835), while K = 9 obtains the best IGD rank and ties the best combined rank. For the adaptive Student-*t* mechanism, ν_max_ = 30 yields the best combined rank (1.7) and the highest composite score (0.788). The setting ω = 0.5 gives the best HV, IGD, and combined ranks within its group. Accordingly, K = 5, ν_max_ = 30, and ω = 0.5 were adopted in the subsequent experiments because they provide the most balanced performance across the evaluated indicators.

#### 4.2.2. Component Ablation Study

Seven variants were constructed to isolate the effects of maximin scrambled Sobol initialization (MSSI), adaptive Student-*t* flight (ASTF), and the archive-guided strategy (AGS). Each variant changes only the mechanism listed in Table 5. SMOWMA-RI and SMOWMA-Sobol assess the two stages of MSSI; SMOWMA-LF and SMOWMA-FixedT assess the distributional and adaptive parts of ASTF; and SMOWMA-Uniform, SMOWMA-NoRho, and SMOWMA-NoAG assess crowding-weighted guide selection, archive-success feedback, and their combined contribution, respectively.

Table 6 summarizes the HV-based ablation results over the 15 test problems. The full SMOWMA achieves the best mean HV rank of 1.000 and obtains the highest mean HV on all 15 problems. Compared with each ablation variant, the full algorithm records 15 wins, with no ties or losses. The reduced variants have mean ranks ranging from 4.367 to 6.100, and none achieves the best HV on any problem. These results indicate that removing or replacing any individual mechanism consistently degrades the HV performance under the adopted experimental settings. Specifically, the results of SMOWMA-RI and SMOWMA-Sobol support the contribution of MSSI; those of SMOWMA-LF and SMOWMA-FixedT support the effectiveness of the adaptive Student-*t* flight mechanism; and those of SMOWMA-Uniform, SMOWMA-NoRho, and SMOWMA-NoAG confirm the complementary contributions of archive-guided selection and archive-success feedback.

### 4.3. Quantitative Performance Analysis

The hypervolume (HV) metric is used to comprehensively evaluate the approximation quality of a non-dominated solution set. A larger HV value indicates that the obtained solution set covers a larger dominated region in the objective space, whereas poor convergence or insufficient Pareto-front coverage generally results in a lower HV value. Table 7 presents the HV results obtained by SMOWMA and the comparison algorithms on 37 benchmark problems.

As shown in Table 7, SMOWMA obtains the highest mean HV on all five ZDT problems, including ZDT6. The results for the WFG suite vary among the algorithms. SMOWMA performs best on WFG2, WFG4, WFG6, WFG8, and WFG9; MOSFOA obtains the highest values on WFG1 and WFG7; SMS-EMOA leads on WFG3; and MOWOA performs best on WFG5. Therefore, SMOWMA demonstrates strong competitiveness on several WFG problems, although its advantage does not extend across the entire suite.

For the UF suite, SMOWMA obtains the highest mean HV on UF2, UF3, UF5, and UF6, whereas MOSFOA performs best on UF1, UF4, and UF7. The CF results also vary considerably with the constraint structure. SMOWMA achieves the highest mean HV on CF1, CF2, CF3, CF4, CF6, and CF9; MOSFOA performs best on CF5 and CF7; NSGA-III obtains the highest value on CF8; and MOEAD leads on CF10. These results indicate that SMOWMA provides favorable overall approximation quality on several constrained problems, although its performance remains affected by the feasible-region structure and the specific constraint characteristics.

The DTLZ suite similarly exhibits problem-dependent performance. SMOWMA obtains the highest mean HV on DTLZ2 and DTLZ4; MOEAD leads on DTLZ1; NSGA-III performs best on DTLZ3; MOWOA obtains the highest value on DTLZ5; and MOSFOA leads on DTLZ6. Therefore, the HV results support the effectiveness of SMOWMA on the ZDT suite and on selected WFG, UF, CF, and DTLZ problems, but they do not indicate uniform superiority across all benchmark families.

**Figure 5:**
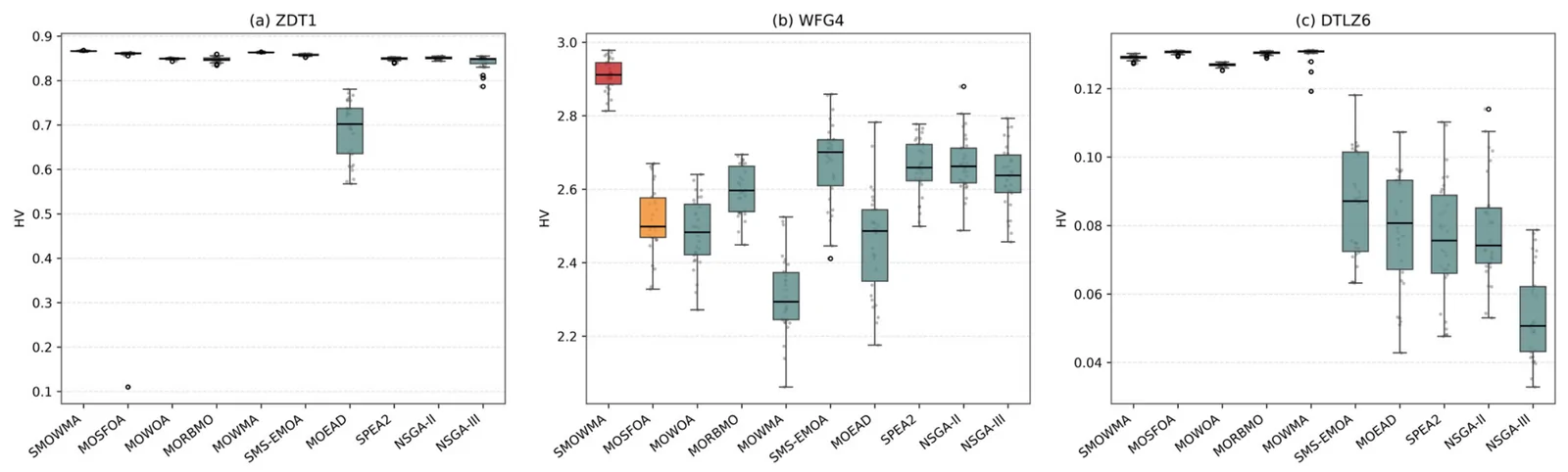
HV distributions across independent runs on (**a**) ZDT1, (**b**) WFG4, and (**c**) DTLZ6.

Although the mean and standard deviation reported in Table 7 summarize the overall HV performance, they do not fully characterize the median, interquartile range, and outliers of the results. Figure 5 presents boxplots of the HV values obtained by SMOWMA, MOSFOA, MOWOA, MORBMO, MOWMA, SMS-EMOA, MOEAD, SPEA2, NSGA-II, and NSGA-III.

As shown in Figure 5a, SMOWMA achieves the highest median HV on ZDT1 and maintains a compact distribution. MOWMA obtains a comparable median with similarly limited dispersion. MOSFOA also shows a relatively high median and a narrow central distribution, although one extremely low outlier is observed. MOWOA, MORBMO, SMS-EMOA, SPEA2, and NSGA-II exhibit slightly lower but generally stable HV values. In contrast, MOEAD shows the widest distribution and a substantially lower median, while NSGA-III presents several low-value outliers. These results indicate that SMOWMA provides favorable approximation quality and run-to-run stability on ZDT1.

As shown in Figure 5b, SMOWMA achieves the highest median HV on WFG4 and maintains a relatively narrow interquartile range. SMS-EMOA, SPEA2, NSGA-II, and NSGA-III form the next competitive group but obtain lower median values. MOWMA records the lowest median HV, whereas MOEAD exhibits relatively large dispersion. Therefore, SMOWMA shows a clear advantage in both HV level and stability on WFG4.

As shown in Figure 5c, SMOWMA belongs to the top-performing group on DTLZ6 and exhibits a compact distribution. MOSFOA and MORBMO obtain slightly higher median values, while MOWMA achieves a comparable median but presents several lower outliers. MOWOA records a slightly lower median with limited dispersion. SMS-EMOA, MOEAD, SPEA2, and NSGA-II exhibit lower HV values and greater variability, whereas NSGA-III records the lowest median HV. Overall, the boxplots indicate that SMOWMA combines high median HV with relatively low run-to-run variability on the three representative problems, although its relative ranking remains problem-dependent.

The detailed GD and IGD results are provided in Table A1 and Table A2 in Appendix A, respectively. The two indicators are distinct because GD mainly reflects the distance from the obtained solutions to the reference front, whereas IGD is also affected by how well the reference front is covered.

For the ZDT suite, SMOWMA records the lowest GD on ZDT3 and ZDT4, while its IGD is the lowest on ZDT1–ZDT4. For the WFG suite, SMOWMA obtains the lowest GD on WFG1 and ranks second in both GD and IGD on WFG6; its IGD on WFG1 also ranks second. Although SMOWMA achieves the highest HV on WFG2, WFG4, WFG8, and WFG9, its GD and IGD are not the lowest on these problems. This difference indicates that its advantage mainly lies in objective-space coverage and boundary-solution preservation rather than uniformly superior distance-based convergence.

The results for the DTLZ series are mixed. SMOWMA obtains the lowest GD on DTLZ3 and DTLZ5 but does not achieve the lowest IGD on any of the six problems. SMS-EMOA obtains the lowest GD on DTLZ1 and DTLZ2, while MOEAD and MOWOA lead on DTLZ4 and DTLZ6, respectively. For IGD, NSGA-III performs best on DTLZ1–DTLZ4, SPEA2 leads on DTLZ5, and MOWMA obtains the lowest value on DTLZ6. Nevertheless, SMOWMA ranks second in both GD and IGD on DTLZ6. Therefore, SMOWMA shows favorable distance-based convergence on selected DTLZ problems, but its reference-front coverage remains less competitive across this suite.

The UF and CF series show more complex results. In the UF suite, SMOWMA simultaneously achieves the lowest GD and IGD on UF5. In the CF suite, SMOWMA obtains the lowest GD on CF2, CF4, CF6, and CF7 and the lowest IGD on CF3, CF5, and CF7. CF7 is therefore the only CF problem on which SMOWMA performs best according to both indicators. Overall, SMOWMA is competitive on most ZDT problems and on selected WFG, DTLZ, UF, and constrained CF problems, but its advantages remain problem- and indicator-dependent. The following statistical tests are therefore used to determine whether the observed differences are statistically significant.

Convergence indicators alone cannot determine whether the obtained solutions are uniformly distributed along the Pareto front. The SP values reported in Table A3 in Appendix A evaluate this aspect from another perspective, with a lower SP value indicating more uniform solution spacing. For the WFG suite, SMOWMA obtains the lowest SP values on WFG1, WFG4, WFG5, and WFG6. In contrast, NSGA-II performs best on WFG2, MOSFOA on WFG3, MOWMA on WFG7, and MOWOA on WFG8 and WFG9. These results show that SMOWMA maintains favorable solution spacing on several WFG problems, although its advantage does not extend across the entire suite.

Outside the WFG suite, SMOWMA obtains the lowest SP values on ZDT1 and ZDT3, whereas MOEAD leads on ZDT2 and ZDT6, and MOSFOA performs best on ZDT4. Favorable results are also observed in the UF and CF suites. Specifically, SMOWMA achieves the lowest SP values on UF2, UF4, and UF5, as well as on CF3, CF4, CF5, and CF8. These results indicate that the diversity-preservation mechanisms of SMOWMA can maintain relatively uniform solution distributions on several unconstrained and constrained problems, although their effectiveness remains problem-dependent.

The DTLZ suite further illustrates this problem dependence. SMOWMA obtains the lowest SP value only on DTLZ2. MOEAD performs best on DTLZ1, SPEA2 achieves the lowest SP values on DTLZ3–DTLZ5, and MOWMA leads on DTLZ6. Compared with its performance on selected WFG problems, SMOWMA shows a less consistent spacing advantage on the DTLZ suite, indicating that its distribution quality is sensitive to the geometry and mapping characteristics of the benchmark problem.

### 4.4. Statistical Significance Analysis

To examine whether the observed differences among the compared algorithms are statistically meaningful, the Friedman test was conducted across all benchmark problems for each performance metric. The corresponding average ranks and omnibus test results are reported in Table 8.

The ranking pattern is not identical across the four metrics. SMOWMA obtains the lowest average ranks for IGD, HV, and SP, whereas SMS-EMOA achieves the lowest average GD rank, followed by SMOWMA. Nevertheless, the Friedman test returns a *p*-value below 0.05 for every metric, rejecting the null hypothesis that all algorithms perform equivalently. Since the Friedman test only identifies an overall difference among the ten methods, pairwise Wilcoxon signed-rank tests were further conducted between SMOWMA and each competing algorithm. The Holm-adjusted results are presented in Table 9.

The pairwise results reveal clear differences among the four indicators. For IGD, SMOWMA performs significantly better than every competing algorithm except MOSFOA after Holm correction. For HV and SP, SMOWMA performs significantly better than all nine competing algorithms. The outcome for GD is more limited: significant advantages are found over MOWOA, MORBMO, MOWMA, and MOEAD, but not over the other five methods. No comparison indicates a significant disadvantage for SMOWMA. Taken together, the tests provide strong support for its HV and SP performance, broad support for its IGD performance, and more selective support for its GD performance, without suggesting uniform superiority under every metric.

### 4.5. Pareto-Front Quality Across Benchmark Suites

**Figure 6:**
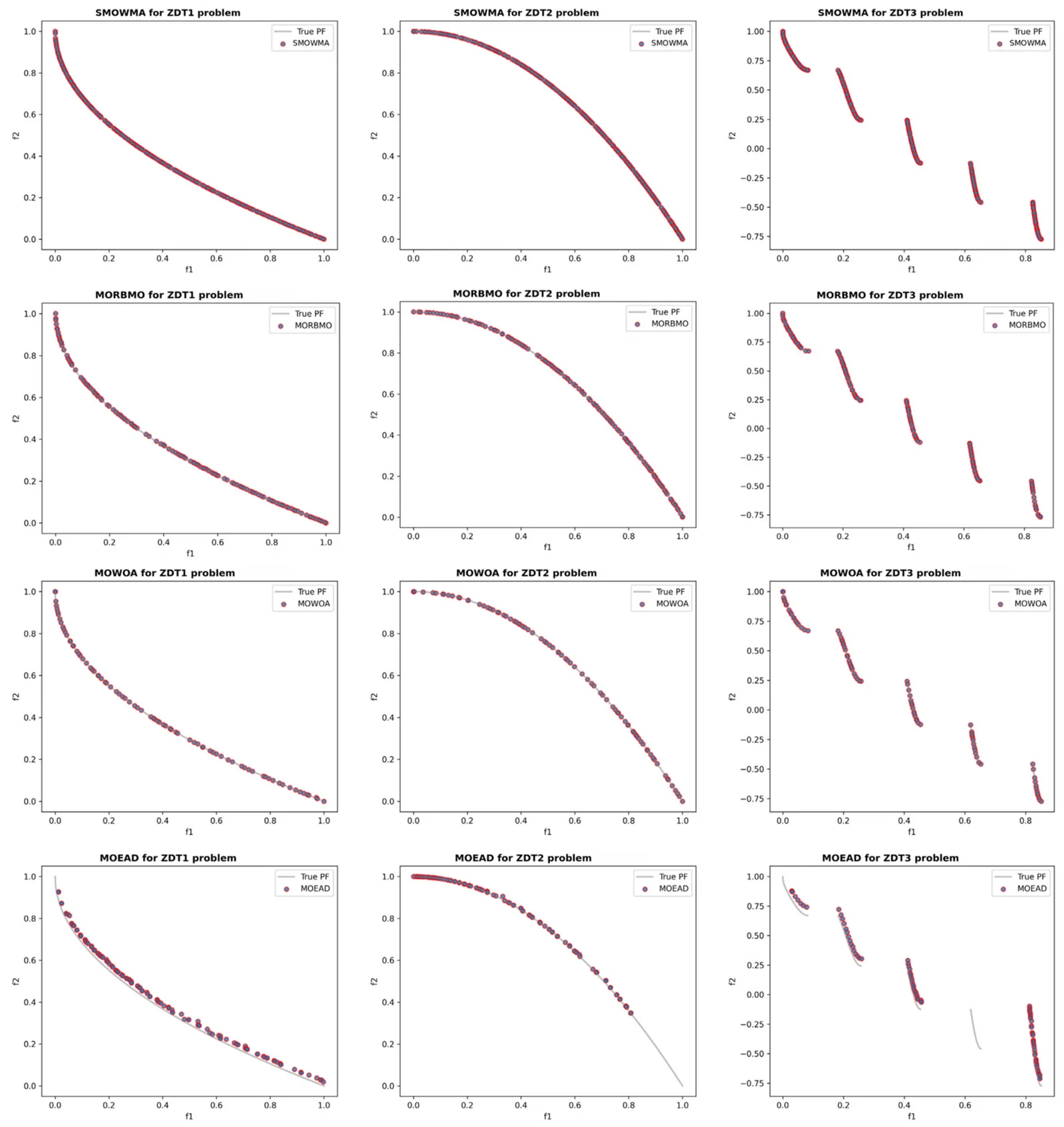
Pareto-front approximations obtained by different algorithms on representative ZDT problems.

Figure 6 shows the ZDT differences. On ZDT1 and ZDT2, the SMOWMA points almost stay on the true front, and the spread along the curve is also regular. ZDT3 is more revealing because the front is broken into separated segments. Here, the SMOWMA result still follows the main pieces of the front, while several competing methods leave empty intervals or place some solutions outside the target shape. The visual patterns are largely consistent with the numerical results, but they also reveal differences that are not always obvious from the metric tables alone.

This behavior may be partly related to the maximin scrambled Sobol initialization scheme. The scheme reduces the clustering of initial individuals and distributes candidate solutions more evenly in the decision space, thereby providing more diverse initial search directions and reducing the possibility of missing boundary regions or disconnected front segments. During the iterative process, crowding-distance-based archive guidance further promotes the preservation of sparse regions and boundary solutions. The adaptive Student-*t* heavy-tailed flight mechanism also helps the algorithm approach the true Pareto front through larger exploratory steps in the early stage and finer local adjustments in the later stage.

**Figure 7:**
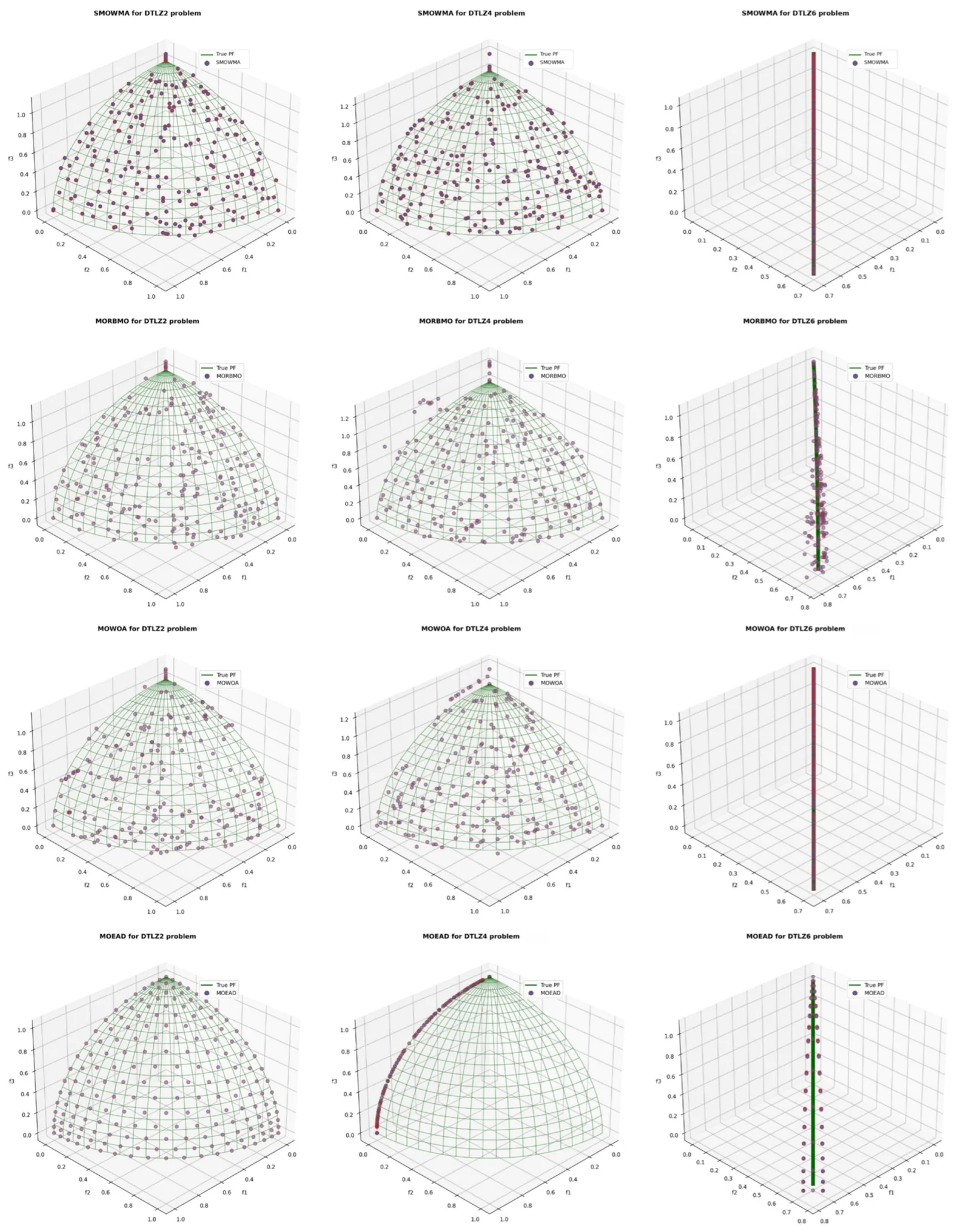
Pareto-front approximations obtained by different algorithms on representative DTLZ problems.

Figure 7 shows the Pareto-front approximations obtained by different algorithms on DTLZ2, DTLZ4 and DTLZ6. On DTLZ2, the results of the four algorithms are generally similar, and the solutions obtained are close to the reference front. SMOWMA maintains a relatively uniform distribution on the curved surface, but MOEAD also fits this regular front well. On DTLZ4, the differences between algorithms are more obvious. SMOWMA covers most regions of the front, while the MOEAD solutions are mainly concentrated near one boundary. DTLZ6 has a degenerate line-shaped Pareto front, and the SMOWMA solutions remain relatively densely distributed along the reference front, with fewer points that obviously deviate from the front. MOWOA also fits the reference line well, while MORBMO has more points scattered outside the front, and the approximation set obtained by MOEAD is relatively sparse. Overall, SMOWMA maintains competitive convergence and front coverage on these three DTLZ problems.

The performance of SMOWMA on these problems is consistent with the intended role of the archive-guided adaptive Student-*t* heavy-tailed flight strategy. In the early search stage, fewer degrees of freedom and a larger flight scale generate heavy-tailed search steps, enhancing long-range exploration and reducing the risk of premature population concentration in local regions. As the iterations proceed, the degrees of freedom gradually increase and the flight scale decreases, allowing the leader whales to conduct more stable local refinement near the Pareto front. Meanwhile, crowding-distance-based external archive maintenance preserves non-dominated solutions from different front regions, while sparse-region archive-guided selection gives solutions in less crowded regions greater influence on population updates. Together, these mechanisms help alleviate excessive population concentration in local front regions.

**Figure 8:**
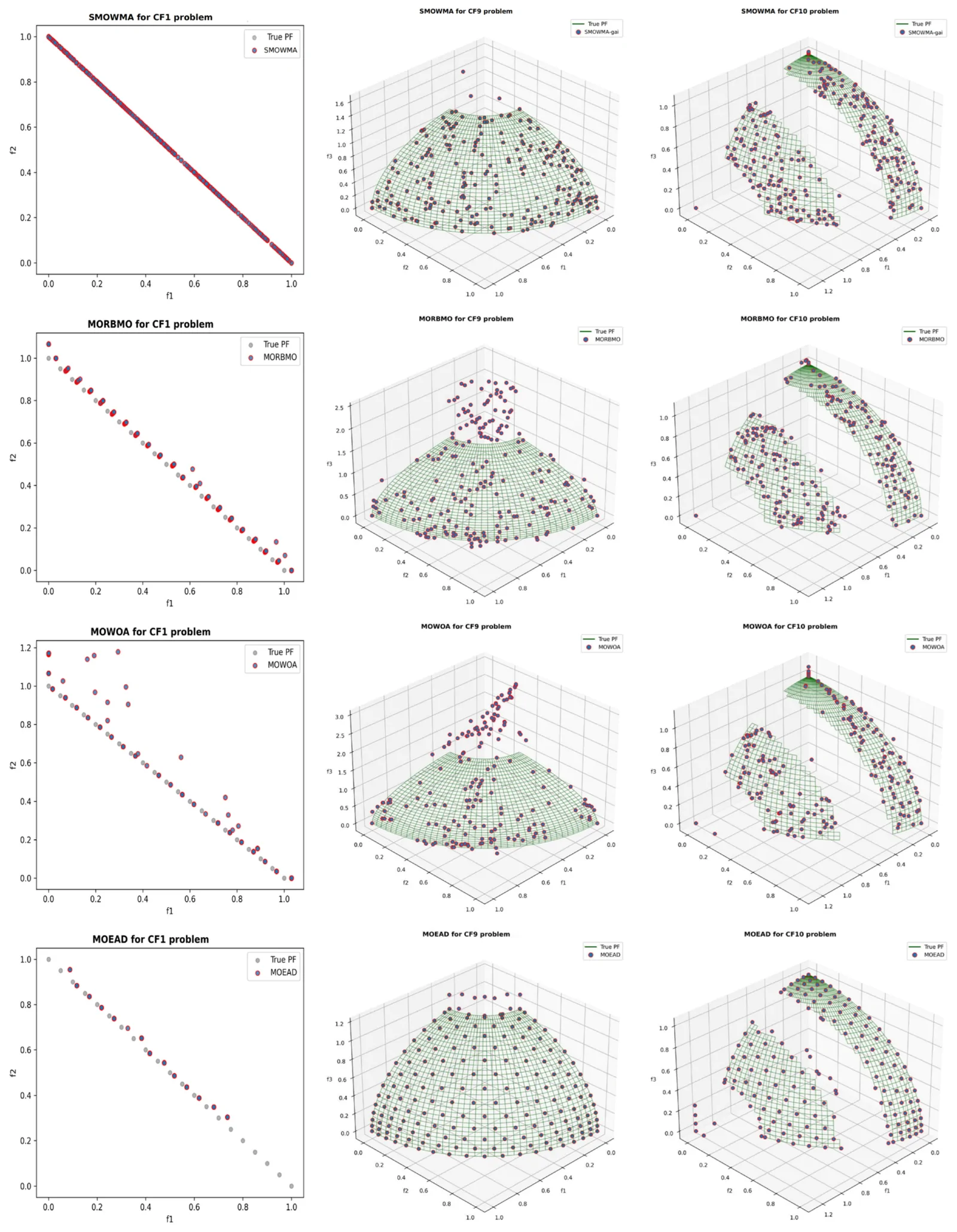
Pareto-front approximations obtained by different algorithms on representative CF problems.

Figure 8 shows the Pareto-front approximations obtained by different algorithms on representative CF problems. On CF1, the reference front has a continuous linear structure. The SMOWMA solutions are densely distributed along the reference front, with fewer points obviously deviating from it. MORBMO and MOEAD can also approach the reference front, but their solution distributions are relatively sparse; MOWOA has more points that deviate from the reference line. On CF9, SMOWMA maintains relatively complete and uniform coverage of the curved reference surface. In contrast, both MORBMO and MOWOA have some solutions that are obviously outside the reference surface, among which the deviation of MOWOA is more prominent. The solution distribution of MOEAD is more regular, but its overall density is lower than that of SMOWMA. CF10 has a more complex discontinuous Pareto-front structure. SMOWMA covers the two main front regions and forms a denser solution set over the curved surfaces. MORBMO also recovers the main structure of the front, but local deviations remain in some regions. The MOWOA solutions are relatively scattered and contain more points that deviate from the front; the distribution of MOEAD is more regular, but its coverage is relatively sparse in some regions. Overall, these visual results suggest that SMOWMA maintains competitive front proximity and coverage completeness on these three constrained problems.

These observations can be related to the combined effects of the external archive and the adaptive Student-*t* flight strategy. The CF series contains strongly constrained problems, among which CF9 and CF10 have disconnected or geometrically complex feasible Pareto fronts. On such problems, the external archive and its feedback mechanisms are expected to play a more direct role in the search process. The external archive preserves feasible non-dominated solutions obtained during the search, while crowding-distance-based guide selection increases the probability of selecting archive solutions from sparse regions as archive guides. This directs the population toward feasible front regions that have not been sufficiently explored. The archive-entry success rate is also fed back to the Student-*t* parameter adjustment process, allowing the search intensity to change according to the recent success of offspring entering the archive. The heavy-tailed steps generated by the Student-*t* distribution help the algorithm move between disconnected or insufficiently explored feasible regions, while the reduced flight scale in the later stage supports more accurate search near constraint boundaries. These mechanisms are consistent with the relatively low deviation and dense coverage observed for SMOWMA on CF9 and CF10. Nevertheless, this interpretation is based on the visual results, and the final comparison should rely on the quantitative metrics and statistical tests.

### 4.6. Convergence Behavior on Representative Benchmark Problems

**Figure 9:**
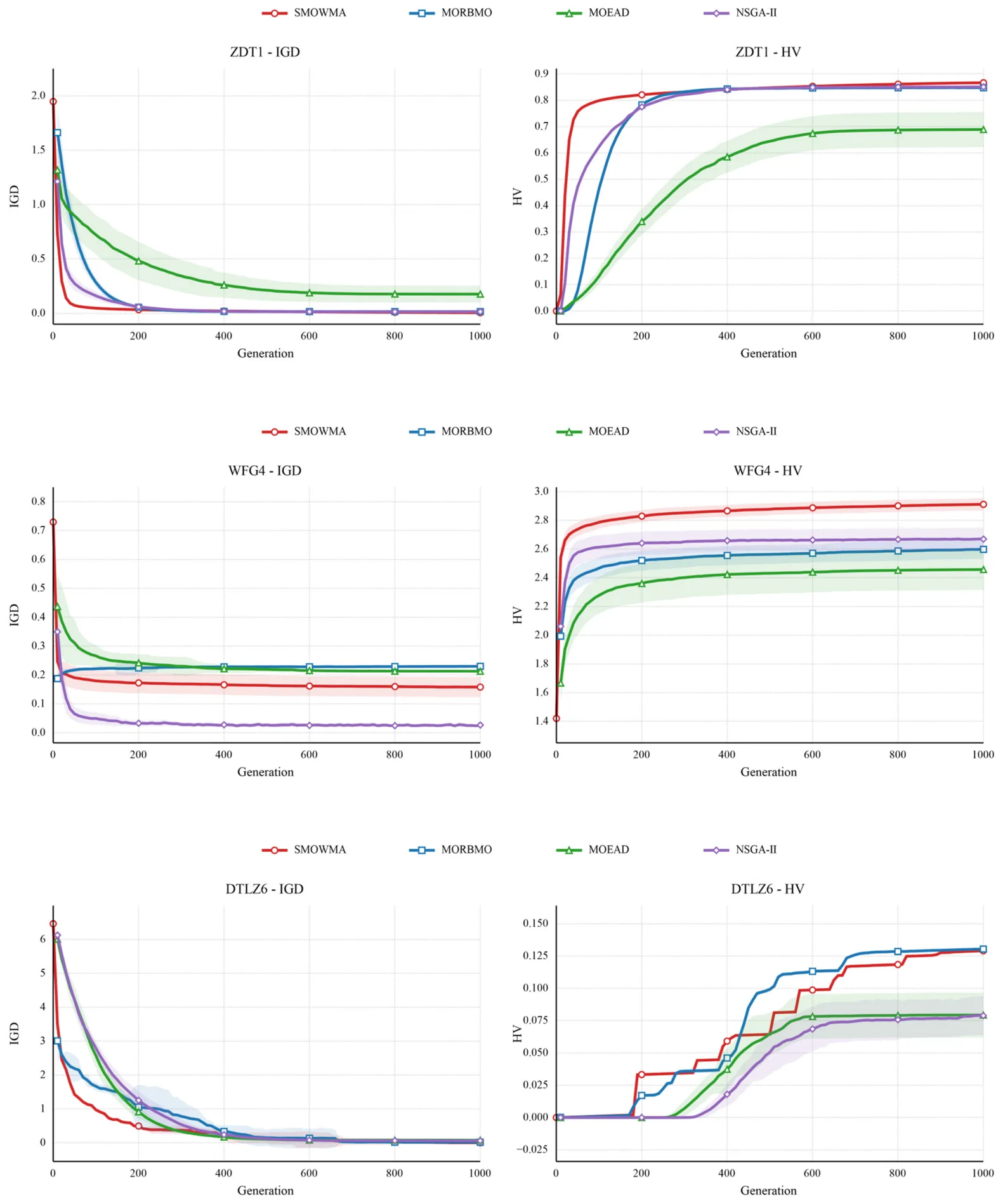
Evolution of benchmark function during the search process.

Final indicator values cannot reflect when an algorithm makes progress, nor can they indicate when its search process begins to slow down. Therefore, the IGD and HV values of four representative algorithms were recorded in Figure 9 during the runs on ZDT1, WFG4, and DTLZ6. ZDT1 is a two-objective problem with a relatively regular structure. WFG4 has multimodal characteristics, while DTLZ6 contains both biased variable mapping and a degenerate Pareto front. These three problems reflect the search process of the algorithms from different perspectives.

On ZDT1, the main differences between algorithms appear in the early stage of iteration. SMOWMA’s IGD curve declined rapidly and approached zero at about 200 generations. Its HV curve showed the opposite trend, rising rapidly in the same stage, followed by only a small increase. NSGA-II and MORBMO also gradually reached the low-IGD region, but they changed more slowly in the early stage. MOEAD required more generations to reduce IGD to a low level. At the end of the run, the four algorithms obtained similarly low IGD values, whereas SMOWMA maintained a clearly higher HV. Therefore, the curves on ZDT1 reflect the advantage of SMOWMA in both convergence speed and final HV.

WFG4 presents different results. SMOWMA improved quickly at the beginning of the search and maintained a higher HV than the other three algorithms throughout most of the iterations. Its final HV was followed by those of NSGA-II, MORBMO, and MOEAD. After the initial decline, the IGD of SMOWMA remained at a low level, with no obvious rebound in the later stage of the search. However, MOEAD and NSGA-II obtained slightly lower final IGD values. Therefore, SMOWMA achieves the strongest HV performance on WFG4, although it is not the leading method in terms of IGD.

The results on DTLZ6 show a mixed pattern. The IGD of SMOWMA declined rapidly during the early stage and stabilized at a lower level than those of the other three algorithms. MORBMO maintained the highest HV during most of the iterations, while the HV of SMOWMA increased more gradually and remained lower than that of MORBMO at 1000 generations. MOEAD and NSGA-II stabilized at relatively lower HV levels. Therefore, SMOWMA demonstrates favorable convergence according to IGD, although it does not achieve the best overall HV performance on this problem.

The three cases do not produce the same ordering. ZDT1 shows faster convergence and a higher final HV for SMOWMA, while WFG4 shows its advantage in HV but not in final IGD. DTLZ6 exposes weaker convergence behavior, particularly in terms of IGD. The curves therefore indicate problem-dependent search behavior rather than consistent superiority across all test functions.

### 4.7. Computational Complexity and Runtime Analysis

Let N denote the population size, D the decision-space dimension, M the number of objectives, G the maximum number of generations, K the number of inner migration steps, A the external-archive size (A≤N), and $C_{f}$ the cost of one objective-function evaluation. Because the candidate pool used by the scrambled Sobol procedure is a fixed multiple of N, maximin scrambled Sobol initialization (MSSI) has a one-time cost of $O\left(N^{2}D\right)$. At each generation, constructing the archive-guided distribution requires O(MAlogA+A), and the K archive-guided migration steps require O(KN(D+A)) under the direct categorical-sampling implementation. Offspring evaluation requires $O\left(NC_{f}\right)$. Archive updating is dominated by non-dominated sorting on at most A+N solutions, while archive-success feedback has a worst-case cost of O(NA). Environmental selection is dominated by non-dominated sorting of the combined parent-offspring population, while crowding-distance calculation requires O(MNlogN).

For readability, define the two per-generation cost groups as $Q_{1}=MAlogA+A+KN\left(D+A\right)+M{\left(A+N\right)}^{2},Q_{2}=NA+NC_{f}+MN^{2}+MNlogN.$ The total upper bound can then be written equivalently as $T_{SMOWMA}=O\left(N^{2}D+MN^{2}+NC_{f}+G\left(Q_{1}+Q_{2}\right)\right).$ For A≤N and fixed K, lower-order and dominated terms can be absorbed, giving $T_{SMOWMA}=O\left(N^{2}D+G\left[\left(M+K\right)N^{2}+KND+NC_{f}\right]\right).$

Thus, the dominant population-dependent order remains quadratic. This order is principally associated with the non-dominated sorting, archive management, and environmental selection operations already required by the multi-objective framework. MSSI introduces a one-time $O\left(N^{2}D\right)$ operation, whereas the adaptive Student-t flight (ASTF) mainly changes the constant factor of the position-update procedure. Therefore, the proposed mechanisms do not introduce a higher-order population-scaling term than the quadratic operations already required by the multi-objective framework. The execution-time comparison under equal function-evaluation budgets is presented in Table 10.

The runtime results do not support the claim that SMOWMA is universally the fastest method. NSGA-II, NSGA-III, SPEA2, and MOEAD require less execution time on these inexpensive analytical functions. Nevertheless, SMOWMA has the fifth-lowest four-problem mean runtime among the nine methods and is faster on average than MOWOA, MORBMO, and SMS-EMOA. More importantly, its mean runtime of 652.648 s is almost identical to the 653.320 s required by its direct MOWMA baseline, corresponding to a reduction of approximately 0.10%.

The problem-specific comparison with MOWMA is mixed. SMOWMA is 52.81% slower on ZDT1 and 5.72% slower on WFG2, but it is 1.14% faster on DTLZ2 and 2.19% faster on UF3. The comparatively large relative overhead on ZDT1 occurs because optimizer-internal operations are more visible when objective-function evaluations are inexpensive; the absolute difference on this problem is 30.49 s. Overall, the results indicate that MSSI, ASTF, and the archive-guided mechanisms do not materially increase the four-problem mean runtime relative to MOWMA under the tested settings. The proposed method therefore provides improved search behavior with a moderate computational trade-off, rather than a universal runtime advantage. For engineering problems with expensive objective evaluations, the $NC_{f}$ term may dominate, making optimizer-internal overhead relatively less influential.

The population and external archive require O((N+A)(D+M)) storage. If the non-dominated sorting implementation stores pairwise domination information, the worst-case auxiliary space complexity is $S_{SMOWMA}=O\left(\left(N+A\right)\left(D+M\right)+N^{2}\right)=O\left(N^{2}+N\left(D+M\right)\right),A\leq N.$

## 5. Constrained Engineering Design Applications

### 5.1. Unified NHV-Based Performance Evaluation

To provide a unified set-level comparison, all algorithms are evaluated using the normalized hypervolume (NHV), which simultaneously reflects the convergence and objective-space coverage of the obtained non-dominated approximation sets. Let $P_{a,r}$ denote the feasible non-dominated set obtained by algorithm a in the r-th independent run. Only solutions satisfying $CV\left(x\right)\leq 10^{-6}$ are retained. If no feasible solution is obtained in a run, its NHV value is assigned as zero.

For each welded-beam formulation, the approximation sets obtained by all algorithms over all independent runs are pooled to establish common normalization bounds:

(34) $$U=\overset{A}{\underset{a=1}{\cup }}\overset{Nr}{\underset{r=1}{\cup }}P_{a,r},\;\;\;\;z_{i}^{min}=min_{x\in U}f_{i}(x),\;\;\;\;z_{i}^{max}=max_{x\in U}f_{i}(x)$$

where A is the number of compared algorithms, $N_{r}$ is the number of independent runs, and ${\hat{P}}_{a,r}$ denotes the normalized approximation set corresponding to $P_{a,r}$. Each objective is normalized as

(35) $${\hat{f}}_{i}(x)=\frac{f_{i}(x)-z_{i}^{\min}}{z_{i}^{\max}-z_{i}^{\min}+\varepsilon }$$

where $\varepsilon =10^{-12}$ prevents division by zero. A fixed reference point q=(1.1,…,1.1) is adopted in the normalized objective space. The NHV value of algorithm a in run r is calculated as

(36) $$NHV_{a,r}=\frac{HV\left({\hat{P}}_{a,r},q\right)}{\prod_{i=1}^{Mn}q_{i}}=\frac{HV\left({\hat{P}}_{a,r},q\right)}{1.1^{Mn}}$$

where $M_{n}$ is the number of objectives and a larger NHV value indicates better overall approximation quality. Each algorithm is independently executed $N_{r}=30$ times under the same computational budget. The mean and standard deviation of NHV are reported as

(37) $$\begin{array}{l} {\bar{NHV}}_{a}=\frac{1}{R}\sum_{r=1}^{Nr}NHV_{a,r}\\ s_{a}=\sqrt{\frac{1}{R-1}\sum_{r=1}^{Nr}{\left(NHV_{a,r}-{\bar{NHV}}_{a}\right)}^{2}} \end{array}$$

Pairwise differences between SMOWMA and each compared algorithm are assessed using the Wilcoxon rank-sum test at a significance level of α=0.05, with Holm correction applied to multiple comparisons. The overall ranking is determined from the mean NHV rank across the two-, three-, and four-objective formulations. For graphical presentation, the representative approximation set of each algorithm is taken from the run whose NHV value is closest to its median NHV. These representative runs are used only for visualization, whereas the quantitative conclusions are based on the complete NHV statistics.

### 5.2. Formulation of Constrained Welded Beam Design Problem

Engineering optimization in practice is rarely straightforward. Nonlinear behavior, local optima, and restrictive constraints often make the search difficult, and the situation becomes more complicated when one design variable influences several objectives or constraints at the same time. As a result, an effective optimizer must search broadly without losing control of these coupled design effects. In this paper, the proposed SMOWMA is applied in the welded beam design problem.

The general form of a constrained multi-objective welded beam design problem is written as follows [40]:

(38) $$Minimize:F(x)={\left[f_{1}(x),f_{2}(x),f_{3}(x),f_{4}(x)\right]}^{T}s.t.\;{\bar{g}}_{j}(x)\leq 0,\;\;\;\;j=1,\ldots ,5.$$

where $x={\left[h,l,t,b\right]}^{T}$ is the continuous decision-variable vector, Ω denotes the admissible design space defined by the variable bounds and manufacturing limitations, and ${\bar{g}}_{j}\left(x\right)$ denotes the j-th physical or geometric constraint.

(39) $$\left\{\begin{array}{l} f_{1}(x)=1.10471h^{2}l+0.04811tb(14.0+l)\\ f_{2}(x)=\frac{4PL^{3}}{Et^{3}b}\\ f_{3}(x)=\sqrt{{\left(\tau^{\prime }\right)}^{2}+2\tau^{\prime }\tau^{\prime \prime }\frac{l}{2R}+{\left(\tau^{\prime \prime }\right)}^{2}}\\ f_{4}(x)=\frac{6PL}{bt^{2}} \end{array}\right.$$

where $f_{1}$(CU) is the fabrication cost, $f_{2}$(in) is the end deflection, and $f_{3}$(psi) and $f_{4}$(psi) are the shear stress and bending stress, respectively. Furthermore, h(in) denotes the weld thickness, l(in) is weld length, and t(in) and L(in) are beam thickness and length, respectively. P(lbf) is the applied load, and E(psi) is Young’s modulus.

To maintain physical admissibility under the assumptions of the benchmark, the same allowable stress, deflection, geometric, and buckling constraints are retained in all formulations, even when deflection or stress is also treated as an optimization objective. The subsequent constraint functions ${\bar{g}}_{j}\left(x\right)$ are evaluated using the following mechanical relationships:

(40) $$\left\{\begin{array}{l} \tau^{\prime }=\frac{P}{\sqrt{2}hl}\\ \tau^{\prime \prime }=\frac{MR}{J_{w}}\\ M=P\left(L+\frac{l}{2}\right)\\ R=\sqrt{\frac{l^{2}}{4}+{\left(\frac{h+t}{2}\right)}^{2}}\\ J_{w}=2\sqrt{2}hl\left[\frac{l^{2}}{12}+{\left(\frac{h+t}{2}\right)}^{2}\right]\\ P_{c}(x)=\frac{4.013E}{L^{2}}\sqrt{\frac{t^{2}b^{6}}{36}}\left[1-\frac{t}{2L}\sqrt{\frac{E}{4G}}\right] \end{array}\right.$$

where $\tau^{\prime }$(psi) is the primary shear stress acting on the weld throat, $\tau^{\prime \prime }$(psi) is the secondary shear stress induced by the load moment, M(lbf·in) is the applied-load moment, R(in) is the distance from the weld-group centroid to the critical point, $J_{w}$(in^4^) is the polar moment of inertia of the weld group, and $P_{c}$(lbf) is the critical buckling load of the beam.

In the benchmark formulation, the applied load and beam length are P=6000 lbf and L=14 in. The elastic modulus and shear modulus are $E=30\times 10^{6}$ and $G=12\times 10^{6}\;psi$. The allowable weld shear stress, allowable beam bending stress, and maximum end deflection are $\tau_{max}=13,600$ psi, $\sigma_{max}=30,000$ psi, and $\delta_{max}=0.25$ in, respectively. The fabrication-cost objective $f_{1}$ is reported in the conventional cost unit (CU) of the benchmark and should not be interpreted as the current industrial price.

Based on the above mechanical relationships and prescribed allowable limits, the five normalized constraint functions imposed in all objective formulations are expressed as follows:

(41) $$\left\{\begin{array}{l} {\bar{g}}_{1}(x)=\frac{\tau (x)}{\tau_{\max}}-1\leq 0\\ {\bar{g}}_{2}(x)=\frac{\sigma (x)}{\sigma_{\max}}-1\leq 0\\ {\bar{g}}_{3}(x)=\frac{\delta (x)}{\delta_{\max}}-1\leq 0\\ {\bar{g}}_{4}(x)=\frac{h}{b}-1\leq 0\\ {\bar{g}}_{5}(x)=\frac{P}{P_{c}(x)}-1\leq 0 \end{array}\right.$$

These parameter values and allowable limits are prescribed benchmark conventions rather than values derived from a specific contemporary welding design code.

For the purpose of feasibility evaluation during evolution, the constraint violation (CV) of a candidate solution is defined as follows:

(42) $$CV(x)=\sum_{j=1}^{5}\max\left(0,{\bar{g}}_{j}(x)\right).$$

The welded-beam design (WBD) problem is employed as a standard constrained engineering benchmark to examine the influence of increasing objective dimensionality and the constraint-handling performance of the compared algorithms. The problem is evaluated entirely through its established analytical formulation. Therefore, the results provide numerical benchmark evidence rather than experimental, industrial, or high-fidelity validation of a real welded structure. Three formulations of the same benchmark, involving two, three, and four objectives, are considered while maintaining an identical feasible design region. This controlled setting isolates the effect of objective dimensionality without introducing differences between unrelated engineering problems.

#### 5.2.1. NHV Results and Statistical Comparison for Welded-Beam Design

Table 11 summarizes the NHV results obtained for the two-, three-, and four-objective welded-beam formulations. The values are reported as the mean and standard deviation over 30 independent runs. Since a larger NHV indicates better overall convergence and objective-space coverage, these normalized results provide a consistent basis for evaluating the algorithms within each formulation and examining their performance as the number of objectives increases.

The two-objective case provides only limited separation among the three leading algorithms. SMOWMA achieves the highest mean NHV, followed closely by MORBMO and NSGA-II. It also has the smallest standard deviation, indicating relatively stable performance across the 30 independent runs. The statistical results in Table 12 show that the differences between SMOWMA and these two algorithms are not significant. In contrast, its advantages over MOEAD and MOWOA are statistically significant.

A different pattern is observed for the three-objective formulation. MORBMO records the highest mean NHV and the smallest standard deviation, while SMOWMA ranks third. The difference between MORBMO and SMOWMA remains significant after Holm correction. No significant difference is found between SMOWMA and NSGA-II, although NSGA-II achieves a slightly higher mean NHV. SMOWMA nevertheless significantly outperforms MOEAD and MOWOA in this formulation.

The four-objective case provides the most favorable result for SMOWMA. It achieves both the highest mean NHV and the smallest standard deviation among the compared methods. The pairwise differences between SMOWMA and all four competing algorithms are statistically significant after Holm correction. Although its numerical advantages over NSGA-II and MORBMO are relatively small, they remain significant at the adopted significance level. These results demonstrate the strong approximation quality and stability of SMOWMA in the more complex four-objective welded-beam formulation.

Table 12 reports the Holm-adjusted p-values obtained from the pairwise Wilcoxon rank-sum tests based on 30 independent runs. The statistical results do not indicate that SMOWMA is superior in every formulation. In the two-objective case, SMOWMA achieves the highest mean NHV but does not differ significantly from NSGA-II or MORBMO. In the three-objective case, it performs significantly worse than MORBMO but remains statistically comparable with NSGA-II. In the four-objective case, SMOWMA significantly outperforms all four competing algorithms. These results demonstrate that its relative performance varies with the number of objectives and should not be assessed solely from the mean NHV values.

Considering the descriptive ranks across the three formulations, SMOWMA obtains the lowest mean rank of 1.667, followed by MORBMO and NSGA-II with mean ranks of 2.000 and 2.333, respectively. SMOWMA ranks first in the two- and four-objective cases but third in the three-objective case. These mean ranks are used only as a compact descriptive summary of the three objective settings rather than as a separate statistical significance test.

#### 5.2.2. Pareto-Front Analysis of the Multi-Objective Welded-Beam Design Problem

In the two-objective application, manufacturing cost $f_{1}$ and end deflection $f_{2}$ are set as two optimization targets. Therefore, the task aims to reduce costs while avoiding the loss of structural stiffness exceeding the specified deflection limit. In this case, shear stress and bending stress are not used as optimization targets but are limited by corresponding allowable stress constraints. In addition, the maximum end deflection constraints, geometric compatibility constraints and buckling constraints are also retained; therefore, all five constraints defined in the formula are applicable to this model defined in Equation (42).

**Figure 10:**
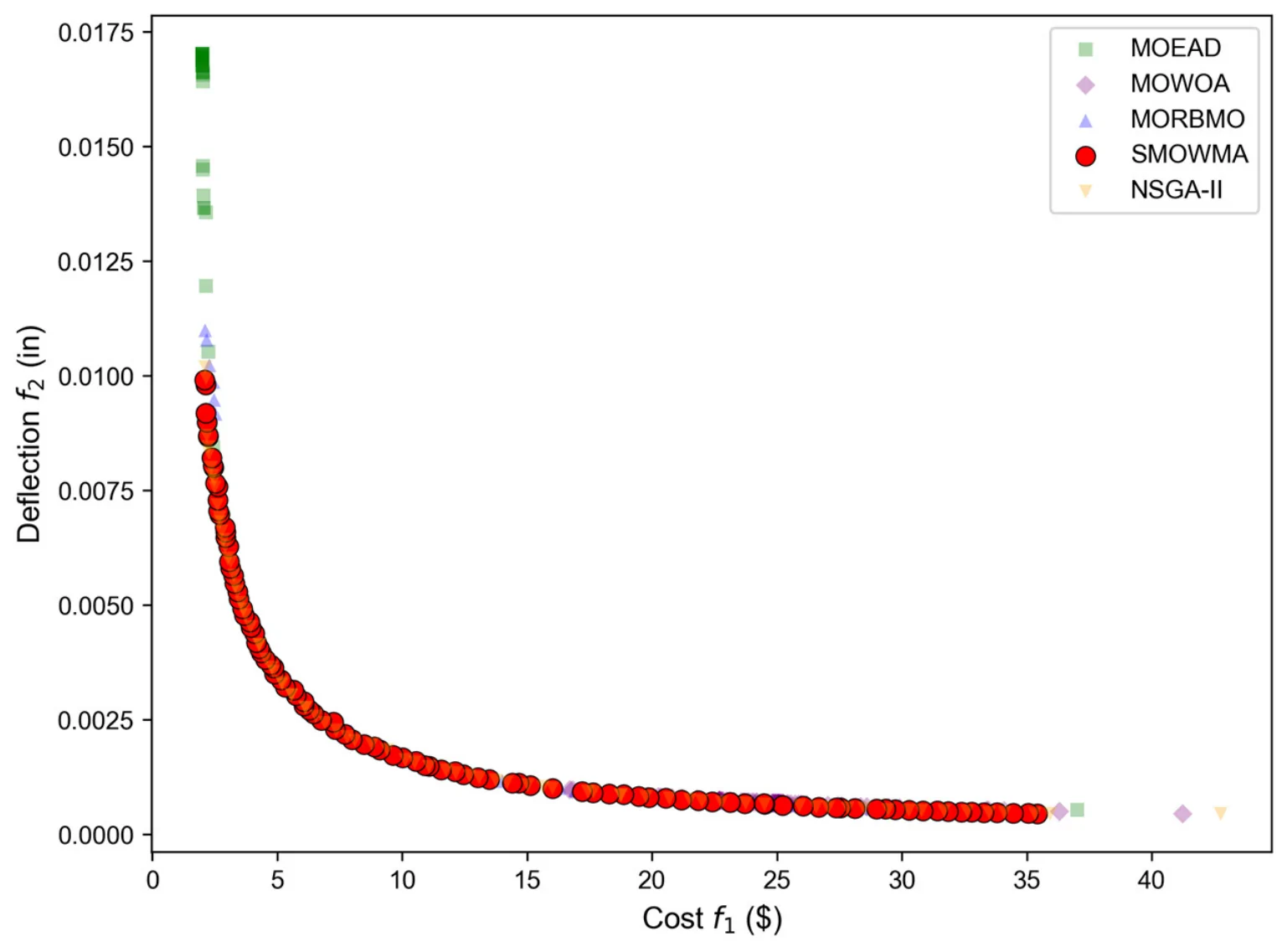
Pareto front for the 2-objective problem.

Figure 10 displays the representative non-dominated sets obtained by the compared algorithms for the bi-objective welded-beam design problem. The front generated by SMOWMA is continuous, and its points remain fairly well distributed over the trade-off range. From the figure, the SMOWMA solutions stay closer to the observed trade-off boundary than those of MOEAD and MOWOA, which is consistent with the NHV results in Table 11. The MOWOA points are visibly more scattered and remain farther from this boundary. NSGA-II and MORBMO reach a similar level of convergence over part of the front, while the SMOWMA front remains relatively smooth over a wider portion of the curve.

The complexity of the welded beam design problem is increased by incorporating shear stress $f_{3}$ as an optimization objective, while maintaining its allowable-stress constraint. Bending stress $f_{4}$ remains subject to an allowable stress constraint to ensure structural stiffness under load. This three-objective optimization is used to validate the SMOWMA, aiming to identify Pareto-optimal solutions that balance structural safety (via shear stress) with economic considerations (fabrication cost) and stiffness (represented by end deflection).

**Figure 11:**
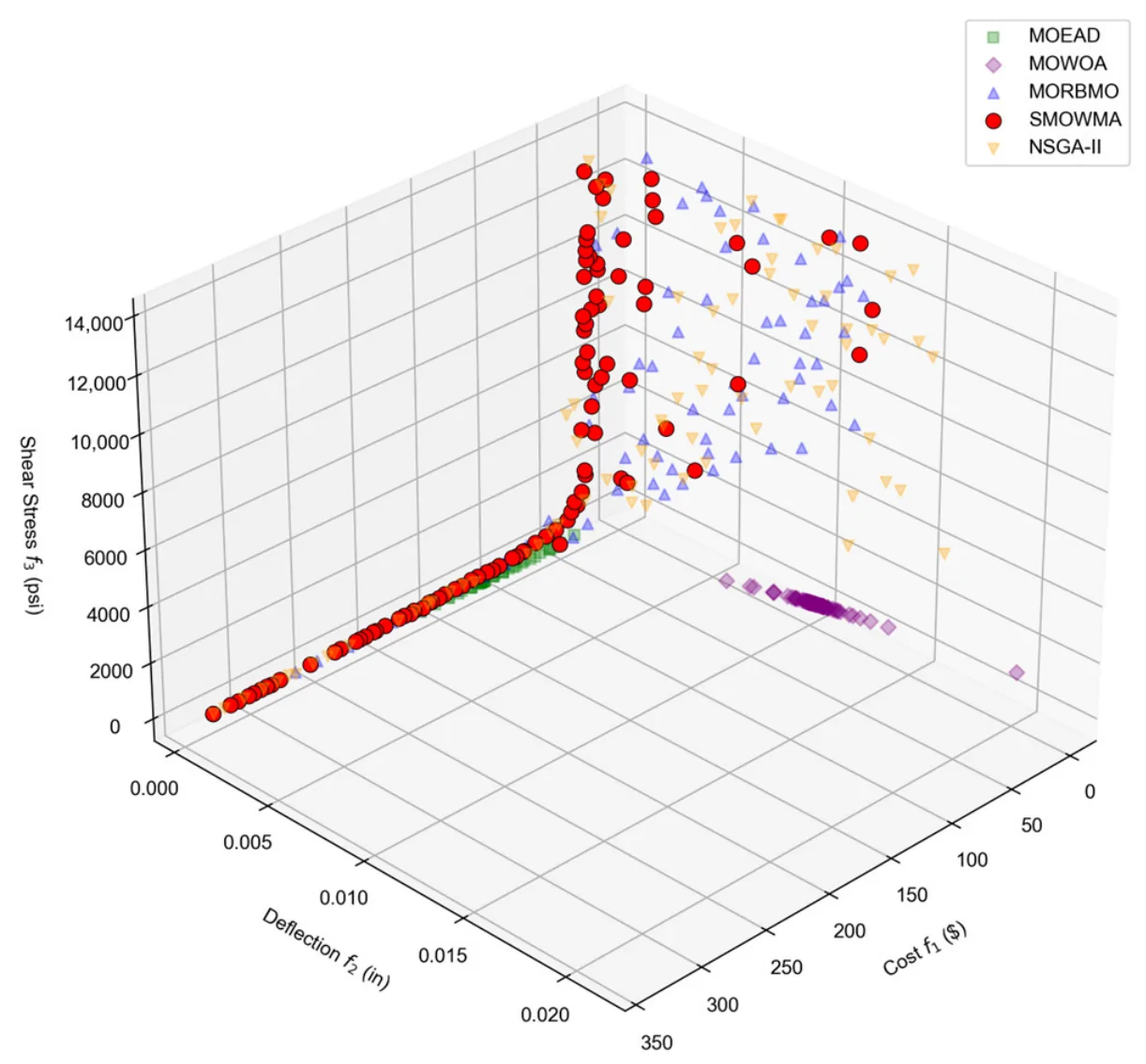
Pareto front for the three-objective welded-beam design problem.

Figure 11 presents the comparative Pareto fronts for the three-objective welded-beam design problem. Increasing shear stress complicates the trade-off surface between fabrication cost, end deflection, and structural stress. Among the algorithms, MOEAD shows strong convergence in low-deflection or stress regions but poor diversity in the low-cost domain. Conversely, MOWOA suffers from scattered solutions and significant deviation from the Pareto front. MORBMO achieves a wide distribution and a competitive front across most of the search space. SMOWMA provides a balanced set of solutions that cover diverse trade-off regions without bias toward a single objective, performing similarly to NSGA-II. Although MORBMO excels in specific regions, SMOWMA remains a robust and competitive solver for this three-objective problem.

**Figure 12:**
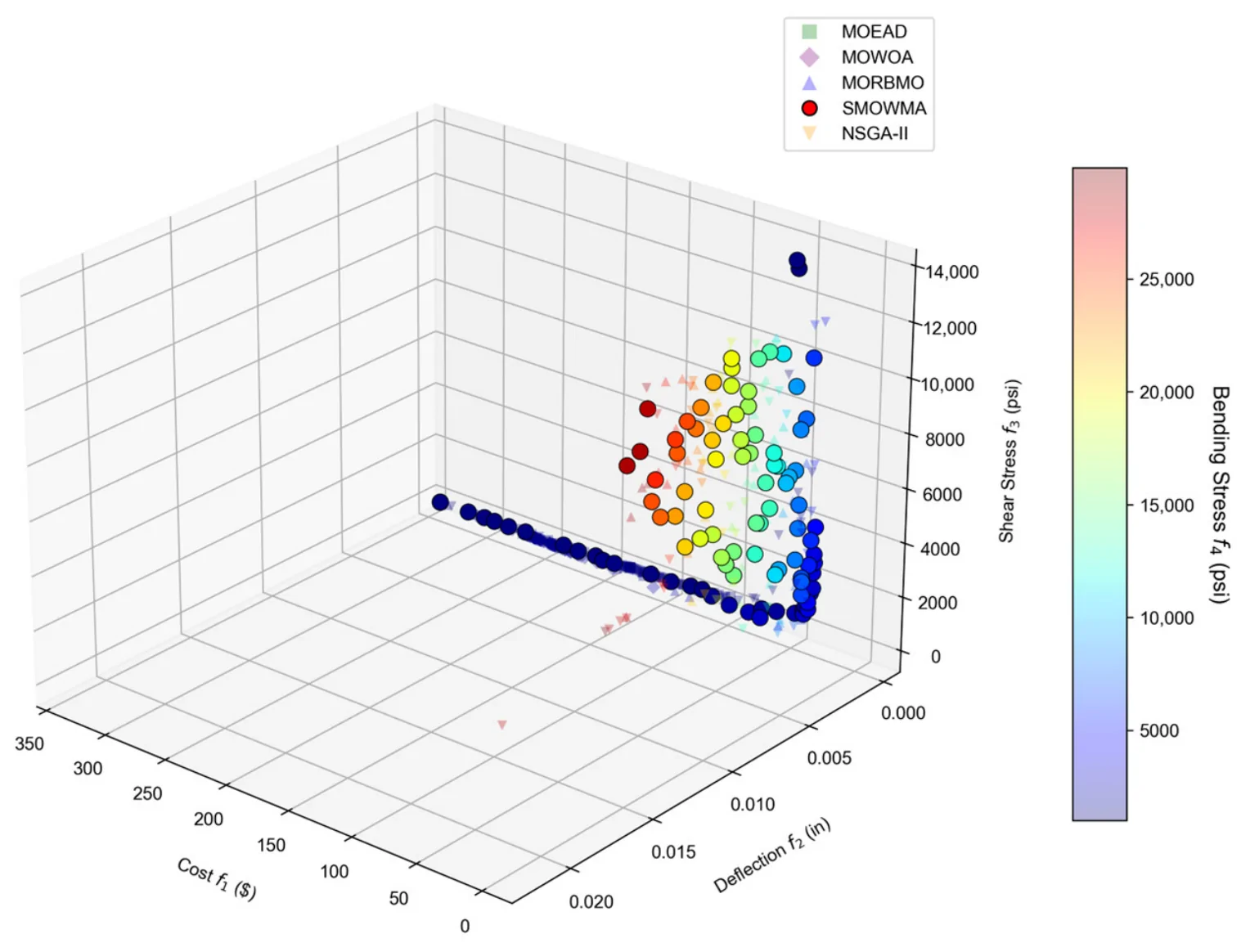
Pareto front for the 4-objective welded beam design problem.

By incorporating shear stress $f_{3}$ and bending stress $f_{4}$ as objectives alongside cost and deflection, the four-objective formulation creates a complex trade-off surface within the mechanically feasible design space (defined by constraints $g_{1}$-$g_{5}$). As shown in Figure 12, this complexity challenges the multi-objective algorithm’s ability to maintain both convergence and diversity. SMOWMA distinguishes itself by distributing Pareto-optimal solutions evenly across the objective space, avoiding the clustering seen in some competitors. In contrast, while NSGA-II and MORBMO reach similar trade-off regions, MOEAD and MOWOA struggle with coverage, with MOWOA showing excessive dispersion in stress-related metrics. These results confirm SMOWMA’s capability to navigate high-dimensional trade-offs effectively, providing a balanced set of Pareto-optimal solutions.

Overall, the NHV results, statistical comparisons, and Pareto-front distributions jointly characterize the performance of the algorithms on the constrained welded-beam design problem. However, these results are derived from different objective formulations of the same engineering benchmark. To further examine the applicability of SMOWMA to a more complex and higher-dimensional constrained engineering problem, an additional four-bar truss design problem and five-objective car side-impact design problem is considered. In addition to solution quality and statistical significance, the execution times of SMOWMA and the comparison algorithms are evaluated to provide a more complete assessment of computational performance.

### 5.3. Four-Bar Truss Design Problem

To further evaluate the performance of SMOWMA on structural engineering design problems, the four-bar truss design benchmark was considered [41]. The design vector is defined as x = (x_1_, x_2_, x_3_, x_4_), where x_1_–x_4_ represent the cross-sectional areas of the four truss members. The two conflicting objectives are the minimization of structural volume f_1_ and structural displacement f_2_, expressed as

(43) $$\left\{\begin{array}{ccc} \underset{x}{\min}F(x) & = & \left[f_{1}(x),f_{2}(x)\right]\\ f_{1}(x) & = & 200\left(2x_{1}+\sqrt{2}x_{2}+\sqrt{x_{3}}+x_{4}\right)\\ f_{2}(x) & = & 0.01\left(\frac{2}{x_{1}}+\frac{2\sqrt{2}}{x_{2}}-\frac{2\sqrt{2}}{x_{3}}+\frac{2}{x_{4}}\right) \end{array}\right.$$

The allowable ranges of the design variables are $1\leq x_{1},\;x_{4}\leq 3,\;1.4142\leq x_{2},\;x_{3}\leq 3$.

This formulation contains no additional inequality constraints beyond the variable bounds. Increasing the member cross-sectional areas generally reduces structural displacement but increases material volume, resulting in a distinct trade-off between structural efficiency and stiffness. SMOWMA was independently executed 30 times using a population size of 200, a maximum of 1000 generations, and an archive capacity of 200. The approximation fronts and normalized hypervolume were used to evaluate convergence and objective-space coverage under a common reference front.

As shown in Table 13, SMOWMA exhibited consistent performance over the 30 independent runs. Since lower GD, IGD, and SP values indicate better performance, the mean GD of 1.6618 × 10^−3^ suggests that the obtained solutions remained close to the numerical reference front. The mean IGD was 3.0941 × 10^−3^, with a standard deviation of 9.2409 × 10^−5^, indicating that the reference front was adequately represented by the approximation sets. Meanwhile, the mean SP of 1.9975 × 10^−3^ reflects a relatively uniform distribution of solutions along the trade-off front. For NHV, SMOWMA obtained a mean value of 0.731312 and a median value of 0.731317, while the best and worst values were 0.731518 and 0.731107, respectively. Under the same normalization scheme, the numerical reference front has an NHV of 0.734697. Therefore, the mean NHV obtained by SMOWMA corresponds to approximately 99.54% of the reference-front value. Moreover, the small NHV standard deviation of 9.1961 × 10^−5^ demonstrates limited variation in objective-space coverage among the 30 runs. The average runtime was 881.18 s, with a standard deviation of 73.52 s. Overall, the close agreement between the mean and median values, together with the narrow NHV range, indicates that SMOWMA provides stable convergence and coverage for the four-bar truss design problem.

**Figure 13:**
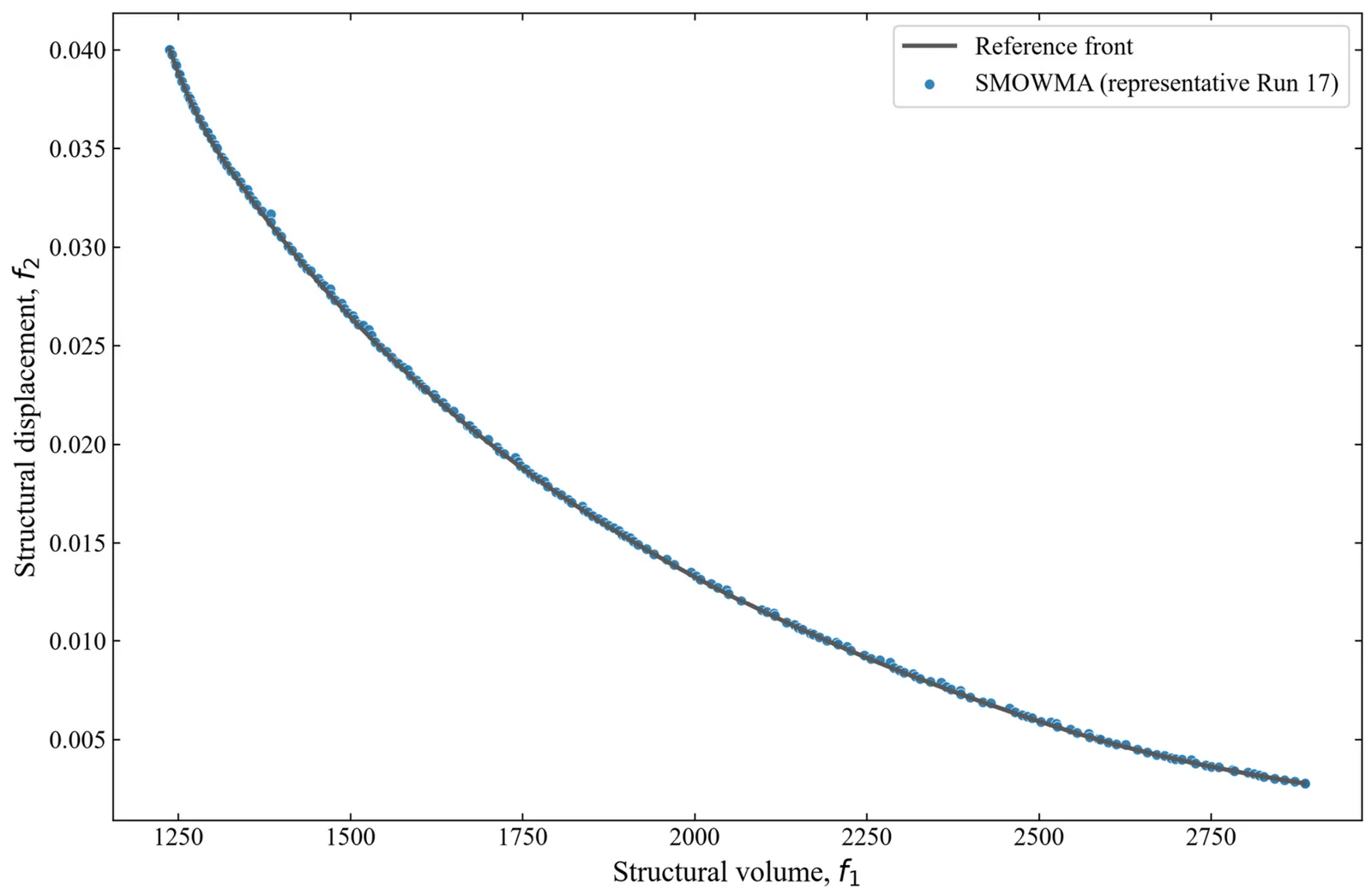
Reference and SMOWMA approximation fronts for the four-bar truss problem.

Figure 13 compares the numerical reference Pareto front with the representative approximation front obtained by SMOWMA. Run 17 was selected because its NHV value was closest to the median of the 30 independent runs, thereby representing the typical rather than the best performance of the algorithm. The obtained solutions closely overlap the reference front throughout the complete trade-off range and cover both extreme regions. The small deviation from the reference front indicates good convergence, while the continuous distribution of solutions demonstrates that SMOWMA maintains satisfactory diversity and objective-space coverage for the four-bar truss design problem.

### 5.4. Five-Objective Car Side-Impact Design Problem

The extended car side-impact design problem consists of seven continuous design variables, five minimization objectives, and ten engineering constraints. The design variables represent the thicknesses of seven structural components [42]. Based on the established car side-impact response model, the five-objective formulation considered in this study is summarized as

(44) $$\left\{\begin{array}{ccc} \min_{x}F(x) & = & \left[W(x),P_{p}(x),V_{MBP}(x),V_{FD}(x),D_{\max}(x)\right],\\ D_{\max}(x) & = & \max\left\{D_{U}(x),D_{M}(x),D_{L}(x)\right\},\\ W(x) & = & 1.98+4.9x_{1}+6.67x_{2}+6.98x_{3}+4.01x_{4}+1.78x_{5}+10^{-5}x_{6}+2.73x_{7}. \end{array}\right.$$

where W(x), P_p_(x), V_MBP_(x), and V_FD_(x) denote the structural weight, pubic symphysis force, B-pillar midpoint velocity, and front-door velocity at the B-pillar, respectively. D_U_(x), D_M_(x), and D_L_(x) represent the upper-, middle-, and lower-rib deflections, respectively. Therefore, D_max_(x) represents the worst rib-deflection response. All five components of F(x) are minimized simultaneously.

The design variables x_1_–x_7_ represent the thicknesses of the B-pillar inner panel, B-pillar reinforcement, floor-side inner panel, cross members, door beam, door-beltline reinforcement, and roof rail, respectively. Their allowable ranges are defined as

(45) $$0.5\leq x_{1},x_{3},x_{4}\leq 1.5,\;\;\;\;0.45\leq x_{2}\leq 1.35,\;\;\;\;0.875\leq x_{5}\leq 2.625,\;\;\;\;0.4\leq x_{6},x_{7}\leq 1.2.$$

(46) $$\left\{\begin{array}{ccc} g_{1}(x) & = & F_{A}(x)-1=1.16-0.3717x_{2}x_{4}-0.0092928x_{3}-1\leq 0,\\ g_{2}(x) & = & VC_{U}(x)-0.32=0.261-0.0159x_{1}x_{2}-0.06486x_{1}-0.019x_{2}x_{7}\\ & & +0.0144x_{3}x_{5}+0.0154464x_{6}-0.32\leq 0,\\ g_{3}(x) & = & VC_{M}(x)-0.32=0.214+0.00817x_{5}-0.045195x_{1}-0.0135168x_{1}\\ & & +0.03099x_{2}x_{6}-0.018x_{2}x_{7}+0.007176x_{3}+0.023232x_{3}-0.00364x_{5}x_{6}-0.018x_{2}^{2}-0.32\leq 0,\\ g_{4}(x) & = & VC_{L}(x)-0.32=0.74-0.61x_{2}-0.031296x_{3}-0.031872x_{7}+0.227x_{2}^{2}-0.32\leq 0,\\ g_{5}(x) & = & D_{U}(x)-32=28.98+3.818x_{3}-4.2x_{1}x_{2}+1.27296x_{6}-2.68065x_{7}-32\leq 0,\\ g_{6}(x) & = & D_{M}(x)-32=33.86+2.95x_{3}-5.057x_{1}x_{2}-3.795x_{2}-3.4431x_{7}+1.45728-32\leq 0,\\ g_{7}(x) & = & D_{L}(x)-32=46.36-9.9x_{2}-4.4505x_{1}-32\leq 0,\\ g_{8}(x) & = & P_{p}(x)-4=4.72-0.5x_{4}-0.19x_{2}x_{3}-4\leq 0,\\ g_{9}(x) & = & V_{MBP}(x)-9.9=10.58-0.674x_{1}x_{2}-0.67275x_{2}-9.9\leq 0,\\ g_{10}(x) & = & V_{FD}(x)-15.7=16.45-0.489x_{3}x_{7}-0.843x_{5}x_{6}-15.7\leq 0. \end{array}\right.$$

where F_A_ denotes the abdominal load, while VC_U_, VC_M_ and VC_L_ denote the upper-, middle-, and lower-rib viscous criteria, respectively. The remaining constraint functions impose upper limits on the three rib deflections, pubic symphysis force, B-pillar midpoint velocity, and front-door velocity.

A candidate solution was regarded as feasible only when all constraints in Equation (46) and the variable bounds in Equation (45) were satisfied. The feasibility handling, common normalization, NHV calculation, representative-run selection, and statistical testing procedures defined in Section 5.1 were applied consistently. MOEAD, MOWOA, MORBMO, SMOWMA, and NSGA-II were independently executed 30 times with a population size of 100.

#### 5.4.1. NHV Results and Statistical Comparison

Table 14 presents the NHV results obtained over 30 independent runs. Pairwise Wilcoxon rank-sum tests were conducted between SMOWMA and each comparison algorithm, and the resulting *p*-values were adjusted using the Holm procedure.

SMOWMA achieves the highest mean NHV of 0.403562, followed by NSGA-II and MORBMO. Compared with NSGA-II and MORBMO, the mean NHV obtained by SMOWMA increases by approximately 5.04% and 8.21%, respectively. In addition, its standard deviation is limited to 0.006436, indicating relatively consistent performance across the 30 independent runs.

All Holm-adjusted *p*-values are below 0.05. Therefore, the NHV differences between SMOWMA and the four comparison algorithms are statistically significant. The results indicate that SMOWMA can maintain favorable convergence, solution-set coverage, and run-to-run stability when the number of objectives increases to five.

#### 5.4.2. Representative Trade-Off

**Figure 14:**
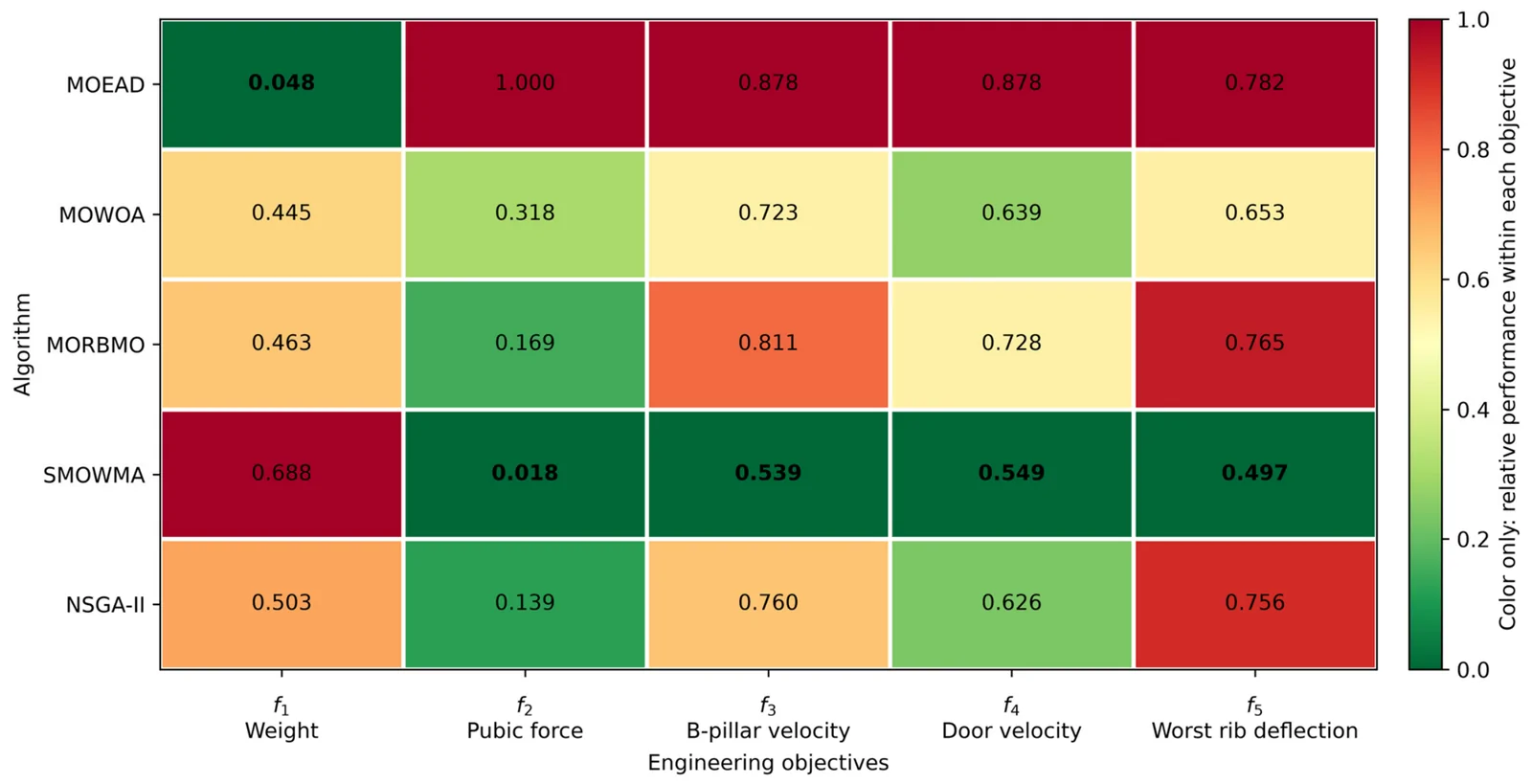
Normalized objective values of representative solutions for the five-objective car side-impact problem.

Figure 14 presents the commonly normalized objective values of one selected compromise solution for each algorithm. The same fixed normalization bounds were applied to all five algorithms. Since all five objectives are minimized, lower numerical values indicate better objective performance. The cell colors represent the relative performance within each objective of the five selected solutions, with greener cells indicating better ranks.

The selected solutions exhibit clear differences in objective preference. MOEAD obtains the lowest normalized structural weight, with f_1_ = 0.048, but records the highest normalized values for f_2_–f_5_. In contrast, SMOWMA achieves the lowest normalized values for pubic symphysis force, B-pillar velocity, front-door velocity, and worst rib deflection, with values of 0.018, 0.539, 0.549, and 0.497, respectively. However, its normalized structural weight is 0.688, which is the highest among the five selected solutions. MOWOA, MORBMO, and NSGA-II occupy intermediate positions, reflecting different compromises between structural weight and occupant-safety responses.

These differences illustrate the conflicting nature of the five engineering objectives. A representative solution that performs well for a particular objective does not necessarily correspond to the algorithm with the best overall approximation set. Therefore, the heatmap is used to illustrate the trade-off characteristics of individual representative solutions, whereas the overall algorithm comparison is determined from the NHV distributions reported in Table 14.

## 6. Conclusions

This study constructs SMOWMA as a Sobol-driven multi-objective optimization algorithm for constrained and multimodal engineering optimization problems, aiming to improve the stability and balance of the search process. The algorithm introduces a maximin scrambled Sobol initialization scheme and an archive-guided adaptive Student-*t* heavy-tailed flight strategy to improve initial population coverage and coordinate global exploration and local exploitation during the search. The method is not only inspired by the migration behavior of whales, but also takes into account the need to combine ranking, diversity preservation, and elitist retention within a unified multi-objective framework. The algorithm also combines fast non-dominated sorting, crowding distance, and an external archive to maintain the diversity of Pareto solutions and reduce the risk of premature convergence in complex search spaces. This paper evaluates the proposed method on commonly used benchmark test series such as ZDT, DTLZ, WFG, UF, and CF, and adopts HV, GD, IGD, and SP as performance indicators. In addition, three welded-beam design (WBD) models with two, three, and four objectives are also examined. The overall results show that, in terms of convergence and solution distribution, SMOWMA remains competitive with MOSFOA, MOWOA, MORBMO, MOWMA, SMS-EMOA, MOEAD, SPEA2, NSGA-II, and NSGA-III and achieves favorable results on many problems. Taken together, these findings indicate that SMOWMA maintains a favorable balance between convergence and solution diversity across benchmark problems with different characteristics.

The main outcomes of this article are as follows:

(1) SMOWMA combines the whale migration mechanism with maximin scrambled Sobol initialization, external-archive retention, and population updating based on rank and density information. The archive-guided adaptive Student-t flight further adjusts the leader search according to the optimization progress, archive-entry success feedback, and guide crowding distance. With fast non-dominated sorting and crowding distance, the search is able to move toward promising yet insufficiently explored regions while still preserving population spread under constrained conditions.
(2) On the ZDT, DTLZ, WFG, UF, and CF benchmark families, SMOWMA achieves favorable overall results for IGD, HV, and SP, whereas its GD performance and the results on individual problems are more varied. These findings support its overall competitiveness but do not imply uniform superiority across all problems and performance indicators.
(3) The welded-beam design problems with two, three, and four objectives further show that the method remains effective as the number of objectives increases. In these constrained engineering settings, SMOWMA maintains competitive set-level convergence and objective-space coverage, with its strongest NHV result obtained in the four-objective formulation. The bi-objective four-bar truss design problem provides additional structural-engineering validation, where SMOWMA obtains a well-distributed approximation front that closely follows the reference Pareto front. The five-objective car side-impact design problem further extends the validation, with SMOWMA achieving the best overall NHV performance among the compared algorithms.

There is still room to continue this work in several aspects. It would also be useful to test SMOWMA on many-objective problems with more than five objectives, where weaker dominance relations usually make the search more difficult. Another practical direction is to combine SMOWMA with surrogate models, especially for engineering problems in which high-fidelity simulations are expensive and the number of allowable evaluations is limited.

## Figures and Tables

**Table 1:** Mapping between whale-migration behavior and computational operators in SMOWMA.

No.	Biological Behavior (Humpback Whale Migration)	Computational Operator in SMOWMA	Mathematical Role	Degree of Biological Fidelity
1	A migratory pod of whales moving as a group	Population of candidate solutions	Solution set updated each generation	Metaphorical—fixed size, no demographics
2	Individual whale position in the ocean	Decision vector $W_{i}$ in the search space	Candidate solution encoding	Direct mapping of the position concept
3	More-experienced leader whales choosing the optimal route	Top-ranked individuals by Pareto rank and crowding distance	Leaders guide follower movement	Structural only—global ranking is not whale perception
4	Less-experienced follower whales relocating toward leaders	Follower-individual update using leader and leader-group mean	Exploitation operator	Metaphorical—leaders are a rank-ordered subset, not socially decided
5	Selection of non-dominated trade-off individuals as survivors	Fast non-dominated sorting (NDS)	Pareto ranking for environmental selection	No biological counterpart—added for MO handling
6	Maintenance of diverse survival strategies across the pod	Crowding-distance (CD) density estimator	Diversity preservation along the front	No biological counterpart—diversity engineering
7	Memory of superior routes across migration cycles	External Pareto archive	Elitist retention of non-dominated solutions	Structural analogy—archive is not whale memory
8	Leaders and followers guided by archive-derived search direction toward sparse front regions	Archive-guided migration (Algorithm 2)	Leader–follower update with archive-sparse prioritization	Metaphorical sparse region is a front-coverage concept, not a whale behavior

*Note: The degree-of-biological-fidelity column clarifies that these mappings are conceptual rather than empirically verified whale behaviors. Rows 5 and 6 have no direct biological counterpart, whereas rows 7 and 8 represent only structural or metaphorical analogies. Rows 5–8 are multi-objective components incorporated into the WMA framework to provide Pareto ranking, diversity preservation, elitist archiving, and archive-guided search.*

**Table 2:** Parameter settings for the compared algorithms.

Parameters	SMOWMA	MOSFOA	MOWOA	MORBMO	MOWMA	SMS-EMOA	MOEAD	SPEA2	NSGA-II	NSGA-III
Number of runs	30	30	30	30	30	30	30	30	30	30
Population size (N)	200	200	200	200	200	200	200	200	200	200
Maximum generations (T)	1000	1000	1000	1000	1000	1000	1000	1000	1000	1000
Archive size	200	200	200	200	200	200	200	200	200	200

**Table 3:** Reference points used for HV calculation.

Benchmark Problems	Reference Point
ZDT1–ZDT4 and ZDT6	(1.1,1.1)
WFG1–WFG9	(2.2,4.4)
DTLZ1	(0.55,0.55,0.55)
DTLZ2–DTLZ4	(1.1,1.1,1.1)
DTLZ5–DTLZ6	(0.777817,0.777817,1.1)
UF1–UF7	(1.1,1.1)
CF1–CF7	(1.1,1.1)
CF8	(0.872414,0.872414,1.1)
CF9	(1.1,1.1,1.043390)
CF10	(1.035783,1.1,1.1)

**Table 4:** Sensitivity analysis of the main parameters in SMOWMA.

Parameter	Value	Mean HV Rank ↓	Mean IGD Rank ↓	Mean Combined Rank ↓	Composite Score ↑
*K*	1	2.6	3.2	2.9	0.599
	3	2.8	3.0	2.9	0.784
	**5**	**2.6**	**2.4**	**2.5**	**0.835**
	7	4.0	4.4	4.2	0.441
	9	3.0	**2.0**	**2.5**	0.713
*ν_max_*	5	4.0	3.4	3.7	0.332
	10	4.0	4.2	4.1	0.357
	20	3.2	3.6	3.4	0.477
	**30**	**1.6**	**1.8**	**1.7**	**0.788**
	50	2.2	2.0	2.1	0.553
*ω*	0.1	3.2	2.8	3.0	0.371
	0.3	3.2	3.6	3.4	0.428
	**0.5**	**1.8**	**1.8**	**1.8**	**0.682**
	0.7	3.0	2.8	2.9	0.393
	0.9	3.8	4.0	3.9	0.212

*Note: ↓ and ↑ indicate lower-is-better and higher-is-better, respectively. Boldface identifies the recommended setting in each parameter group; for K = 9, the best IGD rank and the tied, best combined rank are additionally highlighted in bold.*

**Table 5:** Experimental variants used in the component ablation study.

Method	Experimental Role	Initialization Strategy	Leader Flight Strategy	Archive-Guided Selection	Archive-Success Feedback	Main Purpose
SMOWMA-RI	MSSI ablation	Random initialization	Adaptive Student-*t*	Crowding-weighted	✓	Evaluate the overall contribution of MSSI
SMOWMA-Sobol	MSSI internal ablation	Scrambled Sobol	Adaptive Student-*t*	Crowding-weighted	✓	Isolate the additional contribution of maximin selection
SMOWMA-LF	ASTF replacement variant	Maximin Scrambled Sobol	Lévy flight	Crowding-weighted	✓	Compare Lévy flight with the proposed ASTF
SMOWMA-FixedT	ASTF internal ablation	Maximin Scrambled Sobol	Fixed Student-*t*	Crowding-weighted	✓	Isolate the contribution of the adaptive Student-*t* mechanism
SMOWMA-Uniform	AGS internal ablation	Maximin Scrambled Sobol	Adaptive Student-*t*	Uniform guide selection	✓	Evaluate the contribution of crowding-weighted guide selection
SMOWMA-NoRho	AGS internal ablation	Maximin Scrambled Sobol	Adaptive Student-*t*	Crowding-weighted	×	Evaluate the contribution of $\bar{\rho }$-based feedback
SMOWMA-NoAG	AGS ablation	Maximin Scrambled Sobol	Adaptive Student-*t*	Uniform guide selection	×	Assess the combined contribution of AGS
SMOWMA	Full algorithm	Maximin Scrambled Sobol	Adaptive Student-*t*	Crowding-weighted	✓	Complete proposed algorithm

*Note: MSSI denotes maximin scrambled Sobol initialization; ASTF denotes adaptive Student-t flight; AGS denotes the archive-guided strategy combining crowding-weighted guide selection and $\bar{\rho }$-based feedback. “✓” indicates that the corresponding mechanism is enabled, whereas “×” indicates that it is disabled.*

**Table 6:** Summary of the HV-based ablation results over 15 benchmark problems.

Method	Mean HV Rank ↓	Best-HV Cases ↑	Full SMOWMA vs. Variant (W/T/L)
SMOWMA-RI	5.400	0/15	15/0/0
SMOWMA-Sobol	4.667	0/15	15/0/0
SMOWMA-LF	6.100	0/15	15/0/0
SMOWMA-FixedT	4.633	0/15	15/0/0
SMOWMA-Uniform	4.767	0/15	15/0/0
SMOWMA-NoRho	5.067	0/15	15/0/0
SMOWMA-NoAG	4.367	0/15	15/0/0
SMOWMA	**1.000**	**15/15**	**—**

*Note: A lower mean HV rank indicates better overall performance. “Best-HV cases” denotes the number of problems on which a method obtains the highest mean HV among the eight configurations. W/T/L gives the numbers of problems on which the full SMOWMA obtains a higher, equal, or lower mean HV than the corresponding ablation variant, respectively. Detailed mean ± standard deviation values are reported in Table A4. “↑” and “↓” indicate that higher and lower values are preferred, respectively.*

**Table 7:** HV results for the benchmark problems.

	**ZDT1**		**ZDT2**		**ZDT3**	
Algorithms	Ave	Std	Ave	Std	Ave	Std
SMOWMA	**8.6656 × 10^−1^**	6.6818 × 10^−4^	**5.4062 × 10^−1^**	**8.8848 × 10^−5^**	**1.0244 × 10^0^**	**5.3845 × 10^−5^**
MOSFOA	8.3606 × 10^−1^	1.5434 × 10^−1^	3.6748 × 10^−1^	2.2160 × 10^−1^	9.9681 × 10^−1^	1.1053 × 10^−2^
MOWOA	8.4912 × 10^−1^	1.6503 × 10^−3^	5.3681 × 10^−1^	4.1159 × 10^−4^	1.0034 × 10^0^	3.2898 × 10^−3^
MORBMO	8.4748 × 10^−1^	5.3514 × 10^−3^	5.3691 × 10^−1^	6.5908 × 10^−4^	9.7226 × 10^−1^	6.1230 × 10^−2^
MOWMA	8.6346 × 10^−1^	**3.3200 × 10^−4^**	2.0398 × 10^−1^	1.3286 × 10^−2^	1.0049 × 10^0^	1.9740 × 10^−2^
SMS-EMOA	8.5746 × 10^−1^	1.8784 × 10^−3^	3.0542 × 10^−1^	1.9007 × 10^−1^	1.0033 × 10^0^	1.2486 × 10^−2^
MOEAD	6.8909 × 10^−1^	6.6581 × 10^−2^	1.7471 × 10^−1^	1.1993 × 10^−1^	7.8284 × 10^−1^	1.1259 × 10^−1^
SPEA2	8.4898 × 10^−1^	3.1495 × 10^−3^	4.6176 × 10^−1^	8.4380 × 10^−2^	9.9485 × 10^−1^	1.0675 × 10^−2^
NSGA-II	8.5076 × 10^−1^	2.8968 × 10^−3^	4.5624 × 10^−1^	8.9273 × 10^−2^	9.9831 × 10^−1^	1.5900 × 10^−2^
NSGA-III	8.4139 × 10^−1^	1.5802 × 10^−2^	3.2783 × 10^−1^	1.7960 × 10^−1^	9.8302 × 10^−1^	2.1872 × 10^−2^
	ZDT4		ZDT6		DTLZ1	
Algorithms	Ave	Std	Ave	Std	Ave	Std
SMOWMA	**8.7367 × 10^−1^**	**8.2648 × 10^−5^**	**5.0681 × 10^−1^**	**6.5066 × 10^−5^**	1.3937 × 10^−1^	2.2766 × 10^−4^
MOSFOA	7.9611 × 10^−1^	2.4107 × 10^−1^	5.0511 × 10^−1^	4.1217 × 10^−4^	2.6907 × 10^−2^	4.1006 × 10^−2^
MOWOA	7.8261 × 10^−1^	2.7550 × 10^−1^	5.0343 × 10^−1^	2.1466 × 10^−4^	1.1231 × 10^−1^	5.8934 × 10^−2^
MORBMO	0.0000 × 10^0^	0.0000 × 10^0^	5.0446 × 10^−1^	6.6003 × 10^−4^	1.4037 × 10^−1^	6.1026 × 10^−4^
MOWMA	0.0000 × 10^0^	0.0000 × 10^0^	4.9712 × 10^−1^	2.7402 × 10^−3^	0.0000 × 10^0^	0.0000 × 10^0^
SMS-EMOA	5.5941 × 10^−1^	2.5389 × 10^−1^	4.8331 × 10^−1^	6.3801 × 10^−3^	1.4201 × 10^−1^	**1.2384 × 10^−5^**
MOEAD	7.9346 × 10^−1^	9.1254 × 10^−2^	4.9877 × 10^−1^	1.3194 × 10^−3^	**1.4294 × 10^−1^**	1.3028 × 10^−4^
SPEA2	5.0387 × 10^−1^	2.1329 × 10^−1^	4.7515 × 10^−1^	4.1294 × 10^−3^	1.4084 × 10^−1^	2.1246 × 10^−4^
NSGA-II	5.9281 × 10^−1^	2.1492 × 10^−1^	4.7713 × 10^−1^	8.8562 × 10^−3^	1.3945 × 10^−1^	5.6733 × 10^−4^
NSGA-III	5.8718 × 10^−1^	1.4938 × 10^−1^	4.6829 × 10^−1^	9.5350 × 10^−3^	1.4193 × 10^−1^	2.1939 × 10^−5^
	DTLZ2		DTLZ3		DTLZ4	
Algorithms	Ave	Std	Ave	Std	Ave	Std
SMOWMA	**7.9645 × 10^−1^**	2.7761 × 10^−3^	7.4291 × 10^−1^	**2.6808 × 10^−3^**	**7.8969 × 10^−1^**	4.6511 × 10^−3^
MOSFOA	7.1955 × 10^−1^	3.9238 × 10^−3^	0.0000 × 10^0^	0.0000 × 10^0^	7.1836 × 10^−1^	2.6387 × 10^−3^
MOWOA	7.3535 × 10^−1^	4.5387 × 10^−3^	0.0000 × 10^0^	0.0000 × 10^0^	7.3479 × 10^−1^	5.4625 × 10^−3^
MORBMO	7.4152 × 10^−1^	2.9249 × 10^−3^	7.3014 × 10^−1^	3.7694 × 10^−3^	7.4083 × 10^−1^	4.4034 × 10^−3^
MOWMA	7.3246 × 10^−1^	4.1536 × 10^−3^	0.0000 × 10^0^	0.0000 × 10^0^	7.4896 × 10^−1^	1.4947 × 10^−3^
SMS-EMOA	7.7229 × 10^−1^	4.9868 × 10^−5^	7.4840 × 10^−1^	4.1914 × 10^−3^	7.7233 × 10^−1^	6.4359 × 10^−5^
MOEAD	7.6516 × 10^−1^	**1.1676 × 10^−6^**	6.5692 × 10^−1^	4.3631 × 10^−2^	6.1387 × 10^−1^	1.6012 × 10^−1^
SPEA2	7.5195 × 10^−1^	3.5092 × 10^−3^	7.4500 × 10^−1^	7.3448 × 10^−3^	7.5940 × 10^−1^	1.0361 × 10^−3^
NSGA-II	7.3860 × 10^−1^	4.9193 × 10^−3^	7.4060 × 10^−1^	3.3767 × 10^−3^	7.4297 × 10^−1^	5.8351 × 10^−3^
NSGA-III	7.6496 × 10^−1^	5.3981 × 10^−6^	**7.5058 × 10^−1^**	2.9611 × 10^−3^	7.6495 × 10^−1^	**3.8441 × 10^−5^**
	DTLZ5		DTLZ6		WFG1	
Algorithms	Ave	Std	Ave	Std	Ave	Std
SMOWMA	1.3345 × 10^−1^	4.0362 × 10^−5^	1.2906 × 10^−1^	6.2800 × 10^−4^	2.5851 × 10^0^	7.7061 × 10^−2^
MOSFOA	1.3329 × 10^−1^	8.5487 × 10^−5^	**1.3061 × 10^−1^**	**4.3000 × 10^−4^**	**5.7040 × 10^0^**	1.9708 × 10^−1^
MOWOA	**1.3468 × 10^−1^**	4.3507 × 10^−5^	1.2693 × 10^−1^	4.8800 × 10^−4^	2.2488 × 10^0^	7.2074 × 10^−2^
MORBMO	1.3419 × 10^−1^	3.4043 × 10^−5^	1.3040 × 10^−1^	4.4500 × 10^−4^	2.2110 × 10^0^	7.2597 × 10^−2^
MOWMA	1.1862 × 10^−1^	1.3823 × 10^−3^	1.3019 × 10^−1^	2.3990 × 10^−3^	2.1772 × 10^0^	**3.7033 × 10^−2^**
SMS-EMOA	1.3409 × 10^−1^	6.8782 × 10^−6^	8.4868 × 10^−2^	1.5649 × 10^−2^	1.9344 × 10^0^	6.0486 × 10^−1^
MOEAD	1.2574 × 10^−1^	**2.0254 × 10^−6^**	7.9354 × 10^−2^	1.7434 × 10^−2^	3.0140 × 10^0^	8.0321 × 10^−1^
SPEA2	1.3357 × 10^−1^	3.9002 × 10^−4^	7.5583 × 10^−2^	1.7677 × 10^−2^	2.1211 × 10^0^	7.6076 × 10^−1^
NSGA-II	1.3386 × 10^−1^	4.3471 × 10^−5^	7.8837 × 10^−2^	1.5255 × 10^−2^	2.4089 × 10^0^	4.2498 × 10^−1^
NSGA-III	1.2501 × 10^−1^	5.5801 × 10^−5^	5.3890 × 10^−2^	1.3430 × 10^−2^	2.1450 × 10^0^	4.1674 × 10^−1^
	WFG2		WFG3		WFG4	
Algorithms	Ave	Std	Ave	Std	Ave	Std
SMOWMA	**6.1487 × 10^0^**	6.9631 × 10^−4^	5.6269 × 10^0^	**1.6305 × 10^−3^**	**2.9114 × 10^0^**	**4.2859 × 10^−2^**
MOSFOA	6.1433 × 10^0^	**4.6469 × 10^−4^**	5.6384 × 10^0^	1.7416 × 10^−3^	2.5187 × 10^0^	9.3302 × 10^−2^
MOWOA	6.1100 × 10^0^	2.0204 × 10^−2^	5.6411 × 10^0^	1.2919 × 10^−2^	2.4805 × 10^0^	9.3526 × 10^−2^
MORBMO	6.1409 × 10^0^	1.1408 × 10^−3^	5.6414 × 10^0^	3.1595 × 10^−3^	2.5978 × 10^0^	6.5829 × 10^−2^
MOWMA	6.1022 × 10^0^	1.1711 × 10^−3^	5.6010 × 10^0^	1.7624 × 10^−3^	2.3106 × 10^0^	1.0584 × 10^−1^
SMS-EMOA	5.8352 × 10^0^	1.0771 × 10^−1^	**5.6549 × 10^0^**	4.2150 × 10^−2^	2.6720 × 10^0^	1.1217 × 10^−1^
MOEAD	5.5450 × 10^0^	1.3536 × 10^−1^	5.6114 × 10^0^	1.0345 × 10^−1^	2.4576 × 10^0^	1.4205 × 10^−1^
SPEA2	5.7139 × 10^0^	1.9480 × 10^−1^	5.6475 × 10^0^	3.9720 × 10^−2^	2.6588 × 10^0^	7.7622 × 10^−2^
NSGA-II	5.8411 × 10^0^	7.1908 × 10^−2^	5.6506 × 10^0^	6.8132 × 10^−2^	2.6695 × 10^0^	7.8807 × 10^−2^
NSGA-III	5.8166 × 10^0^	6.6508 × 10^−2^	5.6500 × 10^0^	5.6083 × 10^−2^	2.6338 × 10^0^	8.8387 × 10^−2^
	WFG5		WFG6		WFG7	
Algorithms	Ave	Std	Ave	Std	Ave	Std
SMOWMA	2.9612 × 10^0^	5.5142 × 10^−3^	**3.3736 × 10^0^**	**1.7958 × 10^−3^**	3.3589 × 10^0^	1.2913 × 10^−2^
MOSFOA	3.0066 × 10^0^	1.4582 × 10^−2^	3.3649 × 10^0^	1.8398 × 10^−3^	**3.3651 × 10^0^**	1.9216 × 10^−3^
MOWOA	**3.0106 × 10^0^**	1.8767 × 10^−2^	3.3625 × 10^0^	2.8506 × 10^−3^	3.3378 × 10^0^	3.0660 × 10^−3^
MORBMO	2.9765 × 10^0^	1.4743 × 10^−2^	3.3681 × 10^0^	1.9894 × 10^−3^	3.3567 × 10^0^	**1.6919 × 10^−3^**
MOWMA	2.9802 × 10^0^	4.8649 × 10^−3^	3.2960 × 10^0^	1.8406 × 10^−3^	3.3322 × 10^0^	2.2566 × 10^−3^
SMS-EMOA	3.0001 × 10^0^	3.9327 × 10^−3^	3.0928 × 10^0^	2.0950 × 10^−1^	3.3172 × 10^0^	1.4609 × 10^−2^
MOEAD	2.9744 × 10^0^	**3.8517 × 10^−3^**	2.9115 × 10^0^	2.4525 × 10^−1^	3.3103 × 10^0^	5.8127 × 10^−2^
SPEA2	3.0071 × 10^0^	8.3421 × 10^−3^	3.0813 × 10^0^	2.0596 × 10^−1^	3.2736 × 10^0^	1.6924 × 10^−2^
NSGA-II	2.9990 × 10^0^	6.7554 × 10^−3^	3.1604 × 10^0^	1.5795 × 10^−1^	3.3141 × 10^0^	2.8703 × 10^−2^
NSGA-III	2.9973 × 10^0^	8.0318 × 10^−3^	3.0222 × 10^0^	1.5785 × 10^−1^	3.3081 × 10^0^	2.1701 × 10^−2^
	WFG8		WFG9		UF1	
Algorithms	Ave	Std	Ave	Std	Ave	Std
SMOWMA	**3.3231 × 10^0^**	9.8887 × 10^−2^	**3.2950 × 10^0^**	1.1063 × 10^−2^	7.7705 × 10^−1^	**3.9165 × 10^−3^**
MOSFOA	3.2880 × 10^0^	1.7102 × 10^−1^	3.2920 × 10^0^	**4.8494 × 10^−3^**	**8.2083 × 10^−1^**	3.4977 × 10^−2^
MOWOA	2.3964 × 10^0^	**9.4569 × 10^−3^**	3.2251 × 10^0^	1.0922 × 10^−2^	7.4874 × 10^−1^	2.8770 × 10^−2^
MORBMO	2.2991 × 10^0^	1.9782 × 10^−2^	3.2301 × 10^0^	6.8043 × 10^−3^	7.7004 × 10^−1^	9.5091 × 10^−3^
MOWMA	2.8373 × 10^0^	1.8446 × 10^−1^	3.2785 × 10^0^	5.5864 × 10^−2^	6.7510 × 10^−1^	5.5122 × 10^−3^
SMS-EMOA	2.2563 × 10^0^	2.5885 × 10^−1^	3.2468 × 10^0^	1.2503 × 10^−2^	7.2305 × 10^−1^	1.4364 × 10^−2^
MOEAD	2.2234 × 10^0^	1.1420 × 10^−1^	3.1928 × 10^0^	8.4540 × 10^−2^	5.8174 × 10^−1^	5.1008 × 10^−2^
SPEA2	2.2177 × 10^0^	3.0611 × 10^−1^	3.2861 × 10^0^	8.0862 × 10^−3^	7.3798 × 10^−1^	1.7911 × 10^−2^
NSGA-II	2.2380 × 10^0^	2.6935 × 10^−1^	3.2922 × 10^0^	1.1253 × 10^−2^	7.3075 × 10^−1^	2.9017 × 10^−2^
NSGA-III	2.1713 × 10^0^	2.5772 × 10^−1^	3.2862 × 10^0^	1.7924 × 10^−2^	7.1255 × 10^−1^	3.6975 × 10^−2^
	UF2		UF3		UF4	
Algorithms	Ave	Std	Ave	Std	Ave	Std
SMOWMA	**8.6294 × 10^−1^**	2.2742 × 10^−3^	**7.3294 × 10^−1^**	1.7805 × 10^−2^	4.3136 × 10^−1^	6.8336 × 10^−3^
MOSFOA	8.5504 × 10^−1^	1.3406 × 10^−3^	6.4309 × 10^−1^	4.2784 × 10^−2^	**5.0106 × 10^−1^**	2.3515 × 10^−2^
MOWOA	8.1502 × 10^−1^	5.8207 × 10^−3^	6.6485 × 10^−1^	3.7937 × 10^−2^	4.0094 × 10^−1^	**2.2265 × 10^−3^**
MORBMO	8.4711 × 10^−1^	**9.8454 × 10^−4^**	4.1158 × 10^−1^	**1.5111 × 10^−2^**	4.2151 × 10^−1^	1.0721 × 10^−2^
MOWMA	7.3951 × 10^−1^	3.2328 × 10^−3^	1.2199 × 10^−1^	1.7418 × 10^−2^	3.6204 × 10^−1^	3.2135 × 10^−3^
SMS-EMOA	8.1740 × 10^−1^	8.8552 × 10^−3^	4.9456 × 10^−1^	3.1829 × 10^−2^	3.8616 × 10^−1^	2.8635 × 10^−2^
MOEAD	7.3546 × 10^−1^	4.7554 × 10^−2^	4.2184 × 10^−1^	4.9914 × 10^−2^	2.9700 × 10^−1^	3.9716 × 10^−2^
SPEA2	8.2789 × 10^−1^	6.0832 × 10^−3^	5.3484 × 10^−1^	3.7541 × 10^−2^	3.4790 × 10^−1^	5.3695 × 10^−2^
NSGA-II	8.2725 × 10^−1^	4.2837 × 10^−3^	5.5679 × 10^−1^	5.4467 × 10^−2^	3.8436 × 10^−1^	3.2805 × 10^−2^
NSGA-III	8.2059 × 10^−1^	5.0107 × 10^−3^	5.0653 × 10^−1^	2.6044 × 10^−2^	3.8362 × 10^−1^	3.4231 × 10^−2^
	UF5		IIIUF6		UF7	
Algorithms	Ave	Std	Ave	Std	Ave	Std
SMOWMA	**3.4473 × 10^−1^**	5.9443 × 10^−2^	**4.7012 × 10^−1^**	**3.9800 × 10^−3^**	6.6679 × 10^−1^	**1.8613 × 10^−3^**
MOSFOA	3.3629 × 10^−1^	9.6916 × 10^−2^	4.1600 × 10^−1^	4.5735 × 10^−2^	**6.8595 × 10^−1^**	1.0052 × 10^−2^
MOWOA	0.0000 × 10^0^	0.0000 × 10^0^	3.0889 × 10^−1^	1.0476 × 10^−1^	6.3400 × 10^−1^	9.6789 × 10^−3^
MORBMO	0.0000 × 10^0^	0.0000 × 10^0^	2.1318 × 10^−1^	4.4564 × 10^−2^	6.7006 × 10^−1^	3.7765 × 10^−3^
MOWMA	0.0000 × 10^0^	0.0000 × 10^0^	1.9489 × 10^−2^	9.7796 × 10^−3^	5.5347 × 10^−1^	4.2877 × 10^−3^
SMS-EMOA	1.5784 × 10^−1^	1.1000 × 10^−1^	3.7057 × 10^−1^	6.3713 × 10^−2^	5.3905 × 10^−1^	1.1870 × 10^−1^
MOEAD	1.0567 × 10^−2^	**1.5768 × 10^−2^**	1.9374 × 10^−1^	1.0202 × 10^−1^	2.8202 × 10^−1^	1.4781 × 10^−1^
SPEA2	7.9299 × 10^−2^	5.9280 × 10^−2^	3.9109 × 10^−1^	4.9076 × 10^−2^	5.8417 × 10^−1^	8.4147 × 10^−2^
NSGA-II	9.5857 × 10^−2^	7.1253 × 10^−2^	3.3666 × 10^−1^	1.6556 × 10^−2^	6.2871 × 10^−1^	2.1503 × 10^−2^
NSGA-III	1.9569 × 10^−1^	5.5900 × 10^−2^	3.8477 × 10^−1^	6.7631 × 10^−2^	5.0392 × 10^−1^	1.2264 × 10^−1^
	CF1		CF2		CF3	
Algorithms	Ave	Std	Ave	Std	Ave	Std
SMOWMA	**7.0738 × 10^−1^**	2.9539 × 10^−3^	**7.7501 × 10^−1^**	1.5322 × 10^−2^	**2.2240 × 10^−1^**	4.6632 × 10^−2^
MOSFOA	6.7333 × 10^−1^	1.3483 × 10^−3^	6.1703 × 10^−1^	**4.9917 × 10^−5^**	2.0301 × 10^−1^	4.0742 × 10^−2^
MOWOA	6.7823 × 10^−1^	1.6366 × 10^−3^	6.1651 × 10^−1^	2.5318 × 10^−4^	3.0457 × 10^−2^	4.6133 × 10^−2^
MORBMO	6.7524 × 10^−1^	8.4083 × 10^−4^	5.5197 × 10^−1^	5.2498 × 10^−2^	1.2181 × 10^−1^	4.3958 × 10^−2^
MOWMA	6.7202 × 10^−1^	1.5330 × 10^−3^	6.0719 × 10^−1^	1.8034 × 10^−3^	0.0000 × 10^0^	0.0000 × 10^0^
SMS-EMOA	7.0598 × 10^−1^	6.5895 × 10^−6^	5.6135 × 10^−1^	4.9042 × 10^−2^	1.1933 × 10^−1^	5.9085 × 10^−2^
MOEAD	6.2808 × 10^−1^	2.6209 × 10^−2^	8.9121 × 10^−2^	5.2356 × 10^−2^	1.1329 × 10^−2^	**2.2885 × 10^−2^**
SPEA2	7.0653 × 10^−1^	2.0449 × 10^−4^	1.8524 × 10^−2^	2.5646 × 10^−2^	1.0929 × 10^−1^	4.1466 × 10^−2^
NSGA-II	7.0673 × 10^−1^	8.4910 × 10^−5^	2.5464 × 10^−2^	1.2548 × 10^−2^	1.2304 × 10^−1^	5.6270 × 10^−2^
NSGA-III	7.0125 × 10^−1^	**2.6939 × 10^−7^**	5.6279 × 10^−1^	3.9815 × 10^−2^	1.3785 × 10^−1^	5.5856 × 10^−2^
	CF4		CF5		CF6	
Algorithms	Ave	Std	Ave	Std	Ave	Std
SMOWMA	**5.5967 × 10^−1^**	2.7670 × 10^−4^	2.5921 × 10^−1^	9.8494 × 10^−2^	**4.3384 × 10^−1^**	5.5179 × 10^−4^
MOSFOA	5.5881 × 10^−1^	5.0731 × 10^−5^	**3.0729 × 10^−1^**	4.1487 × 10^−2^	4.2263 × 10^−1^	1.0118 × 10^−3^
MOWOA	5.4404 × 10^−1^	8.6623 × 10^−3^	1.7163 × 10^−1^	4.7812 × 10^−2^	4.2794 × 10^−1^	8.3236 × 10^−4^
MORBMO	5.5925 × 10^−1^	**1.5742 × 10^−5^**	1.6180 × 10^−1^	6.2701 × 10^−2^	4.2368 × 10^−1^	4.0319 × 10^−4^
MOWMA	5.4215 × 10^−1^	3.4133 × 10^−3^	1.0977 × 10^−1^	**3.2546 × 10^−3^**	4.1584 × 10^−1^	1.7104 × 10^−3^
SMS-EMOA	5.5628 × 10^−1^	4.6305 × 10^−3^	2.5171 × 10^−1^	4.1960 × 10^−2^	4.2927 × 10^−1^	6.0199 × 10^−4^
MOEAD	5.1550 × 10^−1^	1.6568 × 10^−2^	4.1789 × 10^−2^	5.2502 × 10^−2^	4.2166 × 10^−1^	6.1513 × 10^−3^
SPEA2	5.5873 × 10^−1^	1.0882 × 10^−4^	1.6847 × 10^−1^	5.6689 × 10^−2^	4.2985 × 10^−1^	**3.6787 × 10^−4^**
NSGA-II	5.5892 × 10^−1^	2.3364 × 10^−4^	1.8189 × 10^−1^	7.0938 × 10^−2^	4.2954 × 10^−1^	5.0435 × 10^−4^
NSGA-III	5.5595 × 10^−1^	3.6840 × 10^−3^	2.4007 × 10^−1^	6.1631 × 10^−2^	4.1354 × 10^−1^	1.1640 × 10^−2^
	CF7		CF8		CF9	
Algorithms	Ave	Std	Ave	Std	Ave	Std
SMOWMA	3.1242 × 10^−1^	7.3084 × 10^−2^	1.8729 × 10^−3^	4.1880 × 10^−3^	**7.6907 × 10^−1^**	4.7275 × 10^−3^
MOSFOA	**3.5262 × 10^−1^**	3.1439 × 10^−2^	7.0768 × 10^−1^	1.9589 × 10^−2^	6.0313 × 10^−1^	1.3889 × 10^−2^
MOWOA	5.5363 × 10^−2^	6.1066 × 10^−2^	0.0000 × 10^0^	0.0000 × 10^0^	6.8146 × 10^−1^	1.2357 × 10^−2^
MORBMO	3.1225 × 10^−1^	7.3050 × 10^−2^	0.0000 × 10^0^	0.0000 × 10^0^	7.2691 × 10^−1^	3.9234 × 10^−3^
MOWMA	7.5633 × 10^−3^	**1.0907 × 10^−2^**	0.0000 × 10^0^	0.0000 × 10^0^	5.5064 × 10^−1^	9.1221 × 10^−3^
SMS-EMOA	2.8490 × 10^−1^	4.4523 × 10^−2^	6.0248 × 10^−1^	5.7904 × 10^−3^	6.9478 × 10^−1^	**1.2171 × 10^−4^**
MOEAD	1.5601 × 10^−2^	3.3199 × 10^−2^	0.0000 × 10^0^	0.0000 × 10^0^	7.5545 × 10^−1^	1.6120 × 10^−4^
SPEA2	3.2033 × 10^−1^	4.3330 × 10^−2^	6.2904 × 10^−1^	4.6432 × 10^−1^	6.7760 × 10^−1^	3.6732 × 10^−3^
NSGA-II	2.8871 × 10^−1^	9.1130 × 10^−2^	7.3178 × 10^−1^	4.8563 × 10^−1^	6.6352 × 10^−1^	6.6199 × 10^−3^
NSGA-III	3.1165 × 10^−1^	4.3564 × 10^−2^	**7.6870 × 10^−1^**	**1.5300 × 10^−4^**	6.8790 × 10^−1^	8.4601 × 10^−4^
	CF10					
Algorithms	Ave	Std				
SMOWMA	7.0773 × 10^−1^	2.4813 × 10^−3^				
MOSFOA	6.5415 × 10^−1^	2.8741 × 10^−3^				
MOWOA	7.0171 × 10^−1^	3.2361 × 10^−3^				
MORBMO	7.0417 × 10^−1^	5.3185 × 10^−3^				
MOWMA	6.9542 × 10^−1^	4.2486 × 10^−3^				
SMS-EMOA	6.6999 × 10^−1^	**6.9054 × 10^−5^**				
MOEAD	**7.2848 × 10^−1^**	8.1218 × 10^−4^				
SPEA2	6.4858 × 10^−1^	1.3083 × 10^−3^				
NSGA-II	6.3445 × 10^−1^	4.4849 × 10^−3^				
NSGA-III	6.5748 × 10^−1^	2.6775 × 10^−4^				

**Table 8:** Average ranks and Friedman test results across the benchmark problems.

Algorithm	GD	IGD	HV	SP
SMOWMA	4.162	**3.568**	**2.324**	**3.216**
MOSFOA	5.297	3.757	4.216	5.216
MOWOA	6.297	6.730	5.500	5.297
MORBMO	6.162	6.135	5.270	5.162
MOWMA	7.824	6.784	7.514	8.027
SMS-EMOA	**3.730**	5.514	5.135	5.973
MOEAD	6.270	8.081	7.797	5.081
SPEA2	4.838	4.865	5.892	4.865
NSGA-II	4.851	4.703	5.243	5.541
NSGA-III	5.568	4.865	6.108	6.622
Friedman χ^2^	52.071	74.437	88.4295	56.104
*p*-value	4.3819 × 10^−8^	2.0412 × 10^−12^	3.3617 × 10^−15^	7.4994 × 10^−9^

*Note: A smaller average rank indicates better performance. The lowest average rank for each metric is shown in bold. The 37 benchmark problems were treated as paired blocks in each Friedman test.*

**Table 9:** Holm-adjusted *p*-values for pairwise comparisons between SMOWMA and each competing algorithm.

SMOWMA vs.	GD	IGD	HV	SP
MOSFOA	0.0654 (=)	0.8980 (=)	0.0395 (+)	0.0012 (+)
MOWOA	0.0070 (+)	3.2391 × 10^−5^ (+)	1.0643 × 10^−5^ (+)	0.0022 (+)
MORBMO	0.0110 (+)	0.0020 (+)	5.7113 × 10^−5^ (+)	0.0019 (+)
MOWMA	3.3207 × 10^−4^ (+)	4.6188 × 10^−4^ (+)	2.5140 × 10^−6^ (+)	4.8623 × 10^−5^ (+)
SMS-EMOA	0.4363 (=)	0.0061 (+)	1.0365 × 10^−4^ (+)	0.0013 (+)
MOEAD	0.0165 (+)	7.0037 × 10^−6^ (+)	3.5198 × 10^−5^ (+)	0.0193 (+)
SPEA2	0.4363 (=)	0.0331 (+)	1.0365 × 10^−4^ (+)	0.0214 (+)
NSGA-II	0.4363 (=)	0.0225 (+)	5.8645 × 10^−5^ (+)	7.7016 × 10^−4^ (+)
NSGA-III	0.1467 (=)	0.0331 (+)	7.0813 × 10^−5^ (+)	0.0012 (+)

Note: “+” indicates that SMOWMA performs significantly better than the compared algorithm, while “=” indicates that no significant difference is detected after Holm correction at α=0.05.

**Table 10:** Execution-time comparison under equal function-evaluation budgets.

Algorithm	ZDT1 (s)	DTLZ2 (s)	WFG2 (s)	UF3 (s)	Mean (s)
NSGA-II	3.338 ± 0.501	17.50 ± 0.83	2.810 ± 0.171	47.94 ± 2.73	17.897
NSGA-III	5.443 ± 0.341	29.17 ± 2.86	4.420 ± 0.575	53.91 ± 3.67	23.236
MOEAD	24.08 ± 1.64	224.6 ± 14.2	32.32 ± 3.03	200.6 ± 12.9	120.400
SPEA2	4.615 ± 0.399	84.73 ± 6.30	4.215 ± 0.446	69.43 ± 4.52	40.748
SMS-EMOA	4.039 ± 0.474	33,972.1 ± 144.4	4.031 ± 0.518	51.63 ± 3.72	8507.950
MOWOA	125.5 ± 11.6	2131.4 ± 92.6	130.5 ± 16.0	2630.0 ± 159.4	1254.350
MORBMO	123.3 ± 10.8	2851.9 ± 72.3	128.2 ± 13.7	2532.1 ± 335.0	1408.875
MOWMA	57.74 ± 1.47	1536.4 ± 10.5	84.24 ± 2.19	934.9 ± 9.1	653.320
SMOWMA	88.23 ± 1.88	1518.9 ± 10.7	89.06 ± 1.11	914.4 ± 24.7	652.648

*Note: Values are reported as mean ± standard deviation. For each problem, every algorithm used the same objective-function-evaluation budget. The mean column is the arithmetic mean of the four problem-specific mean runtimes*.

**Table 11:** Normalized hypervolume results for welded beam design cases with different numbers of objectives.

Algorithm	2-Objective NHV	3-Objective NHV	4-Objective NHV	Mean Rank
MOEAD	0.874863 ± 0.036768	0.840232 ± 0.005766	0.745810 ± 0.003653	4.000
NSGA-II	0.977263 ± 0.003806	0.965783 ± 0.002235	0.954236 ± 0.002719	2.333
MOWOA	0.796487 ± 0.132698	0.685602 ± 0.167866	0.595920 ± 0.186457	5.000
MORBMO	0.977443 ± 0.002020	**0.969131 ± 0.001744**	0.953116 ± 0.003649	2.000
SMOWMA	**0.977788 ± 0.001978**	0.962525 ± 0.006216	**0.958652 ± 0.001868**	**1.667**

*Note: NHV denotes normalized hypervolume, for which a larger value is preferred. Values are reported as mean ± standard deviation over 30 independent runs. The best mean in each case and the lowest mean rank are shown in bold. Mean rank is descriptive across the three objective settings.*

**Table 12:** Holm-adjusted *p*-values for pairwise NHV comparisons between SMOWMA and the competing algorithms.

SMOWMA vs.	2-Objective	3-Objective	4-Objective
MOEAD	**1.1488 × 10^−10^ (+)**	**1.1488 × 10^−10^ (+)**	**1.1488 × 10^−10^ (+)**
NSGA-II	1.0000 (=)	0.0785 (=)	**2.1109 × 10^−8^ (+)**
MOWOA	**1.1488 × 10^−10^ (+)**	**1.1488 × 10^−10^ (+)**	**1.1488 × 10^−10^ (+)**
MORBMO	1.0000 (=)	**1.0203 × 10^−4^ (−)**	**2.1109 × 10^−8^ (+)**

*Note: Values are Holm-adjusted p-values obtained from two-sided Wilcoxon rank-sum tests on 30 independent runs. “+” indicates that SMOWMA performs significantly better, “−” indicates that it performs significantly worse, and “=” indicates that no significant difference is detected at α = 0.05.*

**Table 13:** Statistical results of SMOWMA for the four-bar truss problem over 30 independent runs.

Metric	Mean	Std.	Median	Best	Worst
GD ↓	1.6618 × 10^−3^	8.4059 × 10^−5^	1.6540 × 10^−3^	1.5301 × 10^−3^	1.8670 × 10^−3^
IGD ↓	3.0941 × 10^−3^	9.2409 × 10^−5^	3.0934 × 10^−3^	2.9708 × 10^−3^	3.3387 × 10^−3^
SP ↓	1.9975 × 10^−3^	1.6312 × 10^−4^	1.9970 × 10^−3^	1.6753 × 10^−3^	2.3939 × 10^−3^
NHV ↑	0.731312	0.000092	0.731317	0.731518	0.731107
Runtime (s) ↓	881.18	73.52	890.28	773.64	974.93

*Note: The symbols “↓” and “ ↑” indicate that a lower or higher value of the corresponding metric is preferred, respectively.*

**Table 14:** NHV results for the five-objective car side-impact design problem.

Algorithm	NHV (Mean ± Std)	Rank	Holm-Adjusted *p*-Value	Result
MOEAD	0.042455 ± 0.004073	5	1.1488 × 10^−10^	+
MOWOA	0.342689 ± 0.040142	4	1.8627 × 10^−9^	+
MORBMO	0.372931 ± 0.012000	3	3.4450 × 10^−10^	+
SMOWMA	0.403562 ± 0.006436	1	—	—
NSGA-II	0.384192 ± 0.010481	2	1.8627 × 10^−9^	+

*Note: “+” indicates that SMOWMA performs significantly better than the corresponding algorithm according to the Wilcoxon rank-sum test with Holm correction at α = 0.05. A higher NHV value is better.*

## Data Availability

The data that support the findings of this study will be made available from the corresponding author upon reasonable request. The source code of SMOWMA and the evaluation scripts (e.g., GD/IGD/HV/SP computation and convergence-curve plotting) are available on GitHub: https://github.com/Zadkl/SMOWMA.git (Accessed on 14 July 2026).

## Appendix A

**Table A1:** GD results for the benchmark problems.

	**ZDT1**		**ZDT2**		**ZDT3**	
Algorithms	Ave	Std	Ave	Std	Ave	Std
SMOWMA	4.8927 × 10^−3^	4.1798 × 10^−4^	4.9783 × 10^−4^	**2.0072 × 10^−5^**	**7.4292 × 10^−4^**	**3.0813 × 10^−5^**
MOSFOA	1.3315 × 10^−3^	6.7945 × 10^−4^	5.8567 × 10^−4^	5.4888 × 10^−4^	3.4691 × 10^−3^	2.7778 × 10^−3^
MOWOA	**3.9740 × 10^−4^**	**9.1810 × 10^−5^**	**4.8178 × 10^−4^**	3.5143 × 10^−5^	1.8496 × 10^−3^	6.8059 × 10^−4^
MORBMO	1.3174 × 10^−2^	3.4062 × 10^−3^	1.7073 × 10^−3^	4.1587 × 10^−4^	1.5996 × 10^−2^	2.8616 × 10^−2^
MOWMA	7.1000 × 10^−3^	2.0800 × 10^−4^	2.4294 × 10^−1^	1.4811 × 10^−2^	8.2178 × 10^−2^	1.7218 × 10^−2^
SMS-EMOA	5.8132 × 10^−3^	4.8077 × 10^−3^	1.7987 × 10^−2^	1.2692 × 10^−2^	3.1239 × 10^−3^	9.5284 × 10^−4^
MOEAD	7.5283 × 10^−2^	4.6073 × 10^−2^	5.4412 × 10^−4^	7.6331 × 10^−4^	1.5935 × 10^−1^	1.0419 × 10^−1^
SPEA2	9.9369 × 10^−3^	2.0525 × 10^−3^	2.7701 × 10^−2^	2.1335 × 10^−2^	5.5023 × 10^−3^	1.4715 × 10^−3^
NSGA-II	8.1823 × 10^−3^	1.7746 × 10^−3^	2.5384 × 10^−2^	1.2172 × 10^−2^	6.2336 × 10^−3^	4.8111 × 10^−3^
NSGA-III	8.8565 × 10^−3^	1.9060 × 10^−3^	1.9209 × 10^−2^	9.4849 × 10^−3^	9.0859 × 10^−3^	7.0601 × 10^−3^
	ZDT4		ZDT6		DTLZ1	
Algorithms	Ave	Std	Ave	Std	Ave	Std
SMOWMA	**1.4722 × 10^−3^**	**1.9882 × 10^−4^**	4.9908 × 10^−4^	**1.8946 × 10^−5^**	9.9062 × 10^−3^	4.5720 × 10^−4^
MOSFOA	1.8308 × 10^−3^	6.5519 × 10^−4^	1.3802 × 10^−3^	2.0769 × 10^−3^	6.7288 × 10^−1^	7.3458 × 10^−1^
MOWOA	1.6642 × 10^−1^	5.2226 × 10^−1^	**4.9808 × 10^−4^**	2.1549 × 10^−5^	2.5553 × 10^−1^	5.9085 × 10^−1^
MORBMO	2.5903 × 10^1^	1.6022 × 10^0^	1.2043 × 10^−1^	2.3213 × 10^−1^	2.6384 × 10^1^	1.0483 × 10^0^
MOWMA	1.1538 × 10^1^	1.6601 × 10^0^	8.0209 × 10^−1^	1.5357 × 10^−1^	2.4494 × 10^1^	9.0732 × 10^−1^
SMS-EMOA	3.2617 × 10^−1^	5.4111 × 10^−1^	1.4628 × 10^−2^	4.5784 × 10^−3^	**5.1509 × 10^−3^**	1.4260 × 10^−4^
MOEAD	5.3699 × 10^−2^	6.0667 × 10^−2^	6.1894 × 10^−3^	8.8745 × 10^−4^	5.2906 × 10^−3^	**1.0995 × 10^−4^**
SPEA2	3.5118 × 10^−1^	2.7153 × 10^−1^	1.9777 × 10^−2^	2.8624 × 10^−3^	5.6005 × 10^−3^	7.5013 × 10^−4^
NSGA-II	2.0039 × 10^−1^	2.0987 × 10^−1^	1.8808 × 10^−2^	6.3290 × 10^−3^	2.2303 × 10^−1^	6.8838 × 10^−1^
NSGA-III	1.2408 × 10^−1^	9.6921 × 10^−2^	2.5680 × 10^−2^	6.8178 × 10^−3^	1.0906 × 10^−2^	1.7989 × 10^−2^
	DTLZ2		DTLZ3		DTLZ4	
Algorithms	Ave	Std	Ave	Std	Ave	Std
SMOWMA	1.3696 × 10^−2^	1.0265 × 10^−3^	**1.1388 × 10^−2^**	**2.5070 × 10^−4^**	1.2641 × 10^−2^	5.1617 × 10^−4^
MOSFOA	3.2194 × 10^−2^	3.7136 × 10^−3^	1.8303 × 10^1^	1.4653 × 10^1^	4.0598 × 10^−2^	1.5951 × 10^−2^
MOWOA	2.1766 × 10^−2^	8.3220 × 10^−3^	1.0363 × 10^2^	1.4669 × 10^1^	1.8932 × 10^−2^	6.3746 × 10^−3^
MORBMO	2.8391 × 10^−2^	1.7658 × 10^−3^	1.8620 × 10^−2^	1.1338 × 10^−3^	3.1722 × 10^−2^	9.8602 × 10^−3^
MOWMA	1.7025 × 10^−2^	4.1410 × 10^−3^	1.4729 × 10^2^	3.5395 × 10^0^	1.2152 × 10^−2^	3.3277 × 10^−4^
SMS-EMOA	**1.1046 × 10^−2^**	2.2741 × 10^−4^	5.5358 × 10^−1^	4.5904 × 10^−1^	1.0687 × 10^−2^	1.9500 × 10^−4^
MOEAD	1.1839 × 10^−2^	3.1626 × 10^−3^	9.3098 × 10^−2^	4.7776 × 10^−2^	**1.0205 × 10^−2^**	1.7468 × 10^−3^
SPEA2	1.2213 × 10^−2^	3.8490 × 10^−4^	1.9349 × 10^−2^	2.0327 × 10^−2^	1.1938 × 10^−2^	3.8385 × 10^−4^
NSGA-II	1.1951 × 10^−2^	3.1320 × 10^−4^	1.5972 × 10^−2^	5.4285 × 10^−3^	1.2038 × 10^−2^	2.5461 × 10^−4^
NSGA-III	1.1842 × 10^−2^	**7.3989 × 10^−6^**	2.2519 × 10^−1^	5.0593 × 10^−1^	1.1845 × 10^−2^	**1.1475 × 10^−5^**
	DTLZ5		DTLZ6		WFG1	
Algorithms	Ave	Std	Ave	Std	Ave	Std
SMOWMA	**2.4548 × 10^−4^**	1.1126 × 10^−5^	2.2265 × 10^−4^	5.8326 × 10^−6^	**3.4602 × 10^−2^**	**3.3786 × 10^−4^**
MOSFOA	2.4014 × 10^−3^	6.3642 × 10^−4^	2.2887 × 10^−4^	6.5167 × 10^−6^	1.2910 × 10^−1^	4.0950 × 10^−2^
MOWOA	8.9062 × 10^−4^	2.6300 × 10^−4^	**2.2230 × 10^−4^**	7.2120 × 10^−6^	1.1755 × 10^0^	2.0796 × 10^−2^
MORBMO	1.2096 × 10^−3^	1.8293 × 10^−4^	4.1516 × 10^−2^	9.8720 × 10^−2^	1.1943 × 10^0^	2.2578 × 10^−2^
MOWMA	3.7652 × 10^−2^	3.6528 × 10^−3^	2.2301 × 10^−4^	**4.2543 × 10^−6^**	1.1182 × 10^0^	1.3639 × 10^−2^
SMS-EMOA	2.6197 × 10^−4^	7.9936 × 10^−6^	8.7368 × 10^−2^	3.1732 × 10^−2^	4.8632 × 10^−1^	1.8880 × 10^−1^
MOEAD	2.5505 × 10^−4^	**4.5133 × 10^−6^**	1.1026 × 10^−1^	5.8752 × 10^−2^	5.4631 × 10^−1^	1.9899 × 10^−1^
SPEA2	3.4531 × 10^−4^	1.3900 × 10^−4^	7.4530 × 10^−2^	3.3196 × 10^−2^	6.7797 × 10^−1^	2.1643 × 10^−1^
NSGA-II	3.9772 × 10^−4^	1.6736 × 10^−4^	6.8416 × 10^−2^	2.5864 × 10^−2^	5.2520 × 10^−1^	1.5146 × 10^−1^
NSGA-III	3.9247 × 10^−4^	3.6045 × 10^−4^	8.5799 × 10^−2^	2.8413 × 10^−2^	5.2648 × 10^−1^	1.2997 × 10^−1^
	WFG2		WFG3		WFG4	
Algorithms	Ave	Std	Ave	Std	Ave	Std
SMOWMA	2.6665 × 10^−3^	9.6533 × 10^−5^	6.2144 × 10^−3^	5.7392 × 10^−4^	1.6632 × 10^−1^	3.6800 × 10^−2^
MOSFOA	1.9063 × 10^−3^	**5.9445 × 10^−5^**	4.4071 × 10^−3^	2.4042 × 10^−4^	6.3277 × 10^−2^	1.7491 × 10^−2^
MOWOA	1.9162 × 10^−3^	6.6011 × 10^−5^	3.8694 × 10^−3^	3.2383 × 10^−4^	2.3913 × 10^−1^	5.6779 × 10^−3^
MORBMO	1.7270 × 10^−3^	9.7467 × 10^−5^	3.5320 × 10^−3^	1.9057 × 10^−4^	2.6144 × 10^−1^	4.0002 × 10^−3^
MOWMA	2.3351 × 10^−3^	8.5469 × 10^−5^	2.2956 × 10^−2^	2.1635 × 10^−3^	5.8890 × 10^−2^	1.4822 × 10^−2^
SMS-EMOA	**1.5918 × 10^−3^**	2.5128 × 10^−4^	**1.2180 × 10^−3^**	**1.1459 × 10^−4^**	**5.6582 × 10^−2^**	1.2910 × 10^−2^
MOEAD	3.5334 × 10^−3^	7.7140 × 10^−4^	4.8506 × 10^−3^	1.7536 × 10^−3^	2.1128 × 10^−1^	**2.8788 × 10^−3^**
SPEA2	1.7545 × 10^−3^	3.0515 × 10^−4^	1.6432 × 10^−3^	1.6469 × 10^−4^	7.3309 × 10^−2^	1.6350 × 10^−2^
NSGA-II	1.6585 × 10^−3^	1.9025 × 10^−4^	1.5830 × 10^−3^	2.6588 × 10^−4^	7.5946 × 10^−2^	1.8060 × 10^−2^
NSGA-III	1.7734 × 10^−3^	2.5707 × 10^−4^	1.8033 × 10^−3^	6.4650 × 10^−4^	8.4746 × 10^−2^	1.9760 × 10^−2^
	WFG5		WFG6		WFG7	
Algorithms	Ave	Std	Ave	Std	Ave	Std
SMOWMA	7.0718 × 10^−2^	2.6183 × 10^−3^	2.1799 × 10^−2^	**5.7425 × 10^−4^**	2.2969 × 10^−1^	3.2466 × 10^−3^
MOSFOA	**6.6504 × 10^−2^**	4.3036 × 10^−3^	**1.1640 × 10^−2^**	3.1794 × 10^−3^	4.7525 × 10^−2^	8.5549 × 10^−3^
MOWOA	2.8583 × 10^−1^	4.4883 × 10^−3^	2.2680 × 10^−1^	3.5948 × 10^−3^	2.2341 × 10^−1^	**1.1657 × 10^−3^**
MORBMO	2.9177 × 10^−1^	3.1953 × 10^−3^	2.2244 × 10^−1^	3.1452 × 10^−3^	2.2438 × 10^−1^	3.2994 × 10^−3^
MOWMA	6.8998 × 10^−2^	**1.1111 × 10^−3^**	2.2199 × 10^−2^	9.3758 × 10^−4^	4.7415 × 10^−2^	6.4089 × 10^−3^
SMS-EMOA	6.9672 × 10^−2^	3.2594 × 10^−3^	9.5255 × 10^−2^	4.2168 × 10^−2^	**3.7866 × 10^−2^**	6.2722 × 10^−3^
MOEAD	2.6665 × 10^−1^	3.8781 × 10^−3^	3.3680 × 10^−1^	3.8877 × 10^−2^	2.0973 × 10^−1^	2.8270 × 10^−3^
SPEA2	6.7115 × 10^−2^	1.6654 × 10^−3^	1.0514 × 10^−1^	3.9727 × 10^−2^	4.8667 × 10^−2^	5.7036 × 10^−3^
NSGA-II	6.9896 × 10^−2^	2.4319 × 10^−3^	9.6138 × 10^−2^	3.1680 × 10^−2^	4.6364 × 10^−2^	7.8094 × 10^−3^
NSGA-III	7.1458 × 10^−2^	4.4996 × 10^−3^	1.2527 × 10^−1^	3.1038 × 10^−2^	6.3410 × 10^−2^	4.8207 × 10^−3^
	WFG8		WFG9		UF1	
Algorithms	Ave	Std	Ave	Std	Ave	Std
SMOWMA	1.8010 × 10^−1^	3.1702 × 10^−2^	6.5246 × 10^−3^	2.5291 × 10^−3^	2.5465 × 10^−2^	8.4949 × 10^−3^
MOSFOA	**2.6593 × 10^−2^**	3.4203 × 10^−2^	4.6264 × 10^−3^	**9.1069 × 10^−4^**	3.0125 × 10^−2^	1.5305 × 10^−2^
MOWOA	2.6446 × 10^−1^	**1.9130 × 10^−2^**	8.5048 × 10^−3^	2.3629 × 10^−3^	3.6346 × 10^−2^	1.3838 × 10^−2^
MORBMO	2.7993 × 10^−1^	1.9158 × 10^−2^	8.6481 × 10^−3^	1.3680 × 10^−3^	**2.8619 × 10^−3^**	**2.1747 × 10^−3^**
MOWMA	9.6561 × 10^−2^	3.0337 × 10^−2^	9.5641 × 10^−3^	9.9485 × 10^−3^	1.1297 × 10^−1^	1.9521 × 10^−2^
SMS-EMOA	1.3397 × 10^−1^	5.9196 × 10^−2^	**4.5115 × 10^−3^**	1.8606 × 10^−3^	6.0671 × 10^−3^	1.0508 × 10^−2^
MOEAD	2.0627 × 10^−1^	5.7474 × 10^−2^	1.6916 × 10^−2^	1.3864 × 10^−2^	3.9115 × 10^−2^	5.9398 × 10^−2^
SPEA2	1.4770 × 10^−1^	7.8601 × 10^−2^	5.8214 × 10^−3^	1.6157 × 10^−3^	6.1147 × 10^−3^	6.6920 × 10^−3^
NSGA-II	8.8964 × 10^−2^	4.8031 × 10^−2^	4.8925 × 10^−3^	2.2374 × 10^−3^	8.7517 × 10^−3^	1.2944 × 10^−2^
NSGA-III	1.5073 × 10^−1^	8.1677 × 10^−2^	6.1205 × 10^−3^	3.4574 × 10^−3^	3.5843 × 10^−3^	2.7956 × 10^−3^
	UF2		UF3		UF4	
Algorithms	Ave	Std	Ave	Std	Ave	Std
SMOWMA	2.4859 × 10^−2^	9.7981 × 10^−3^	2.6966 × 10^−1^	3.7182 × 10^−2^	1.9861 × 10^−2^	1.0311 × 10^−2^
MOSFOA	4.4053 × 10^−2^	3.5233 × 10^−2^	1.4113 × 10^−1^	2.3298 × 10^−2^	3.0308 × 10^−2^	1.1781 × 10^−2^
MOWOA	1.1366 × 10^−1^	5.4235 × 10^−2^	1.5828 × 10^−1^	3.1701 × 10^−2^	9.9932 × 10^−3^	7.5309 × 10^−3^
MORBMO	1.8492 × 10^−2^	4.6014 × 10^−3^	**3.0872 × 10^−2^**	1.6904 × 10^−2^	1.1737 × 10^−2^	1.5413 × 10^−2^
MOWMA	1.0033 × 10^−1^	1.1809 × 10^−2^	9.9536 × 10^−1^	3.3033 × 10^−1^	1.2527 × 10^−1^	4.5872 × 10^−2^
SMS-EMOA	2.3710 × 10^−2^	9.9216 × 10^−3^	3.9021 × 10^−2^	2.0580 × 10^−2^	3.9858 × 10^−2^	9.4710 × 10^−2^
MOEAD	**1.0931 × 10^−2^**	6.9692 × 10^−3^	4.5703 × 10^−2^	2.3099 × 10^−2^	3.4100 × 10^−2^	2.7041 × 10^−2^
SPEA2	1.6515 × 10^−2^	3.5424 × 10^−3^	3.6166 × 10^−2^	**1.5225 × 10^−2^**	1.8925 × 10^−2^	2.2002 × 10^−2^
NSGA-II	1.5894 × 10^−2^	**2.7366 × 10^−3^**	8.5201 × 10^−2^	4.2884 × 10^−2^	**5.6306 × 10^−3^**	**7.1889 × 10^−3^**
NSGA-III	2.5317 × 10^−2^	4.5976 × 10^−3^	5.4790 × 10^−2^	3.7020 × 10^−2^	1.2106 × 10^−1^	1.7184 × 10^−1^
	UF5		UF6		UF7	
Algorithms	Ave	Std	Ave	Std	Ave	Std
SMOWMA	**1.8068 × 10^−1^**	**4.9257 × 10^−2^**	1.1770 × 10^−1^	**2.2019 × 10^−2^**	1.3809 × 10^−2^	7.8886 × 10^−3^
MOSFOA	2.6756 × 10^−1^	1.3791 × 10^−1^	2.1429 × 10^−1^	5.8082 × 10^−2^	1.8416 × 10^−2^	9.7496 × 10^−3^
MOWOA	5.7518 × 10^−1^	2.4223 × 10^−1^	2.2710 × 10^−1^	1.2120 × 10^−1^	3.0726 × 10^−2^	1.6471 × 10^−2^
MORBMO	4.0615 × 10^−1^	1.8434 × 10^−1^	**7.0423 × 10^−2^**	3.5032 × 10^−2^	7.4787 × 10^−3^	6.5318 × 10^−3^
MOWMA	1.8760 × 10^0^	1.3496 × 10^−1^	7.4059 × 10^−1^	6.9955 × 10^−2^	1.0785 × 10^−1^	2.8491 × 10^−2^
SMS-EMOA	3.1148 × 10^−1^	1.2830 × 10^−1^	1.1048 × 10^−1^	7.3513 × 10^−2^	3.5673 × 10^−2^	7.2263 × 10^−2^
MOEAD	6.6612 × 10^−1^	1.8641 × 10^−1^	1.5346 × 10^−1^	1.2676 × 10^−1^	1.1726 × 10^−2^	1.5522 × 10^−2^
SPEA2	4.1465 × 10^−1^	1.0193 × 10^−1^	9.6918 × 10^−2^	5.7414 × 10^−2^	**7.0483 × 10^−3^**	**5.5491 × 10^−3^**
NSGA-II	4.7588 × 10^−1^	2.3899 × 10^−1^	1.2191 × 10^−1^	7.1619 × 10^−2^	2.2069 × 10^−2^	4.1269 × 10^−2^
NSGA-III	2.6290 × 10^−1^	9.1261 × 10^−2^	8.9503 × 10^−2^	4.6654 × 10^−2^	3.4546 × 10^−2^	6.1912 × 10^−2^
	CF1		CF2		CF3	
Algorithms	Ave	Std	Ave	Std	Ave	Std
SMOWMA	4.0235 × 10^−2^	1.0073 × 10^−3^	**1.5503 × 10^−1^**	9.1120 × 10^−3^	3.0706 × 10^−1^	8.2807 × 10^−2^
MOSFOA	9.4971 × 10^−2^	1.7533 × 10^−2^	1.9679 × 10^−1^	**8.8685 × 10^−3^**	3.3534 × 10^−1^	1.1190 × 10^−1^
MOWOA	1.9692 × 10^−2^	1.2471 × 10^−2^	2.1874 × 10^−1^	2.6246 × 10^−2^	8.6607 × 10^−1^	4.7010 × 10^−1^
MORBMO	4.7608 × 10^−2^	2.4161 × 10^−2^	2.2257 × 10^−1^	1.2716 × 10^−2^	3.6282 × 10^−1^	1.0951 × 10^−1^
MOWMA	**1.9221 × 10^−2^**	2.2973 × 10^−3^	4.1759 × 10^−1^	5.8312 × 10^−2^	1.7965 × 10^0^	1.5224 × 10^−1^
SMS-EMOA	2.0372 × 10^−2^	8.9344 × 10^−5^	1.9208 × 10^−1^	1.7897 × 10^−2^	**2.9031 × 10^−1^**	**6.0111 × 10^−2^**
MOEAD	2.0252 × 10^−2^	6.7304 × 10^−3^	2.7227 × 10^0^	2.2869 × 10^−2^	5.1490 × 10^−1^	1.5465 × 10^−1^
SPEA2	2.0401 × 10^−2^	3.0591 × 10^−4^	8.8765 × 10^−1^	4.1354 × 10^−2^	3.8076 × 10^−1^	7.7136 × 10^−2^
NSGA-II	2.0410 × 10^−2^	2.7310 × 10^−4^	4.2387 × 10^−1^	8.8656 × 10^−2^	3.2665 × 10^−1^	6.0637 × 10^−2^
NSGA-III	2.0361 × 10^−2^	**3.5223 × 10^−7^**	2.7341 × 10^−1^	3.6961 × 10^−2^	4.5358 × 10^−1^	1.7441 × 10^−1^
	CF4		CF5		CF6	
Algorithms	Ave	Std	Ave	Std	Ave	Std
SMOWMA	**1.8702 × 10^−2^**	**3.6814 × 10^−3^**	3.7189 × 10^−1^	2.3687 × 10^−1^	**6.7130 × 10^−2^**	**1.3455 × 10^−3^**
MOSFOA	4.0660 × 10^−2^	6.5652 × 10^−3^	3.6311 × 10^−1^	2.1472 × 10^−1^	2.8975 × 10^−1^	1.2844 × 10^−2^
MOWOA	5.8542 × 10^−2^	1.9260 × 10^−2^	**1.6467 × 10^−1^**	1.8824 × 10^−1^	3.1965 × 10^−1^	5.9491 × 10^−2^
MORBMO	3.6142 × 10^−2^	3.9024 × 10^−3^	4.4850 × 10^−1^	1.8336 × 10^−1^	2.8235 × 10^−1^	7.9105 × 10^−3^
MOWMA	1.1921 × 10^−1^	1.0997 × 10^−2^	2.3191 × 10^0^	5.3456 × 10^−1^	3.5220 × 10^−1^	4.0705 × 10^−2^
SMS-EMOA	2.6770 × 10^−2^	7.8334 × 10^−3^	4.1750 × 10^−1^	3.1048 × 10^−1^	2.7456 × 10^−1^	7.5398 × 10^−3^
MOEAD	7.2983 × 10^−2^	1.0608 × 10^−2^	4.9980 × 10^−1^	**1.0450 × 10^−1^**	2.9458 × 10^−1^	7.0345 × 10^−3^
SPEA2	3.6391 × 10^−2^	1.1247 × 10^−2^	3.2233 × 10^−1^	1.4826 × 10^−1^	2.7701 × 10^−1^	6.0025 × 10^−3^
NSGA-II	2.9632 × 10^−2^	7.0623 × 10^−3^	3.1548 × 10^−1^	1.4192 × 10^−1^	2.8581 × 10^−1^	8.7113 × 10^−3^
NSGA-III	5.1929 × 10^−2^	1.0162 × 10^−2^	4.8549 × 10^−1^	1.9485 × 10^−1^	2.8383 × 10^−1^	1.8661 × 10^−2^
	CF7		CF8		CF9	
Algorithms	Ave	Std	Ave	Std	Ave	Std
SMOWMA	**5.9688 × 10^−1^**	**1.1447 × 10^−1^**	4.6082 × 10^−1^	**4.7738 × 10^−3^**	2.9281 × 10^−1^	4.5810 × 10^−2^
MOSFOA	7.8958 × 10^−1^	1.5781 × 10^−1^	6.9319 × 10^0^	3.4369 × 10^0^	2.1880 × 10^0^	3.1058 × 10^−1^
MOWOA	1.4351 × 10^0^	6.7156 × 10^−1^	3.6707 × 10^1^	5.4153 × 10^0^	6.8699 × 10^−1^	3.5073 × 10^−2^
MORBMO	7.2167 × 10^−1^	3.7554 × 10^−1^	3.9552 × 10^1^	4.8438 × 10^1^	2.0860 × 10^−1^	5.9376 × 10^−2^
MOWMA	2.1938 × 10^0^	2.9251 × 10^−1^	7.6808 × 10^1^	2.5558 × 10^1^	4.9206 × 10^−1^	3.9451 × 10^−2^
SMS-EMOA	8.3368 × 10^−1^	3.0942 × 10^−1^	3.2856 × 10^0^	3.2564 × 10^0^	**1.1917 × 10^−2^**	**3.8066 × 10^−4^**
MOEAD	7.3099 × 10^−1^	2.2957 × 10^−1^	**4.4601 × 10^−1^**	1.4389 × 10^−2^	2.6237 × 10^−2^	1.4879 × 10^−2^
SPEA2	6.7002 × 10^−1^	1.7412 × 10^−1^	5.5367 × 10^1^	9.7157 × 10^1^	3.5311 × 10^−2^	3.6547 × 10^−2^
NSGA-II	7.3032 × 10^−1^	2.1728 × 10^−1^	7.3687 × 10^1^	6.2507 × 10^1^	2.0620 × 10^−1^	4.4184 × 10^−2^
NSGA-III	6.4906 × 10^−1^	2.0464 × 10^−1^	5.5866 × 10^0^	1.1296 × 10^1^	1.0897 × 10^−1^	3.5581 × 10^−2^
	CF10					
Algorithms	Ave	Std				
SMOWMA	3.4067 × 10^−2^	4.9733 × 10^−3^				
MOSFOA	3.9741 × 10^−2^	6.8423 × 10^−3^				
MOWOA	**3.1958 × 10^−2^**	**1.5175 × 10^−3^**				
MORBMO	3.2771 × 10^−2^	5.0321 × 10^−3^				
MOWMA	4.7914 × 10^−2^	3.8420 × 10^−3^				
SMS-EMOA	3.7645 × 10^−2^	3.9640 × 10^−3^				
MOEAD	5.3406 × 10^−2^	8.1048 × 10^−3^				
SPEA2	4.3895 × 10^−2^	9.1211 × 10^−3^				
NSGA-II	4.7914 × 10^−2^	1.6243 × 10^−3^				
NSGA-III	6.6405 × 10^−2^	1.5467 × 10^−2^				

**Table A2:** IGD results for the benchmark problems.

	**ZDT1**		**ZDT2**		**ZDT3**	
Algorithms	Ave	Std	Ave	Std	Ave	Std
SMOWMA	**5.5652 × 10^−3^**	4.0009 × 10^−4^	**2.9808 × 10^−3^**	**1.1831 × 10^−4^**	**2.8025 × 10^−3^**	**5.2549 × 10^−5^**
MOSFOA	3.8977 × 10^−2^	1.5133 × 10^−1^	2.9435 × 10^−1^	3.7548 × 10^−1^	2.9193 × 10^−2^	3.4294 × 10^−3^
MOWOA	2.1474 × 10^−2^	1.4039 × 10^−3^	8.1667 × 10^−3^	6.1836 × 10^−4^	2.7860 × 10^−2^	9.1738 × 10^−4^
MORBMO	1.6228 × 10^−2^	3.0539 × 10^−3^	5.2861 × 10^−3^	3.0525 × 10^−4^	2.5225 × 10^−2^	3.2139 × 10^−2^
MOWMA	7.5720 × 10^−3^	**2.1300 × 10^−4^**	2.4538 × 10^−1^	1.7357 × 10^−2^	2.5791 × 10^−1^	7.3141 × 10^−3^
SMS-EMOA	1.1610 × 10^−2^	8.1765 × 10^−4^	3.3619 × 10^−1^	3.0038 × 10^−1^	2.4110 × 10^−2^	2.2606 × 10^−2^
MOEAD	1.7681 × 10^−1^	7.6682 × 10^−2^	5.7480 × 10^−1^	2.0399 × 10^−1^	1.4988 × 10^−1^	8.5541 × 10^−2^
SPEA2	1.5325 × 10^−2^	1.6297 × 10^−3^	7.1153 × 10^−2^	1.2581 × 10^−1^	2.5876 × 10^−2^	2.2155 × 10^−2^
NSGA-II	1.4705 × 10^−2^	1.4952 × 10^−3^	8.3153 × 10^−2^	1.3344 × 10^−1^	1.6892 × 10^−2^	5.2042 × 10^−3^
NSGA-III	2.5206 × 10^−2^	2.1646 × 10^−2^	2.8641 × 10^−1^	2.7796 × 10^−1^	3.2368 × 10^−2^	2.3881 × 10^−2^
	ZDT4		ZDT6		DTLZ1	
Algorithms	Ave	Std	Ave	Std	Ave	Std
SMOWMA	**2.9587 × 10^−3^**	**1.3482 × 10^−4^**	9.1447 × 10^−2^	**2.8604 × 10^−6^**	2.1606 × 10^−2^	6.2267 × 10^−4^
MOSFOA	9.4134 × 10^−2^	2.8771 × 10^−1^	9.0524 × 10^−^	1.8004 × 10^−3^	5.4696 × 10^−1^	5.7418 × 10^−1^
MOWOA	1.5752 × 10^−1^	4.7397 × 10^−1^	9.1583 × 10^−2^	3.9592 × 10^−5^	2.2123 × 10^−1^	4.6721 × 10^−1^
MORBMO	1.2112 × 10^1^	2.2529 × 10^0^	9.1274 × 10^−2^	6.1290 × 10^−4^	9.6055 × 10^0^	2.2949 × 10^0^
MOWMA	7.3821 × 10^0^	1.6753 × 10^0^	9.2950 × 10^−2^	1.0946 × 10^−3^	1.2370 × 10^1^	2.1976 × 10^0^
SMS-EMOA	3.8449 × 10^−1^	4.4528 × 10^−1^	**8.9784 × 10^−2^**	2.3337 × 10^−4^	1.4701 × 10^−2^	4.0700 × 10^−5^
MOEAD	5.7107 × 10^−2^	6.1158 × 10^−2^	9.0954 × 10^−2^	1.1115 × 10^−4^	1.4574 × 10^−2^	6.2872 × 10^−5^
SPEA2	2.8281 × 10^−1^	1.8324 × 10^−1^	9.1550 × 10^−2^	3.5347 × 10^−3^	1.5778 × 10^−2^	1.3838 × 10^−4^
NSGA-II	2.2292 × 10^−1^	1.7905 × 10^−1^	9.0283 × 10^−2^	5.2888 × 10^−4^	2.4149 × 10^−2^	2.3100 × 10^−3^
NSGA-III	2.9389 × 10^−1^	1.2696 × 10^−1^	9.0643 × 10^−2^	1.0800 × 10^−3^	**1.4475 × 10^−2^**	**2.0327 × 10^−5^**
	DTLZ2		DTLZ3		DTLZ4	
Algorithms	Ave	Std	Ave	Std	Ave	Std
SMOWMA	5.5550 × 10^−2^	2.2762 × 10^−3^	4.6743 × 10^−2^	**8.4105 × 10^−4^**	5.4705 × 10^−2^	1.4351 × 10^−3^
MOSFOA	5.7273 × 10^−2^	2.5947 × 10^−3^	1.7968 × 10^1^	1.4395 × 10^1^	5.8972 × 10^−2^	2.5183 × 10^−3^
MOWOA	5.9451 × 10^−2^	3.4978 × 10^−3^	9.6078 × 10^1^	1.2374 × 10^1^	5.5545 × 10^−2^	3.7683 × 10^−3^
MORBMO	5.7282 × 10^−2^	1.5557 × 10^−3^	4.9311 × 10^−2^	9.0337 × 10^−4^	5.6254 × 10^−2^	1.5455 × 10^−3^
MOWMA	5.6157 × 10^−2^	3.9511 × 10^−3^	5.0848 × 10^−2^	1.5843 × 10^−3^	5.1038 × 10^−2^	1.2488 × 10^−3^
SMS-EMOA	6.0116 × 10^−2^	7.1900 × 10^−4^	5.5564 × 10^−2^	3.3899 × 10^−3^	6.0440 × 10^−2^	7.6468 × 10^−4^
MOEAD	3.9432 × 10^−2^	**5.2235 × 10^−7^**	7.1071 × 10^−2^	1.8783 × 10^−2^	3.8923 × 10^−1^	3.8489 × 10^−1^
SPEA2	4.2465 × 10^−2^	1.4801 × 10^−3^	4.5886 × 10^−2^	5.0242 × 10^−3^	4.2311 × 10^−2^	1.0845 × 10^−3^
NSGA-II	5.4285 × 10^−2^	2.0803 × 10^−3^	4.8292 × 10^−2^	1.6268 × 10^−3^	5.2281 × 10^−2^	2.3113 × 10^−3^
NSGA-III	**3.9357 × 10^−2^**	1.9023 × 10^−6^	**4.4239 × 10^−2^**	1.3459 × 10^−3^	**3.9357 × 10^−2^**	**2.2372 × 10^−6^**
	DTLZ5		DTLZ6		WFG1	
Algorithms	Ave	Std	Ave	Std	Ave	Std
SMOWMA	3.6819 × 10^−3^	1.5064 × 10^−4^	3.5210 × 10^−3^	1.6087 × 10^−4^	3.6237 × 10^−1^	**6.5551 × 10^−5^**
MOSFOA	4.0096 × 10^−3^	2.3432 × 10^−4^	4.0572 × 10^−3^	6.1499 × 10^−4^	**1.2775 × 10^−1^**	3.8590 × 10^−2^
MOWOA	3.6503 × 10^−3^	1.9876 × 10^−4^	3.5912 × 10^−3^	2.0459 × 10^−4^	1.1589 × 10^0^	2.1862 × 10^−2^
MORBMO	3.8091 × 10^−3^	2.1719 × 10^−4^	1.9899 × 10^−2^	3.4104 × 10^−2^	1.1422 × 10^0^	2.3416 × 10^−2^
MOWMA	2.7462 × 10^−2^	2.0898 × 10^−3^	**2.9160 × 10^−3^**	**1.2231 × 10^−4^**	1.1073 × 10^0^	1.2361 × 10^−2^
SMS-EMOA	3.7400 × 10^−3^	2.2498 × 10^−4^	8.6852 × 10^−2^	3.1249 × 10^−2^	1.4129 × 10^0^	4.0785 × 10^−2^
MOEAD	2.7918 × 10^−2^	**1.6356 × 10^−6^**	7.8867 × 10^−2^	2.1261 × 10^−2^	9.0261 × 10^−1^	2.1178 × 10^−1^
SPEA2	**2.8764 × 10^−3^**	1.1276 × 10^−4^	6.9428 × 10^−2^	3.0694 × 10^−2^	1.1169 × 10^0^	1.4282 × 10^−1^
NSGA-II	3.4767 × 10^−3^	1.6199 × 10^−4^	6.0403 × 10^−2^	2.5928 × 10^−2^	1.1446 × 10^0^	9.1814 × 10^−2^
NSGA-III	3.1724 × 10^−2^	1.6207 × 10^−4^	8.1982 × 10^−2^	2.3361 × 10^−2^	1.2658 × 10^0^	8.8889 × 10^−2^
	WFG2		WFG3		WFG4	
Algorithms	Ave	Std	Ave	Std	Ave	Std
SMOWMA	9.9848 × 10^−3^	1.2974 × 10^−3^	1.0465 × 10^−2^	7.3983 × 10^−4^	1.5828 × 10^−1^	3.4809 × 10^−2^
MOSFOA	5.7410 × 10^−3^	1.4152 × 10^−4^	8.3226 × 10^−3^	2.1770 × 10^−4^	**9.9952 × 10^−3^**	1.1233 × 10^−3^
MOWOA	**5.5901 × 10^−3^**	**1.1712 × 10^−4^**	8.0064 × 10^−3^	2.1726 × 10^−4^	2.2492 × 10^−1^	4.2406 × 10^−3^
MORBMO	5.8216 × 10^−3^	2.8340 × 10^−4^	7.9426 × 10^−3^	1.5975 × 10^−4^	2.3005 × 10^−1^	1.2885 × 10^−3^
MOWMA	7.8601 × 10^−3^	3.2336 × 10^−3^	1.4291 × 10^−2^	2.9132 × 10^−4^	3.5407 × 10^−2^	2.9773 × 10^−3^
SMS-EMOA	4.4393 × 10^−1^	2.4686 × 10^−2^	**6.4800 × 10^−3^**	**9.7092 × 10^−5^**	3.1381 × 10^−2^	8.1776 × 10^−3^
MOEAD	7.5850 × 10^−2^	9.6824 × 10^−3^	1.0433 × 10^−2^	4.0238 × 10^−3^	2.1310 × 10^−1^	**7.2964 × 10^−4^**
SPEA2	9.4297 × 10^−2^	2.4543 × 10^−2^	7.3123 × 10^−3^	2.4204 × 10^−4^	3.5798 × 10^−2^	1.3894 × 10^−2^
NSGA-II	7.6251 × 10^−2^	1.2859 × 10^−4^	7.1034 × 10^−3^	1.8258 × 10^−4^	2.6775 × 10^−2^	6.4626 × 10^−3^
NSGA-III	8.3799 × 10^−2^	2.5239 × 10^−4^	6.9045 × 10^−3^	2.9268 × 10^−4^	2.4812 × 10^−2^	7.7342 × 10^−3^
	WFG5		WFG6		WFG7	
Algorithms	Ave	Std	Ave	Std	Ave	Std
SMOWMA	6.7738 × 10^−2^	**4.2363 × 10^−5^**	1.2229 × 10^−2^	3.6311 × 10^−4^	2.1198 × 10^−1^	3.5441 × 10^−4^
MOSFOA	**6.3453 × 10^−2^**	3.2361 × 10^−3^	**1.0327 × 10^−2^**	4.5276 × 10^−4^	**9.4973 × 10^−3^**	8.2910 × 10^−4^
MOWOA	2.7069 × 10^−1^	2.6723 × 10^−4^	2.1303 × 10^−1^	4.7780 × 10^−4^	2.1393 × 10^−1^	3.4027 × 10^−4^
MORBMO	2.6961 × 10^−1^	5.8785 × 10^−4^	2.1203 × 10^−1^	**3.3442 × 10^−4^**	2.1224 × 10^−1^	**2.9630 × 10^−4^**
MOWMA	6.5195 × 10^−2^	3.3354 × 10^−4^	1.7065 × 10^−2^	3.0922 × 10^−3^	1.3635 × 10^−2^	8.6257 × 10^−4^
SMS-EMOA	6.8551 × 10^−2^	1.8619 × 10^−3^	1.0003 × 10^−1^	3.7423 × 10^−2^	3.5698 × 10^−2^	8.6999 × 10^−3^
MOEAD	2.6994 × 10^−1^	3.9858 × 10^−3^	3.6450 × 10^−1^	6.7368 × 10^−2^	2.1485 × 10^−1^	9.1236 × 10^−3^
SPEA2	6.8476 × 10^−2^	1.3270 × 10^−3^	1.1140 × 10^−1^	3.8647 × 10^−2^	5.1528 × 10^−2^	1.0370 × 10^−2^
NSGA-II	6.8331 × 10^−2^	6.7473 × 10^−4^	9.9845 × 10^−2^	2.9717 × 10^−2^	3.3503 × 10^−2^	1.8631 × 10^−2^
NSGA-III	6.6262 × 10^−2^	4.2811 × 10^−4^	1.2804 × 10^−1^	3.0972 × 10^−2^	3.2764 × 10^−2^	1.5010 × 10^−2^
	WFG8		WFG9		UF1	
Algorithms	Ave	Std	Ave	Std	Ave	Std
SMOWMA	1.9349 × 10^−1^	3.3425 × 10^−2^	1.4518 × 10^−2^	1.6245 × 10^−3^	7.3712 × 10^−2^	4.4102 × 10^−3^
MOSFOA	**4.4244 × 10^−2^**	2.8684 × 10^−2^	1.2246 × 10^−2^	**7.5802 × 10^−4^**	**3.8963 × 10^−2^**	2.4116 × 10^−2^
MOWOA	2.7204 × 10^−1^	9.6540 × 10^−3^	1.5012 × 10^−2^	1.6449 × 10^−3^	8.8346 × 10^−2^	2.4063 × 10^−2^
MORBMO	2.8168 × 10^−1^	**9.1867 × 10^−3^**	1.5242 × 10^−2^	1.1330 × 10^−3^	6.9414 × 10^−2^	7.5505 × 10^−3^
MOWMA	2.2128 × 10^−1^	1.3185 × 10^−1^	1.5048 × 10^−2^	8.4084 × 10^−3^	1.1763 × 10^−1^	**3.3620 × 10^−3^**
SMS-EMOA	1.4838 × 10^−1^	5.3396 × 10^−2^	**1.2009 × 10^−2^**	1.4681 × 10^−3^	1.2718 × 10^−1^	1.3359 × 10^−2^
MOEAD	2.2136 × 10^−1^	5.2640 × 10^−2^	3.1196 × 10^−2^	2.1021 × 10^−2^	2.5363 × 10^−1^	7.4786 × 10^−2^
SPEA2	1.5555 × 10^−1^	7.5097 × 10^−2^	1.3575 × 10^−2^	1.1576 × 10^−3^	1.1986 × 10^−1^	2.0005 × 10^−2^
NSGA-II	9.7517 × 10^−2^	4.1409 × 10^−2^	1.2431 × 10^−2^	1.5402 × 10^−3^	1.2085 × 10^−1^	2.5945 × 10^−2^
NSGA-III	1.6935 × 10^−1^	8.3081 × 10^−2^	1.3413 × 10^−2^	2.6336 × 10^−3^	1.3841 × 10^−1^	2.5758 × 10^−2^
	UF2		UF3		UF4	
Algorithms	Ave	Std	Ave	Std	Ave	Std
SMOWMA	3.1353 × 10^−2^	2.1641 × 10^−3^	3.0117 × 10^−1^	**1.0794 × 10^−2^**	9.7300 × 10^−2^	7.7574 × 10^−3^
MOSFOA	**1.6807 × 10^−2^**	1.5687 × 10^−3^	1.7149 × 10^−1^	4.1050 × 10^−2^	**3.3424 × 10^−2^**	1.9545 × 10^−2^
MOWOA	5.5101 × 10^−2^	5.2306 × 10^−3^	**1.5458 × 10^−1^**	3.1687 × 10^−2^	1.2962 × 10^−1^	1.0612 × 10^−2^
MORBMO	2.4767 × 10^−2^	**1.4879 × 10^−3^**	3.1004 × 10^−1^	1.1152 × 10^−2^	8.4534 × 10^−2^	1.0702 × 10^−2^
MOWMA	8.6457 × 10^−2^	2.3026 × 10^−3^	5.7006 × 10^−1^	2.5499 × 10^−2^	1.0647 × 10^−1^	**3.8229 × 10^−3^**
SMS-EMOA	6.1488 × 10^−2^	1.2619 × 10^−2^	4.5310 × 10^−1^	8.9485 × 10^−2^	1.4969 × 10^−1^	4.1612 × 10^−2^
MOEAD	2.5682 × 10^−1^	1.0540 × 10^−1^	5.5470 × 10^−1^	8.9211 × 10^−2^	2.5180 × 10^−1^	1.1666 × 10^−1^
SPEA2	4.8605 × 10^−2^	1.0352 × 10^−2^	3.8238 × 10^−1^	9.9629 × 10^−2^	1.8238 × 10^−1^	6.8390 × 10^−2^
NSGA-II	4.9524 × 10^−2^	8.0377 × 10^−3^	3.0857 × 10^−1^	1.1288 × 10^−1^	1.5979 × 10^−1^	5.0534 × 10^−2^
NSGA-III	5.2012 × 10^−2^	6.3906 × 10^−3^	4.5478 × 10^−1^	9.7696 × 10^−2^	1.4351 × 10^−1^	3.5821 × 10^−2^
	UF5		UF6		UF7	
Algorithms	Ave	Std	Ave	Std	Ave	Std
SMOWMA	**2.1200 × 10^−1^**	**2.9714 × 10^−2^**	1.5404 × 10^−1^	**6.2526 × 10^−3^**	4.2581 × 10^−2^	4.0414 × 10^−3^
MOSFOA	2.6301 × 10^−1^	1.8709 × 10^−1^	**1.4550 × 10^−1^**	3.3760 × 10^−2^	**1.8976 × 10^−2^**	1.1154 × 10^−2^
MOWOA	8.4776 × 10^−1^	8.8544 × 10^−2^	2.4775 × 10^−1^	7.1305 × 10^−2^	7.0972 × 10^−2^	1.2357 × 10^−2^
MORBMO	1.1315 × 10^0^	7.8246 × 10^−2^	3.0945 × 10^−1^	3.5450 × 10^−2^	3.3098 × 10^−2^	3.3068 × 10^−3^
MOWMA	1.5099 × 10^0^	7.3871 × 10^−2^	5.9526 × 10^−1^	1.5403 × 10^−2^	9.5200 × 10^−2^	**2.2804 × 10^−3^**
SMS-EMOA	4.5395 × 10^−1^	1.4266 × 10^−1^	2.7856 × 10^−1^	1.0508 × 10^−1^	2.0418 × 10^−1^	1.6628 × 10^−1^
MOEAD	8.6940 × 10^−1^	2.1401 × 10^−1^	5.0811 × 10^−1^	1.9195 × 10^−1^	5.7807 × 10^−1^	2.1720 × 10^−1^
SPEA2	4.8355 × 10^−1^	9.7307 × 10^−2^	2.3010 × 10^−1^	4.2974 × 10^−2^	1.3725 × 10^−1^	1.0777 × 10^−1^
NSGA-II	5.0248 × 10^−1^	1.3545 × 10^−1^	2.5677 × 10^−1^	7.0840 × 10^−2^	7.8165 × 10^−2^	2.4146 × 10^−2^
NSGA-III	3.4597 × 10^−1^	4.8976 × 10^−2^	2.3555 × 10^−1^	4.1603 × 10^−2^	2.5779 × 10^−1^	1.9638 × 10^−1^
	CF1		CF2		CF3	
Algorithms	Ave	Std	Ave	Std	Ave	Std
SMOWMA	2.2250 × 10^−3^	1.0494 × 10^−4^	1.6737 × 10^−1^	1.8935 × 10^−3^	**2.2478 × 10^−1^**	**3.4673 × 10^−2^**
MOSFOA	1.3654 × 10^−1^	6.7250 × 10^−4^	1.5111 × 10^−1^	**1.3520 × 10^−3^**	2.3252 × 10^−1^	3.8194 × 10^−2^
MOWOA	2.1174 × 10^−2^	3.5089 × 10^−4^	2.5020 × 10^−1^	1.9044 × 10^−3^	6.2691 × 10^−1^	1.9594 × 10^−1^
MORBMO	3.0910 × 10^−2^	9.5493 × 10^−4^	2.6872 × 10^−1^	1.0967 × 10^−1^	2.8815 × 10^−1^	6.7550 × 10^−2^
MOWMA	2.2517 × 10^−2^	4.9484 × 10^−4^	**1.4765 × 10^−1^**	3.4531 × 10^−3^	8.6450 × 10^−1^	6.7837 × 10^−2^
SMS-EMOA	**1.9967 × 10^−3^**	1.9942 × 10^−4^	2.4551 × 10^−1^	1.0169 × 10^−1^	2.7216 × 10^−1^	3.6669 × 10^−2^
MOEAD	7.3919 × 10^−2^	4.4355 × 10^−2^	5.8925 × 10^−1^	5.8998 × 10^−2^	6.8591 × 10^−1^	1.5424 × 10^−1^
SPEA2	2.6066 × 10^−3^	2.2887 × 10^−4^	5.4858 × 10^−1^	9.5468 × 10^−1^	3.0824 × 10^−1^	7.2555 × 10^−2^
NSGA-II	2.6223 × 10^−3^	2.8912 × 10^−4^	3.8975 × 10^−1^	4.7895 × 10^−1^	3.0201 × 10^−1^	8.9267 × 10^−2^
NSGA-III	2.0082 × 10^−3^	**1.4970 × 10^−6^**	2.2594 × 10^−1^	8.7092 × 10^−2^	2.7103 × 10^−1^	5.2974 × 10^−2^
	CF4		CF5		CF6	
Algorithms	Ave	Std	Ave	Std	Ave	Std
SMOWMA	1.0032 × 10^−1^	**1.1120 × 10^−3^**	**2.3364 × 10^−1^**	4.3943 × 10^−2^	3.0406 × 10^−1^	4.6379 × 10^−4^
MOSFOA	**9.8337 × 10^−2^**	1.8225 × 10^−3^	2.5725 × 10^−1^	3.7340 × 10^−2^	3.0558 × 10^−1^	8.4297 × 10^−4^
MOWOA	1.1228 × 10^−1^	7.8443 × 10^−3^	6.2036 × 10^−1^	2.4248 × 10^−1^	3.0461 × 10^−1^	3.9655 × 10^−4^
MORBMO	1.0060 × 10^−1^	1.2753 × 10^−3^	3.9236 × 10^−1^	6.5011 × 10^−2^	3.0236 × 10^−1^	3.0360 × 10^−4^
MOWMA	1.0403 × 10^−1^	2.7818 × 10^−3^	5.9232 × 10^−1^	**1.5415 × 10^−2^**	3.0987 × 10^−1^	1.2247 × 10^−3^
SMS-EMOA	1.0284 × 10^−1^	3.6468 × 10^−3^	3.3353 × 10^−1^	9.8001 × 10^−2^	3.0231 × 10^−1^	4.2800 × 10^−4^
MOEAD	1.4727 × 10^−1^	2.3183 × 10^−2^	6.9361 × 10^−1^	1.5417 × 10^−1^	3.0581 × 10^−1^	1.7505 × 10^−3^
SPEA2	9.9279 × 10^−2^	1.9484 × 10^−3^	3.6295 × 10^−1^	5.0702 × 10^−2^	**3.0193 × 10^−1^**	**2.4808 × 10^−4^**
NSGA-II	9.9519 × 10^−2^	2.0700 × 10^−3^	3.6813 × 10^−1^	6.1624 × 10^−2^	3.0211 × 10^−1^	3.0879 × 10^−4^
NSGA-III	1.0254 × 10^−1^	4.6030 × 10^−3^	3.1219 × 10^−1^	4.2692 × 10^−2^	3.0417 × 10^−1^	1.8861 × 10^−3^
	CF7		CF8		CF9	
Algorithms	Ave	Std	Ave	Std	Ave	Std
SMOWMA	**3.3953 × 10^−1^**	2.4246 × 10^−2^	5.7660 × 10^−1^	**4.5448 × 10^−8^**	4.8217 × 10^−2^	1.9193 × 10^−3^
MOSFOA	3.4654 × 10^−1^	**1.6532 × 10^−2^**	**2.3634 × 10^−1^**	1.3546 × 10^−1^	9.1021 × 10^−2^	5.2714 × 10^−3^
MOWOA	8.2021 × 10^−1^	1.9991 × 10^−1^	6.3327 × 10^−1^	5.4558 × 10^−1^	1.2719 × 10^−1^	5.1384 × 10^−3^
MORBMO	3.8034 × 10^−1^	5.2629 × 10^−2^	5.4419 × 10^−1^	3.4996 × 10^−2^	1.0613 × 10^−1^	1.7800 × 10^−3^
MOWMA	8.9538 × 10^−1^	1.1231 × 10^−1^	6.4368 × 10^0^	3.2583 × 10^0^	1.0336 × 10^−1^	4.2398 × 10^−3^
SMS-EMOA	3.8983 × 10^−1^	3.0017 × 10^−2^	5.2586 × 10^−1^	7.4105 × 10^−2^	6.2969 × 10^−2^	1.2084 × 10^−3^
MOEAD	8.8030 × 10^−1^	2.0897 × 10^−1^	5.5198 × 10^−1^	2.1028 × 10^−2^	9.6002 × 10^−2^	**3.5349 × 10^−4^**
SPEA2	3.6815 × 10^−1^	2.9770 × 10^−2^	8.1219 × 10^−1^	1.1741 × 10^0^	**4.1851 × 10^−2^**	1.3335 × 10^−3^
NSGA-II	4.0039 × 10^−1^	9.1664 × 10^−2^	5.4495 × 10^−1^	2.8936 × 10^−2^	6.3292 × 10^−2^	4.7093 × 10^−3^
NSGA-III	3.7318 × 10^−1^	3.9325 × 10^−2^	5.3358 × 10^−1^	6.7622 × 10^−2^	4.4956 × 10^−2^	1.2454 × 10^−3^
	CF10					
Algorithms	Ave	Std				
SMOWMA	4.5991 × 10^−2^	5.5103 × 10^−3^				
MOSFOA	6.4547 × 10^−2^	4.8485 × 10^−3^				
MOWOA	9.9112 × 10^−2^	3.7921 × 10^−3^				
MORBMO	9.4546 × 10^−2^	3.4630 × 10^−3^				
MOWMA	9.7856 × 10^−2^	7.3541 × 10^−3^				
SMS-EMOA	5.5399 × 10^−2^	9.2096 × 10^−4^				
MOEAD	7.3668 × 10^−2^	1.5842 × 10^−3^				
SPEA2	**3.4938 × 10^−2^**	1.1733 × 10^−3^				
NSGA-II	4.9119 × 10^−2^	3.0179 × 10^−3^				
NSGA-III	3.8001 × 10^−2^	**2.9301 × 10^−5^**				

**Table A3:** Spacing results for the benchmark problems.

	**ZDT1**		**ZDT2**		**ZDT3**	
Algorithms	Ave	Std	Ave	Std	Ave	Std
SMOWMA	**3.0094 × 10^−3^**	**1.7740 × 10^−4^**	2.6874 × 10^−3^	**1.3019 × 10^−4^**	**3.4258 × 10^−3^**	**1.6539 × 10^−4^**
MOSFOA	9.4170 × 10^−3^	2.4720 × 10^−3^	2.1455 × 10^−3^	1.8730 × 10^−3^	3.7555 × 10^−2^	7.2448 × 10^−3^
MOWOA	2.1992 × 10^−2^	6.5561 × 10^−3^	7.9459 × 10^−3^	7.1003 × 10^−4^	4.1045 × 10^−2^	6.9645 × 10^−3^
MORBMO	6.5148 × 10^−3^	9.1794 × 10^−4^	4.1065 × 10^−3^	4.5401 × 10^−4^	1.0239 × 10^−2^	1.9844 × 10^−3^
MOWMA	6.9930 × 10^−3^	2.5300 × 10^−4^	4.6715 × 10^−2^	2.0501 × 10^−2^	9.4425 × 10^−1^	4.0689 × 10^−3^
SMS-EMOA	1.7825 × 10^−2^	2.4459 × 10^−2^	4.1076 × 10^−3^	2.9753 × 10^−3^	1.4029 × 10^−2^	2.6947 × 10^−3^
MOEAD	5.5735 × 10^−2^	6.3180 × 10^−2^	**9.6468 × 10^−4^**	1.3022 × 10^−3^	1.0501 × 10^−1^	1.6797 × 10^−1^
SPEA2	9.1281 × 10^−3^	2.0799 × 10^−3^	5.4668 × 10^−3^	2.0032 × 10^−3^	1.3577 × 10^−2^	3.0037 × 10^−3^
NSGA-II	1.0863 × 10^−2^	2.5625 × 10^−3^	6.1036 × 10^−3^	1.4196 × 10^−3^	1.8556 × 10^−2^	5.5272 × 10^−3^
NSGA-III	1.1447 × 10^−2^	6.7835 × 10^−3^	5.6041 × 10^−3^	4.4271 × 10^−3^	2.2009 × 10^−2^	1.1107 × 10^−2^
	ZDT4		ZDT6		DTLZ1	
Algorithms	Ave	Std	Ave	Std	Ave	Std
SMOWMA	2.6000 × 10^−3^	**1.9273 × 10^−4^**	2.5749 × 10^−3^	**2.0272 × 10^−4^**	9.4572 × 10^−3^	6.9249 × 10^−4^
MOSFOA	**2.1000 × 10^−3^**	7.5565 × 10^−4^	3.0392 × 10^−3^	2.5301 × 10^−4^	3.0218 × 10^−2^	2.0844 × 10^−2^
MOWOA	7.9497 × 10^−3^	1.0637 × 10^−3^	5.5060 × 10^−3^	4.5537 × 10^−4^	1.8386 × 10^−2^	2.1851 × 10^−2^
MORBMO	3.0849 × 10^−1^	1.5003 × 10^−1^	1.2435 × 10^−1^	2.3452 × 10^−1^	2.4232 × 10^0^	2.4598 × 10^0^
MOWMA	1.1642 × 10^0^	6.8349 × 10^−1^	1.3139 × 10^−1^	3.8840 × 10^−2^	1.9305 × 10^0^	1.8767 × 10^−1^
SMS-EMOA	1.4337 × 10^−2^	2.1119 × 10^−2^	3.1879 × 10^−3^	5.1136 × 10^−4^	3.8431 × 10^−3^	1.9531 × 10^−4^
MOEAD	2.9797 × 10^−3^	1.1671 × 10^−3^	**1.3015 × 10^−3^**	2.0321 × 10^−4^	**5.5914 × 10^−5^**	**4.1269 × 10^−5^**
SPEA2	8.6988 × 10^−2^	2.4800 × 10^−1^	4.0356 × 10^−3^	7.0665 × 10^−4^	5.3455 × 10^−3^	9.4897 × 10^−4^
NSGA-II	7.4823 × 10^−3^	2.3461 × 10^−3^	5.0606 × 10^−3^	5.3200 × 10^−4^	2.2745 × 10^−1^	6.8652 × 10^−1^
NSGA-III	1.1793 × 10^−2^	3.0085 × 10^−3^	2.7384 × 10^−3^	5.9968 × 10^−4^	6.1407 × 10^−3^	1.9305 × 10^−2^
	DTLZ2		DTLZ3		DTLZ4	
Algorithms	Ave	Std	Ave	Std	Ave	Std
SMOWMA	**1.1920 × 10^−2^**	4.8155 × 10^−3^	2.6510 × 10^−2^	2.3214 × 10^−3^	2.7748 × 10^−2^	3.2239 × 10^−3^
MOSFOA	2.8003 × 10^−2^	2.2106 × 10^−3^	5.2014 × 10^−1^	4.0011 × 10^−1^	2.8208 × 10^−2^	2.7002 × 10^−3^
MOWOA	3.1457 × 10^−2^	1.0632 × 10^−3^	2.7840 × 10^0^	4.4753 × 10^−1^	2.6636 × 10^−2^	1.7242 × 10^−3^
MORBMO	2.6305 × 10^−2^	1.8107 × 10^−3^	2.5528 × 10^−2^	**1.5504 × 10^−3^**	2.5976 × 10^−2^	1.6288 × 10^−3^
MOWMA	2.7128 × 10^−2^	3.2892 × 10^−3^	1.0861 × 10^1^	1.4602 × 10^0^	2.6464 × 10^−2^	1.5909 × 10^−3^
SMS-EMOA	2.0068 × 10^−2^	5.2147 × 10^−4^	3.9602 × 10^0^	4.9492 × 10^0^	2.0010 × 10^−2^	5.4191 × 10^−4^
MOEAD	1.9125 × 10^−2^	**2.5627 × 10^−6^**	9.9793 × 10^−2^	6.6588 × 10^−2^	1.2688 × 10^−2^	6.9241 × 10^−3^
SPEA2	1.2912 × 10^−2^	1.0232 × 10^−3^	**2.0888 × 10^−2^**	1.8335 × 10^−2^	**1.2020 × 10^−2^**	5.8058 × 10^−4^
NSGA-II	2.6117 × 10^−2^	1.1924 × 10^−3^	4.1429 × 10^−2^	2.3253 × 10^−2^	2.7879 × 10^−2^	1.5026 × 10^−3^
NSGA-III	1.9102 × 10^−2^	8.8466 × 10^−6^	1.4102 × 10^0^	3.3935 × 10^0^	1.9099 × 10^−2^	**2.5141 × 10^−6^**
	DTLZ5		DTLZ6		WFG1	
Algorithms	Ave	Std	Ave	Std	Ave	Std
SMOWMA	3.1102 × 10^−3^	**1.3417 × 10^−4^**	3.5441 × 10^−3^	**2.1479 × 10^−4^**	**3.2865 × 10^−3^**	**1.5906 × 10^−4^**
MOSFOA	3.1296 × 10^−3^	2.7008 × 10^−4^	3.5767 × 10^−3^	5.6239 × 10^−4^	1.9302 × 10^−2^	8.4452 × 10^−3^
MOWOA	3.3351 × 10^−3^	2.4512 × 10^−4^	3.2580 × 10^−3^	2.5108 × 10^−4^	3.4857 × 10^−2^	1.0048 × 10^−2^
MORBMO	3.9978 × 10^−3^	1.9204 × 10^−4^	7.5114 × 10^−3^	1.0243 × 10^−2^	3.8778 × 10^−2^	6.1804 × 10^−3^
MOWMA	7.8655 × 10^−3^	1.0294 × 10^−3^	**2.5742 × 10^−3^**	2.2731 × 10^−4^	3.0293 × 10^−2^	4.8504 × 10^−3^
SMS-EMOA	4.2408 × 10^−3^	2.8155 × 10^−4^	9.1621 × 10^−3^	2.9135 × 10^−3^	3.4048 × 10^−3^	1.3699 × 10^−3^
MOEAD	4.2199 × 10^−3^	1.4584 × 10^−4^	5.3800 × 10^−2^	5.8464 × 10^−2^	5.4392 × 10^−3^	4.0596 × 10^−3^
SPEA2	**2.0610 × 10^−3^**	2.2513 × 10^−4^	6.2343 × 10^−3^	9.1552 × 10^−4^	6.2908 × 10^−3^	1.0911 × 10^−3^
NSGA-II	3.4397 × 10^−3^	2.3241 × 10^−4^	7.8002 × 10^−3^	1.6792 × 10^−3^	7.8859 × 10^−3^	1.6043 × 10^−3^
NSGA-III	4.8455 × 10^−2^	3.6430 × 10^−3^	3.8742 × 10^−2^	4.0254 × 10^−3^	4.9936 × 10^−3^	1.2178 × 10^−3^
	WFG2		WFG3		WFG4	
Algorithms	Ave	Std	Ave	Std	Ave	Std
SMOWMA	8.1604 × 10^−3^	4.9117 × 10^−4^	8.6108 × 10^−3^	9.7621 × 10^−4^	**7.8280 × 10^−3^**	7.6109 × 10^−4^
MOSFOA	5.3023 × 10^−3^	3.7438 × 10^−4^	**5.2485 × 10^−3^**	4.3741 × 10^−4^	9.3888 × 10^−3^	6.2488 × 10^−4^
MOWOA	5.3732 × 10^−3^	**3.0207 × 10^−4^**	5.8412 × 10^−3^	4.8326 × 10^−4^	2.5576 × 10^−2^	3.9403 × 10^−3^
MORBMO	5.4815 × 10^−3^	5.6316 × 10^−4^	5.6019 × 10^−3^	5.8478 × 10^−4^	2.8069 × 10^−2^	9.5980 × 10^−3^
MOWMA	9.9400 × 10^−3^	1.5049 × 10^−3^	6.6456 × 10^−3^	3.5494 × 10^−4^	1.7915 × 10^−2^	5.4129 × 10^−3^
SMS-EMOA	4.9399 × 10^−3^	5.7587 × 10^−4^	5.6256 × 10^−3^	5.2373 × 10^−4^	3.1047 × 10^−2^	5.0669 × 10^−3^
MOEAD	5.7239 × 10^−3^	7.0620 × 10^−4^	6.5318 × 10^−3^	4.0906 × 10^−4^	1.0337 × 10^−2^	9.7296 × 10^−4^
SPEA2	5.6419 × 10^−3^	1.2985 × 10^−3^	6.5187 × 10^−3^	4.6258 × 10^−4^	1.8308 × 10^−2^	1.8459 × 10^−3^
NSGA-II	**4.5739 × 10^−3^**	5.0932 × 10^−4^	5.9837 × 10^−3^	4.9344 × 10^−4^	2.3801 × 10^−2^	1.3725 × 10^−3^
NSGA-III	5.3198 × 10^−3^	4.9101 × 10^−4^	6.6326 × 10^−3^	**2.7024 × 10^−4^**	1.8940 × 10^−2^	**5.7636 × 10^−4^**
	WFG5		WFG6		WFG7	
Algorithms	Ave	Std	Ave	Std	Ave	Std
SMOWMA	**7.1765 × 10^−3^**	4.9590 × 10^−4^	**7.2619 × 10^−3^**	3.0978 × 10^−4^	1.1795 × 10^−2^	4.3544 × 10^−3^
MOSFOA	1.0451 × 10^−2^	1.3726 × 10^−3^	8.9787 × 10^−3^	4.7142 × 10^−4^	9.1518 × 10^−3^	9.2393 × 10^−4^
MOWOA	2.4388 × 10^−2^	2.9010 × 10^−3^	2.6331 × 10^−2^	2.2049 × 10^−3^	2.5595 × 10^−2^	2.5740 × 10^−3^
MORBMO	1.5080 × 10^−2^	9.5032 × 10^−4^	1.4341 × 10^−2^	9.1699 × 10^−4^	1.6237 × 10^−2^	1.7843 × 10^−3^
MOWMA	8.3604 × 10^−3^	4.8899 × 10^−4^	8.4464 × 10^−3^	1.9304 × 10^−3^	**8.8169 × 10^−3^**	9.3326 × 10^−4^
SMS-EMOA	3.2723 × 10^−2^	3.6371 × 10^−3^	3.4818 × 10^−2^	2.9073 × 10^−3^	3.3494 × 10^−2^	3.2496 × 10^−3^
MOEAD	9.9609 × 10^−3^	4.0473 × 10^−4^	1.0178 × 10^−2^	5.3945 × 10^−4^	1.0596 × 10^−2^	1.6101 × 10^−3^
SPEA2	1.6675 × 10^−2^	2.5743 × 10^−3^	1.7411 × 10^−2^	1.4022 × 10^−3^	1.8208 × 10^−2^	2.1765 × 10^−3^
NSGA-II	2.3260 × 10^−2^	1.5351 × 10^−3^	2.9058 × 10^−2^	6.3269 × 10^−3^	2.5727 × 10^−2^	3.1691 × 10^−3^
NSGA-III	1.8917 × 10^−2^	**3.2568 × 10^−4^**	1.8914 × 10^−2^	**1.9277 × 10^−4^**	1.8786 × 10^−2^	**5.9659 × 10^−4^**
	WFG8		WFG9		UF1	
Algorithms	Ave	Std	Ave	Std	Ave	Std
SMOWMA	8.4098 × 10^−3^	**4.6529 × 10^−4^**	8.9156 × 10^−3^	8.4152 × 10^−4^	1.5131 × 10^−3^	**1.9445 × 10^−4^**
MOSFOA	3.2590 × 10^−2^	3.4939 × 10^−3^	7.1536 × 10^−3^	3.9447 × 10^−4^	9.8850 × 10^−3^	1.1217 × 10^−2^
MOWOA	**7.2219 × 10^−3^**	1.2934 × 10^−3^	**6.8886 × 10^−3^**	7.6254 × 10^−4^	**1.1683 × 10^−3^**	6.0695 × 10^−4^
MORBMO	1.5264 × 10^−2^	4.6492 × 10^−3^	7.1569 × 10^−3^	4.3889 × 10^−4^	6.5467 × 10^−3^	1.8060 × 10^−3^
MOWMA	3.4430 × 10^−2^	4.3810 × 10^−2^	7.6054 × 10^−3^	4.4368 × 10^−4^	3.1097 × 10^−2^	1.1584 × 10^−2^
SMS-EMOA	1.1024 × 10^−2^	7.5990 × 10^−3^	7.3115 × 10^−3^	4.0699 × 10^−4^	3.2893 × 10^−3^	8.2949 × 10^−3^
MOEAD	7.7249 × 10^−3^	7.8569 × 10^−4^	1.4845 × 10^−2^	3.9582 × 10^−3^	1.1759 × 10^−2^	1.3648 × 10^−2^
SPEA2	7.5818 × 10^−3^	1.2594 × 10^−3^	7.7266 × 10^−3^	1.3056 × 10^−3^	4.6142 × 10^−3^	1.0512 × 10^−2^
NSGA-II	7.6831 × 10^−3^	1.3649 × 10^−3^	7.1369 × 10^−3^	**3.5986 × 10^−4^**	4.0568 × 10^−3^	9.1567 × 10^−3^
NSGA-III	8.7049 × 10^−3^	1.8992 × 10^−3^	9.2479 × 10^−3^	5.0358 × 10^−4^	3.3604 × 10^−3^	2.5346 × 10^−3^
	UF2		UF3		UF4	
Algorithms	Ave	Std	Ave	Std	Ave	Std
SMOWMA	**2.4272 × 10^−3^**	**2.5350 × 10^−4^**	2.2420 × 10^−3^	1.1304 × 10^−3^	**8.5851 × 10^−4^**	**1.8619 × 10^−4^**
MOSFOA	5.1790 × 10^−3^	2.6606 × 10^−3^	6.1185 × 10^−3^	1.1704 × 10^−3^	7.8303 × 10^−3^	6.0969 × 10^−3^
MOWOA	4.6705 × 10^−3^	2.9458 × 10^−3^	4.6521 × 10^−3^	3.2362 × 10^−3^	1.1212 × 10^−3^	1.4621 × 10^−3^
MORBMO	2.5818 × 10^−3^	4.3108 × 10^−4^	8.0019 × 10^−3^	5.3402 × 10^−3^	7.6408 × 10^−3^	4.6269 × 10^−3^
MOWMA	1.7271 × 10^−2^	6.8907 × 10^−3^	3.8689 × 10^−1^	3.8476 × 10^−1^	5.2134 × 10^−2^	3.0763 × 10^−2^
SMS-EMOA	5.9768 × 10^−3^	2.0937 × 10^−3^	8.0673 × 10^−3^	1.1667 × 10^−2^	1.4371 × 10^−2^	1.9155 × 10^−2^
MOEAD	2.5177 × 10^−3^	1.6800 × 10^−3^	**4.7751 × 10^−5^**	**1.4861 × 10^−4^**	1.3199 × 10^−2^	9.7545 × 10^−3^
SPEA2	4.3767 × 10^−3^	3.6266 × 10^−3^	1.4611 × 10^−2^	1.1990 × 10^−2^	9.5966 × 10^−3^	9.3060 × 10^−3^
NSGA-II	3.2188 × 10^−3^	1.3462 × 10^−3^	4.0212 × 10^−3^	4.2178 × 10^−3^	2.3107 × 10^−3^	4.1416 × 10^−3^
NSGA-III	9.5272 × 10^−3^	2.1541 × 10^−3^	3.2927 × 10^−2^	2.4078 × 10^−2^	1.4802 × 10^−1^	1.5021 × 10^−1^
	UF5		UF6		UF7	
Algorithms	Ave	Std	Ave	Std	Ave	Std
SMOWMA	**1.3720 × 10^−4^**	**9.0560 × 10^−5^**	7.7560 × 10^−4^	4.8154 × 10^−4^	2.0682 × 10^−3^	**1.5988 × 10^−4^**
MOSFOA	2.2245 × 10^−2^	1.5993 × 10^−2^	1.0799 × 10^−2^	6.1170 × 10^−3^	3.8477 × 10^−3^	1.0769 × 10^−3^
MOWOA	1.9593 × 10^−3^	1.8316 × 10^−3^	5.5502 × 10^−3^	5.7157 × 10^−3^	**1.6577 × 10^−3^**	1.6379 × 10^−4^
MORBMO	5.0351 × 10^−2^	2.1164 × 10^−2^	1.1779 × 10^−2^	4.4615 × 10^−3^	2.5279 × 10^−3^	6.4139 × 10^−4^
MOWMA	1.7746 × 10^−1^	1.0395 × 10^−1^	1.1186 × 10^−1^	7.0171 × 10^−2^	3.3187 × 10^−2^	2.5879 × 10^−2^
SMS-EMOA	1.0248 × 10^−2^	2.3534 × 10^−2^	5.9544 × 10^−3^	1.8215 × 10^−2^	8.4230 × 10^−3^	1.6474 × 10^−2^
MOEAD	4.8161 × 10^−3^	7.3161 × 10^−3^	1.2026 × 10^−2^	2.7679 × 10^−2^	5.4388 × 10^−3^	9.9251 × 10^−3^
SPEA2	5.7050 × 10^−4^	1.3715 × 10^−3^	**9.6794 × 10^−5^**	**2.0680 × 10^−4^**	8.8319 × 10^−3^	9.0961 × 10^−3^
NSGA-II	2.8322 × 10^−3^	5.6020 × 10^−3^	8.9776 × 10^−3^	2.0395 × 10^−2^	1.3276 × 10^−2^	3.1060 × 10^−2^
NSGA-III	8.9989 × 10^−2^	1.9387 × 10^−1^	9.8539 × 10^−2^	1.3372 × 10^−1^	2.7331 × 10^−2^	2.5822 × 10^−2^
	CF1		CF2		CF3	
Algorithms	Ave	Std	Ave	Std	Ave	Std
SMOWMA	3.2344 × 10^−3^	1.6166 × 10^−3^	1.5202 × 10^−1^	1.3703 × 10^−2^	**4.6343 × 10^−3^**	4.3025 × 10^−3^
MOSFOA	1.4557 × 10^−2^	3.1828 × 10^−3^	1.1532 × 10^−3^	4.9270 × 10^−4^	1.4076 × 10^−2^	1.4695 × 10^−2^
MOWOA	7.5326 × 10^−3^	5.5344 × 10^−3^	**9.3678 × 10^−4^**	**3.6094 × 10^−4^**	1.0253 × 10^−2^	8.2100 × 10^−3^
MORBMO	5.2288 × 10^−3^	2.9930 × 10^−3^	4.3324 × 10^−3^	1.0810 × 10^−2^	5.3241 × 10^−3^	**9.3578 × 10^−4^**
MOWMA	1.4343 × 10^−2^	1.3454 × 10^−2^	7.9740 × 10^−2^	5.3622 × 10^−2^	3.1242 × 10^−1^	1.6116 × 10^−1^
SMS-EMOA	9.7371 × 10^−4^	7.5059 × 10^−5^	1.1817 × 10^−2^	1.5259 × 10^−2^	1.9636 × 10^−2^	1.9163 × 10^−2^
MOEAD	1.9569 × 10^−3^	3.4704 × 10^−3^	1.1503 × 10^−1^	1.2845 × 10^−2^	5.1193 × 10^−2^	1.0446 × 10^−1^
SPEA2	1.6832 × 10^−3^	1.2439 × 10^−4^	1.4685× 10^−2^	5.3576 × 10^−3^	2.7381 × 10^−2^	3.2328 × 10^−2^
NSGA-II	2.7150 × 10^−3^	1.8398 × 10^−4^	1.8546 × 10^−2^	4.7245 × 10^−2^	1.3754 × 10^−2^	1.8716 × 10^−2^
NSGA-III	**7.4025 × 10^−6^**	**2.2999 × 10^−6^**	1.1356 × 10^−1^	4.4167 × 10^−2^	2.1932 × 10^−1^	1.7263 × 10^−1^
	CF4		CF5		CF6	
Algorithms	Ave	Std	Ave	Std	Ave	Std
SMOWMA	**1.7163 × 10^−3^**	2.1243 × 10^−3^	**8.3991 × 10^−4^**	**1.1346 × 10^−3^**	8.4412 × 10^−3^	1.8092 × 10^−2^
MOSFOA	2.4900 × 10^−3^	**1.4360 × 10^−3^**	1.1420 × 10^−2^	8.9608 × 10^−3^	9.7091 × 10^−3^	5.7386 × 10^−3^
MOWOA	4.4245 × 10^−3^	7.7614 × 10^−3^	2.2295 × 10^−3^	3.5887 × 10^−3^	5.7667 × 10^−3^	2.4714 × 10^−3^
MORBMO	1.8456 × 10^−3^	2.2374 × 10^−3^	1.4097 × 10^−2^	2.9032 × 10^−2^	**2.5638 × 10^−3^**	3.3677 × 10^−3^
MOWMA	2.3133 × 10^−2^	8.9229 × 10^−3^	3.6538 × 10^−1^	2.5858 × 10^−1^	3.6335 × 10^−2^	1.7646 × 10^−2^
SMS-EMOA	9.3874 × 10^−3^	8.1714 × 10^−3^	3.7023 × 10^−2^	2.6327 × 10^−2^	7.8722 × 10^−3^	5.2221 × 10^−3^
MOEAD	1.9157 × 10^−2^	1.4728 × 10^−2^	2.9050 × 10^−2^	5.9639 × 10^−2^	6.3445 × 10^−3^	**2.1840 × 10^−3^**
SPEA2	1.6390 × 10^−2^	9.2173 × 10^−3^	3.8604 × 10^−2^	3.8903 × 10^−2^	8.9896 × 10^−3^	6.3423 × 10^−3^
NSGA-II	3.4793 × 10^−3^	3.8557 × 10^−3^	2.3431 × 10^−2^	3.8952 × 10^−2^	8.1418 × 10^−3^	6.0041 × 10^−3^
NSGA-III	4.7774 × 10^−2^	1.5258 × 10^−2^	2.7456 × 10^−1^	1.8237 × 10^−1^	5.4514 × 10^−2^	3.7440 × 10^−2^
	CF7		CF8		CF9	
Algorithms	Ave	Std	Ave	Std	Ave	Std
SMOWMA	6.5052 × 10^−3^	**2.8621 × 10^−3^**	**7.5848 × 10^−2^**	**3.4460 × 10^−2^**	2.7595 × 10^−2^	1.3868 × 10^−3^
MOSFOA	2.5583 × 10^−2^	2.7211 × 10^−2^	2.8253 × 10^−1^	1.1950 × 10^−1^	9.9238 × 10^−2^	4.8636 × 10^−2^
MOWOA	4.4520 × 10^−3^	5.2765 × 10^−3^	8.5100 × 10^−1^	8.3824 × 10^−1^	4.0161 × 10^−2^	2.4140 × 10^−3^
MORBMO	**3.9377 × 10^−3^**	7.8561 × 10^−3^	3.2242 × 10^−1^	2.9656 × 10^−1^	3.3387 × 10^−2^	4.0413 × 10^−3^
MOWMA	5.6718 × 10^−1^	3.2467 × 10^−1^	1.6249 × 10^1^	8.3916 × 10^0^	4.1407 × 10^−2^	6.3640 × 10^−3^
SMS-EMOA	1.9775 × 10^−2^	1.4160 × 10^−2^	7.1412 × 10^−1^	1.0823 × 10^0^	3.0264 × 10^−2^	**4.5239 × 10^−4^**
MOEAD	7.9523 × 10^−3^	6.0671 × 10^−3^	9.3125 × 10^−2^	9.2304 × 10^−2^	2.8844 × 10^−2^	1.2982 × 10^−3^
SPEA2	1.9259 × 10^−2^	1.8638 × 10^−2^	1.9408 × 10^0^	3.9705 × 10^0^	1.7530 × 10^−2^	4.7142 × 10^−3^
NSGA-II	4.9024 × 10^−2^	6.1757 × 10^−2^	1.6185 × 10^0^	2.1717 × 10^0^	3.2098 × 10^−2^	2.7039 × 10^−3^
NSGA-III	2.1373 × 10^−1^	1.6173 × 10^−1^	8.0846 × 10^−1^	1.9448 × 10^0^	**1.7383 × 10^−2^**	7.9012 × 10^−4^
	CF10					
Algorithms	Ave	Std				
SMOWMA	2.4642 × 10^−2^	4.9366 × 10^−3^				
MOSFOA	2.9422 × 10^−2^	6.2575 × 10^−3^				
MOWOA	3.0820 × 10^−2^	5.4561 × 10^−3^				
MORBMO	2.4815 × 10^−2^	3.9761 × 10^−3^				
MOWMA	2.8423 × 10^−2^	7.2144 × 10^−3^				
SMS-EMOA	3.2739 × 10^−2^	4.6132 × 10^−3^				
MOEAD	2.4386 × 10^−2^	**1.6930 × 10^−3^**				
SPEA2	2.0511 × 10^−2^	8.9547 × 10^−3^				
NSGA-II	2.4115 × 10^−2^	2.3931 × 10^−3^				
NSGA-III	**2.0024 × 10^−2^**	5.4442 × 10^−3^				

**Table A4:** HV results of the SMOWMA ablation study.

	**ZDT1**		**ZDT2**		**ZDT3**	
Algorithms	Avg	Std	Avg	Std	Avg	Std
SMOWMA	**8.6656 × 10^−1^**	**6.6818 × 10^−4^**	**5.4062 × 10^−1^**	**8.8848 × 10^−5^**	**1.0244 × 10^0^**	**5.3845 × 10^−5^**
SMOWMA-RI	8.4648 × 10^−1^	1.5510 × 10^−3^	1.0357 × 10^−1^	1.2170 × 10^−3^	9.8226 × 10^−1^	3.9194 × 10^−3^
SMOWMA-Sobol	8.4624 × 10^−1^	1.0261 × 10^−3^	1.0405 × 10^−1^	6.2000 × 10^−4^	9.8224 × 10^−1^	6.9600 × 10^−3^
SMOWMA-LF	8.3706 × 10^−1^	1.9326 × 10^−3^	1.4220 × 10^−1^	1.2270 × 10^−1^	9.6563 × 10^−1^	5.6510 × 10^−3^
SMOWMA-FixedT	8.4463 × 10^−1^	1.4664 × 10^−3^	2.6199 × 10^−1^	2.0404 × 10^−1^	9.7869 × 10^−1^	4.8718 × 10^−3^
SMOWMA-Uniform	8.4454 × 10^−1^	1.7256 × 10^−3^	1.4402 × 10^−1^	1.2585 × 10^−1^	9.5737 × 10^−1^	9.6148 × 10^−3^
SMOWMA-NoRho	8.4751 × 10^−1^	1.5739 × 10^−3^	2.9401 × 10^−1^	2.2531 × 10^−1^	9.7686 × 10^−1^	8.2000 × 10^−3^
SMOWMA-NoAG	8.4192 × 10^−1^	2.1211 × 10^−3^	1.8072 × 10^−1^	1.6519 × 10^−1^	9.5087 × 10^−1^	1.0765 × 10^−2^
	DTLZ2		DTLZ4		DTLZ5	
Algorithms	Avg	Std	Avg	Std	Avg	Std
SMOWMA	**7.9645 × 10^−1^**	**2.7761 × 10^−3^**	**7.8969 × 10^−1^**	**4.6511 × 10^−3^**	**1.3345 × 10^−1^**	**4.0362 × 10^−5^**
SMOWMA-RI	6.8909 × 10^−1^	3.1600 × 10^−3^	6.3231 × 10^−1^	1.8005 × 10^−1^	1.3196 × 10^−1^	2.4900 × 10^−4^
SMOWMA-Sobol	6.9532 × 10^−1^	6.0485 × 10^−3^	6.9714 × 10^−1^	1.4869 × 10^−2^	1.3190 × 10^−1^	2.6739 × 10^−4^
SMOWMA-LF	6.8537 × 10^−1^	9.6942 × 10^−3^	6.9016 × 10^−1^	8.0971 × 10^−3^	1.3186 × 10^−1^	1.8101 × 10^−4^
SMOWMA-FixedT	6.8916 × 10^−1^	9.2711 × 10^−3^	6.9959 × 10^−1^	7.3568 × 10^−3^	1.3187 × 10^−1^	1.8471 × 10^−4^
SMOWMA-Uniform	6.8970 × 10^−1^	6.9234 × 10^−3^	7.0262 × 10^−1^	7.9223 × 10^−3^	1.3187 × 10^−1^	3.9962 × 10^−4^
SMOWMA-NoRho	6.9219 × 10^−1^	8.1365 × 10^−3^	6.9086 × 10^−1^	9.8668 × 10^−3^	1.3175 × 10^−1^	6.0214 × 10^−4^
SMOWMA-NoAG	6.9714 × 10^−1^	5.6941 × 10^−3^	6.9264 × 10^−1^	9.5195 × 10^−3^	1.3186 × 10^−1^	1.4035 × 10^−4^
	WFG2		WFG3		WFG5	
Algorithms	Avg	Std	Avg	Std	Avg	Std
SMOWMA	**6.1487 × 10^0^**	**6.9631 × 10^−4^**	**5.6269 × 10^0^**	**1.6305 × 10^−3^**	**2.9612 × 10^0^**	**5.5142 × 10^−3^**
SMOWMA-RI	6.0676 × 10^0^	5.6915 × 10^−2^	5.5542 × 10^0^	8.4750 × 10^−3^	2.8329 × 10^0^	2.8525 × 10^−2^
SMOWMA-Sobol	6.0793 × 10^0^	5.8422 × 10^−2^	5.5545 × 10^0^	8.8871 × 10^−3^	2.8445 × 10^0^	1.4718 × 10^−2^
SMOWMA-LF	6.0697 × 10^0^	5.5282 × 10^−2^	5.5510 × 10^0^	7.3343 × 10^−3^	2.8373 × 10^0^	1.0894 × 10^−2^
SMOWMA-FixedT	6.0718 × 10^0^	5.9917 × 10^−2^	5.5560 × 10^0^	4.0304 × 10^−3^	2.8384 × 10^0^	1.6471 × 10^−2^
SMOWMA-Uniform	5.9437 × 10^0^	9.4755 × 10^−2^	5.5544 × 10^0^	5.9285 × 10^−3^	2.8381 × 10^0^	1.7908 × 10^−2^
SMOWMA-NoRho	6.0771 × 10^0^	5.5417 × 10^−2^	5.5522 × 10^0^	8.9063 × 10^−3^	2.8481 × 10^0^	1.4451 × 10^−2^
SMOWMA-NoAG	5.9396 × 10^0^	8.2554 × 10^−2^	5.5509 × 10^0^	8.0047 × 10^−3^	2.8437 × 10^0^	1.4304 × 10^−2^
	UF3		UF4		UF6	
Algorithms	Avg	Std	Avg	Std	Avg	Std
SMOWMA	**7.3294 × 10^−1^**	**1.7805 × 10^−2^**	**4.3136 × 10^−1^**	**6.8336 × 10^−3^**	**4.7012 × 10^−1^**	**3.9800 × 10^−3^**
SMOWMA-RI	4.5053 × 10^−1^	3.3938 × 10^−2^	3.3133 × 10^−1^	5.6257 × 10^−2^	1.9769 × 10^−1^	1.1108 × 10^−1^
SMOWMA-Sobol	4.5891 × 10^−1^	3.0119 × 10^−2^	3.0984 × 10^−1^	5.3188 × 10^−2^	1.1571 × 10^−1^	1.2437 × 10^−1^
SMOWMA-LF	4.4424 × 10^−1^	2.1918 × 10^−2^	3.1644 × 10^−1^	7.1681 × 10^−2^	1.6795 × 10^−1^	1.0973 × 10^−1^
SMOWMA-FixedT	4.5922 × 10^−1^	2.4722 × 10^−2^	2.9475 × 10^−1^	6.4554 × 10^−2^	1.4820 × 10^−1^	1.2615 × 10^−1^
SMOWMA-Uniform	4.5290 × 10^−1^	3.6506 × 10^−2^	3.4089 × 10^−1^	3.7472 × 10^−2^	1.3139 × 10^−1^	1.2591 × 10^−1^
SMOWMA-NoRho	4.5445 × 10^−1^	2.9547 × 10^−2^	2.5388 × 10^−1^	9.6823 × 10^−2^	1.5044 × 10^−1^	1.4153 × 10^−1^
SMOWMA-NoAG	4.6596 × 10^−1^	2.1822 × 10^−2^	3.6635 × 10^−1^	2.0292 × 10^−2^	2.3590 × 10^−1^	8.9227 × 10^−2^
	CF2		CF5		CF7	
Algorithms	Avg	Std	Avg	Std	Avg	Std
SMOWMA	**7.7501 × 10^−1^**	1.5322 × 10^−2^	**2.5921 × 10^−1^**	9.8494 × 10^−2^	**3.1242 × 10^−1^**	7.3084 × 10^−2^
SMOWMA-RI	5.4857 × 10^−1^	2.5241 × 10^−2^	2.5256 × 10^−2^	4.5388 × 10^−2^	1.7608 × 10^−2^	5.5683 × 10^−2^
SMOWMA-Sobol	5.6443 × 10^−1^	2.1282 × 10^−2^	2.5138 × 10^−2^	4.6062 × 10^−2^	4.0762 × 10^−3^	**1.2890 × 10^−2^**
SMOWMA-LF	5.5944 × 10^−1^	2.0914 × 10^−2^	4.1769 × 10^−2^	4.7850 × 10^−2^	1.1161 × 10^−2^	2.0528 × 10^−2^
SMOWMA-FixedT	5.5860 × 10^−1^	2.6901 × 10^−2^	2.6646 × 10^−2^	**3.7160 × 10^−2^**	1.6208 × 10^−2^	3.1884 × 10^−2^
SMOWMA-Uniform	5.8855 × 10^−1^	1.1075 × 10^−2^	4.1356 × 10^−2^	6.0444 × 10^−2^	3.0506 × 10^−2^	3.7064 × 10^−2^
SMOWMA-NoRho	5.4363 × 10^−1^	4.1271 × 10^−2^	3.3722 × 10^−2^	5.4360 × 10^−2^	5.2389 × 10^−3^	1.5724 × 10^−2^
SMOWMA-NoAG	5.9058 × 10^−1^	**1.0389 × 10^−2^**	6.3648 × 10^−2^	5.2811 × 10^−2^	2.2627 × 10^−2^	3.1338 × 10^−2^
